# Dissection of the antimicrobial and hemolytic activity of Cap18: Generation of Cap18 derivatives with enhanced specificity

**DOI:** 10.1371/journal.pone.0197742

**Published:** 2018-05-31

**Authors:** Anna Ebbensgaard, Hanne Mordhorst, Michael Toft Overgaard, Frank Møller Aarestrup, Egon Bech Hansen

**Affiliations:** 1 National Food Institute, Technical University of Denmark, Kongens Lyngby, Denmark; 2 Department of Biology, University of Copenhagen, Copenhagen, Denmark; 3 Department of Chemistry and Bioscience, Aalborg University, Aalborg, Denmark; Nagoya University, JAPAN

## Abstract

Due to the rapid emergence of resistance to classical antibiotics, novel antimicrobial compounds are needed. It is desirable to selectively kill pathogenic bacteria without targeting other beneficial bacteria in order to prevent the negative clinical consequences caused by many broad-spectrum antibiotics as well as reducing the development of antibiotic resistance. Antimicrobial peptides (AMPs) represent an alternative to classical antibiotics and it has been previously demonstrated that Cap18 has high antimicrobial activity against a broad range of bacterial species. In this study we report the design of a positional scanning library consisting of 696 Cap18 derivatives and the subsequent screening for antimicrobial activity against *Y*. *ruckeri*, *A*. *salmonicida*, *S*. Typhimurium and *L*. *lactis* as well as for hemolytic activity measuring the hemoglobin release of horse erythrocytes. We show that the hydrophobic face of Cap18, in particular I13, L17 and I24, is essential for its antimicrobial activity against *S*. Typhimurium, *Y*. *ruckeri*, *A*. *salmonicida*, *E*. *coli*, *P*. *aeruginosa*, *L*. *lactis*, *L*. *monocytogenes* and *E*. *faecalis*. In particular, Cap18 derivatives harboring a I13D, L17D, L17P, I24D or I24N substitution lost their antimicrobial activity against any of the tested bacterial strains. In addition, we were able to generate species-specific Cap18 derivatives by particular amino acid substitutions either in the hydrophobic face at positions L6, L17, I20, and I27, or in the hydrophilic face at positions K16 and K18. Finally, our data showed the proline residue at position 29 to be essential for the inherent low hemolytic activity of Cap18 and that substitution of the residues K16, K23, or G21 by any hydrophobic residues enhances the hemolytic activity. This study demonstrates the potential of generating species-specific AMPs for the selective elimination of bacterial pathogens.

## Introduction

In recent years, the widespread use of antibiotics has contributed to the selection for microorganisms with antibiotic resistance and to the selection for transmission of antibiotic resistance mechanisms between quite distantly related organisms. The number of resistant superbugs is increasing and new anti-infective solutions are urgently needed. However, the time for development of a new antimicrobial drug is quite lengthy compared to the current dissemination of novel antibiotic resistance mechanisms among commensal and pathogenic microorganisms [1–4]. We are therefore in a state of extreme vulnerability with a risk for reappearance of epidemics of infectious diseases.

The use of traditional antibiotics not only select for resistance in a broad range of pathogens, but also disturbs and alters the natural flora, which plays an important role in human and animal health [5–8]. This is one reason behind major health problems/antibiotics-associated infections such as *Clostridium difficile* infections in humans and animals resulting in severe and sometimes fatal enteric diseases [9]. More recent studies have shown that traditional antibiotic treatment can perhaps have lifelong negative consequences due to the permanent disturbance of the microbiota. Therefore, much of the current research in infection therapy is aiming at identifying alternatives which are safer, more specific for a given bacteria target to reduce or eliminate such deleterious side effects on the microbiota and are more sustainable than the traditional antibiotics. New and non-conventional antimicrobials with species-specific killing capability are needed to prevent posttreatment complications and to overcome a future “post-antibiotic era”.

Antimicrobial peptides (AMPs) captured attention and might present an attractive alternative to classical antibiotics. AMPs have been found in all kingdoms of life and are part of the innate immunity and represent the first line of defense in an infection [10,11]. Despite their diversity in origin and sequence, they generally have a substantial proportion of hydrophobic amino acids (= >30%), an overall positive charge (+2 to +11) and are relatively short consisting of 10–50 amino acids [12]. Based on these properties, AMPs are able to fold into amphiphilic three-dimensional structures and are often based on their secondary structure categorized into α-helical, β-sheet or peptides with extended/random coil structure. Most of the so far characterized AMPs belong to the family of the α-helical or β-sheet peptides [13,14].

It is widely accepted that the bacterial membrane is the key component for the antimicrobial activity of AMPs. Based on a considerable amount of *in vitro* data showing the disruption of lipid bilayers by AMPs, it has been suggested that the bactericidal effect of AMPs is mainly due the formation of pores in the cytoplasmic membrane disrupting the physical integrity of bacterial membrane which finally leads to cell death [12,14,15]. However, the exact mechanism of pore formation in bacterial membrane is less certain. It is widely believed that electrostatic forces between the positively charged amino acids of the AMPs and the negatively charged bacterial surface are the initial step and critical determinants for interactions between AMPs and the bacterial membrane. In addition to the negatively charged phospholipids such as phosphatidylglycerol, cardiolipin and phosphatidylserine present in the bacterial cytoplasmic membrane, lipopolysaccharides (LPS) in the outer membrane of Gram-negative bacteria and teichoic acids in the peptidoglycan layer of Gram-positive bacteria are contributing to the overall negative charge of the bacterial cell envelope [16]. The positively charged AMP is expected to accumulate on the surface of the membrane and upon reaching a certain threshold the AMP might self-assemble and incorporate into the membrane by creating a pore. Several models for pore formation of AMPs have been suggested including the barrel-stave model, the carpet mechanism and the toroidal pore model [17]. Besides membrane dysfunction and disruption caused by the leakage of ions and metabolites and depolarization of the transmembrane potential, membrane permeabilization is crucial for the translocation of certain AMPs into the cytoplasm acting on key cellular mechanisms such as DNA, RNA and protein synthesis, enzymatic activity, protein folding and cell wall synthesis [14,15].

Since the interaction of the AMP with the membrane is the key step for the mechanism of most AMPs, discrimination between eukaryotic and prokaryotic membrane is crucial for potential successful drug candidate. The difference between bacterial and mammalian membranes enables as selective action of the AMPs. In contrast to bacteria, the cytoplasmic membrane of mammalian cells has neutral net charge consisting of mainly zwitterionic phospholipids such as phosphatidylcholine, sphingomyelin and phosphatidylethanolamine [18,19]. In addition, mammalian cell membranes have a high content of cholesterol which is supposed to reduce the antimicrobial activity of AMPs by stabilizing the membrane [10,18,20]. Despite those fundamental differences between mammalian and bacterial membranes, many AMPs are hemolytic and able to lyse mammalian cells. Minimizing cell toxicity, while at the same time maximizing antimicrobial activity is a major challenge in the development of AMPs for clinical applications.

It must be expected that AMPs during evolution have evolved towards a high general activity against multiple microorganisms. In this study, we address the issues of species specificity, hemolytic activity and antimicrobial activity of Cap18, a α-helical peptide of the cathelicidin family. Our previous study demonstrated that Cap18, originally isolated from rabbit neutrophils, has high antimicrobial activity against a broad range of pathogenic bacteria, is highly thermostable and showed no hemolytic activity *in* vitro [21]. In addition, a recent study evaluated a potential therapeutic effect of Cap 18 against the red mouth disease in juvenile rainbow trout caused by *Y*. *ruckeri* either by oral administration or intraperitoneal injection. It was concluded that, injection of Cap18 into juvenile rainbow trout before exposure to *Y*. *ruckeri* was associated with lower mortality compared to non-treated fish [22]. Based on those properties, Cap18 has the potential to act as lead peptide for further development and optimization. Here, we report the design of a Cap18 peptide library consisting of 696 Cap18 derivatives which was screened for antimicrobial and hemolytic activity and analyzed for species specific killing. In particular, the Cap18 library was screened for antimicrobial activity against *S*. Typhimurium, an important foodborne pathogen and *Y*. *ruckeri* and *A*. *salmonicida*, two important fish pathogens accounting for substantial economic losses in aquaculture. We successfully identified Cap18 variants with changed target specificity and species-specific killing properties and amino acid residues important for antimicrobial and hemolytic activity. Based on our results, we show that changing one single amino acid of Cap18 can lead to changed species specificity of Cap18.

## Materials and methods

### Bacterial strains and growth conditions

The strains used in this study are listed in Table 1. The *Y*. *ruckeri* strain was kindly provided by Prof. Kurt Buchmann, University of Copenhagen, Faculty of Health and Medical Sciences, Denmark.

All strains were grown in Mueller-Hinton-II medium, except *L*. *monocytogenes* which was grown in BHI medium and *L*. *lactis* which was grown in MRS medium. Incubation took place aerobically at 37°C, except for *Y*. *ruckeri* and *A*. *salmonicida*, which were grown aerobically at RT (20 °C), and *L*. *lactis* grown aerobically at 30°C. All plates were incubated for 16–21 hours.

**Table 1:** Strains used in this study.

Strain	Relevant characteristics /genotype	Reference(s)
*Aeromonas salmonicida* ATCC33658	Type strain	ATCC strain collection
*Yersinia ruckeri* 392/2003		[23]
*Salmonella enterica* serovar Typhimurium LT2		sequenced
*Lactococcus lactis* IL1403		[24]
*Escherichia coli* ATCC25922	Clinical isolate, Serotype O6, Biotype 1, reference strain	ATCC strain collection
*Staphylococcus aureus* ATCC29213	reference strain for antimicrobial susceptibility testing	ATCC strain collection
*Enterococcus faecalis* ATCC29212	reference strain for antimicrobial susceptibility testing	ATCC strain collection
*Pseudomonas aeruginosa* ATCC27853	reference strain for antimicrobial susceptibility testing	ATCC strain collection
*Listeria monocytogenes* N22-2	Isolate from fish processing industry	[25]
*Escherichia coli* ATCC25922	Clinical isolate, Serotype O6, Biotype 1, reference strain	ATCC strain collection

### Antimicrobial peptides

All peptides used in this study were purchased as chemically synthesized peptides with either crude purity for the peptide library peptides from Genscript or with high purity from Genscript or Peptide 2.0. The purity of each peptide was determined by the supplier by HPLC and MS analysis. Peptide purity values are given in S1 Table. All peptides were dissolved in 100% DMSO at a stock concentration of 10 mg/ml and stored at -20°C.

### Antimicrobial susceptibility testing (MIC testing)

The minimum inhibitory concentrations (MICs) of the AMPs were measured in 96-well microtiter plates according the Clinical and Laboratory Standards Institute (CLSI, formerly National Committee for Clinical Laboratory Standards [NCCLS]) [26]. Briefly, liquid Mueller-Hinton-II medium containing increasing concentrations of AMPs is inoculated with a defined number of cells (approx. 10^5^ CFUs/ml) in 96-well microtiter plates (polypropylene), whereas each plate also includes a positive growth control and a negative control (sterile control). The range of peptide concentrations analyzed was 0.125–64 μg/ml for the high purity peptides and 0.06–32 μg/ml for peptides of the variant library. After incubation, the MIC is determined by the lowest concentration showing no visible growth. All plates were incubated for 16–20 hours. The MIC of the reference antibiotics was determined by the use of Sensititre panels (Trek Diagnostic Systems Ltd, East Grinstead, UK).

### Cytotoxicity assay

The cytotoxicity for each AMP was determined spectrophotometrically by measuring the haemoglobin release from horse erythrocytes. Briefly, fresh defibrinated horse blood was washed three times with PBS, centrifuged for 15 minutes at 1000g and resuspended at 10% (v/v) in PBS. Samples of the washed horse erythrocytes (100 μl) were transferred to a 96 well microtiter plate and mixed with 100 μl AMP solution. The final AMP concentration in the assay was (32 μg/ml). PBS was used as a negative control, and 0.2% TritonX-100 was used as a positive control. The microtiter plates were incubated for 60 minutes at 37°C and then centrifuged for 10 minutes at 1300g. The supernatants were transferred to a flat-bottom 96 well polystyrene mircotiter plate and the haemoglobin release was monitored by measuring the absorbance at 540 nm. The percentage of haemolysis was calculated as 100 *(A_sample_−A_PBS_)/(ATritonX-100 –A_PBS_), where A_sample_ is the experimental absorbance of the peptide sample, A_PBS_ is the control absorbance of untreated erythrocytes, and A_TritonX-100_ is the absorbance of 0.2% TritonX-100 lysed cells.

## Results

### Broad antimicrobial activity of Cap18

Cap18 is highly active in particular against Gram-negative bacteria including the foodborne pathogens *Salmonella* Typhimurium and *Campylobacter jejuni* and the fish pathogens *Yersina ruckeri* and *Aeromonas salmonicida*, which are a major problem in fish farming (Table 2). The antimicrobial activity of Cap18 against *Y*. *ruckeri* is similar to the well-known antibiotics ampicillin, gentamicin and polymyxin E and even more active than nalidixic acid. However, Cap18 is not only active against pathogenic organisms, but shows a broader range of antimicrobial activity including high antimicrobial activity against beneficial organisms such as *Lactococcus lactis* (Table 2)

**Table 2:** Antimicrobial activity of Cap18 and selected antibiotics.

	Antimicrobial Activity MIC [μg/ml]					
	Cap18—Pure (≥ 89.5% purity)	Cap18—Library (47.5% purity)	Ampicillin	Gentamicin	Nalidixic acid	Polymyxin E
*Yersinia ruckeri* 392/2003	2–4	2	2	1–2	32	1–2
*Aermononas salmonicida* ATCC33658	4	2	≤1	1	≤4	2
*Salmonella* Typhimurium LT2	4–8	2–4	≤1	0.5–1	≤4	2
*Lactococcus lactis* IL1403	1–2	2–4	n.d.	n.d.	n.d.	n.d.

Data are collected as minimal inhibitory concentrations (MICs) according to the Clinical and Laboratory Standards Institute (CLSI) and expressed in μg/ml. All MIC determinations were carried out in triplicates for Cap18—pure and in duplicates for Cap18 –library. MIC determination for the standard antibiotics were carried out in triplicates. n.d = not determined. MIC values are given as a single value when replicates gave identical results whereas two values are given when replicates differed by one well. For standard antibiotics the symbol ≤ is used to indicate sensitivity to the lowest concentration for this antibiotic in the Sensititre plate.

### Design of a Cap18 positional scanning library consisting of 703 peptide variants

In order to optimize the antimicrobial properties of Cap18 and to identify specific residues crucial for the antimicrobial activity, a positional scanning library of Cap18 was designed (S1 Fig). By sequentially substituting each amino acid of the wild-type Cap18 peptide by all other natural amino acids, a peptide library consisting of 703 chemically synthesized peptides was generated. Each individual peptide of this positional scanning library harbors one single amino acid change compared to the Cap18 wild-type peptide. The library peptides were purchased from Genscript as chemically synthesized peptides with crude purity. The purity of the individual peptides varied from 15.8% to 81% purity, whereas most of the peptides showed a purity between 30%-60% (S1 Table). After correcting for the different purity of each peptide, only 7 peptides were not soluble in 100% DMSO, including the peptides with the following amino acid changes: K4F, P33K, P33N, P33S, R34C, T35V and D36F. Those peptides were discarded and therefore not used for further screening. To validate the quality of the peptide library, the MIC values were measured for the high purity Cap18 peptide (≥89.5% purity) and for the library Cap18 peptide (47.5% purity) and compared (Table 2).

### Antimicrobial activity screening of 696 Cap18 derivatives against *Yersinia ruckeri*, *Aeromonas salmonicida* and *Salmonella* Typhimurium

**Fig 1:**
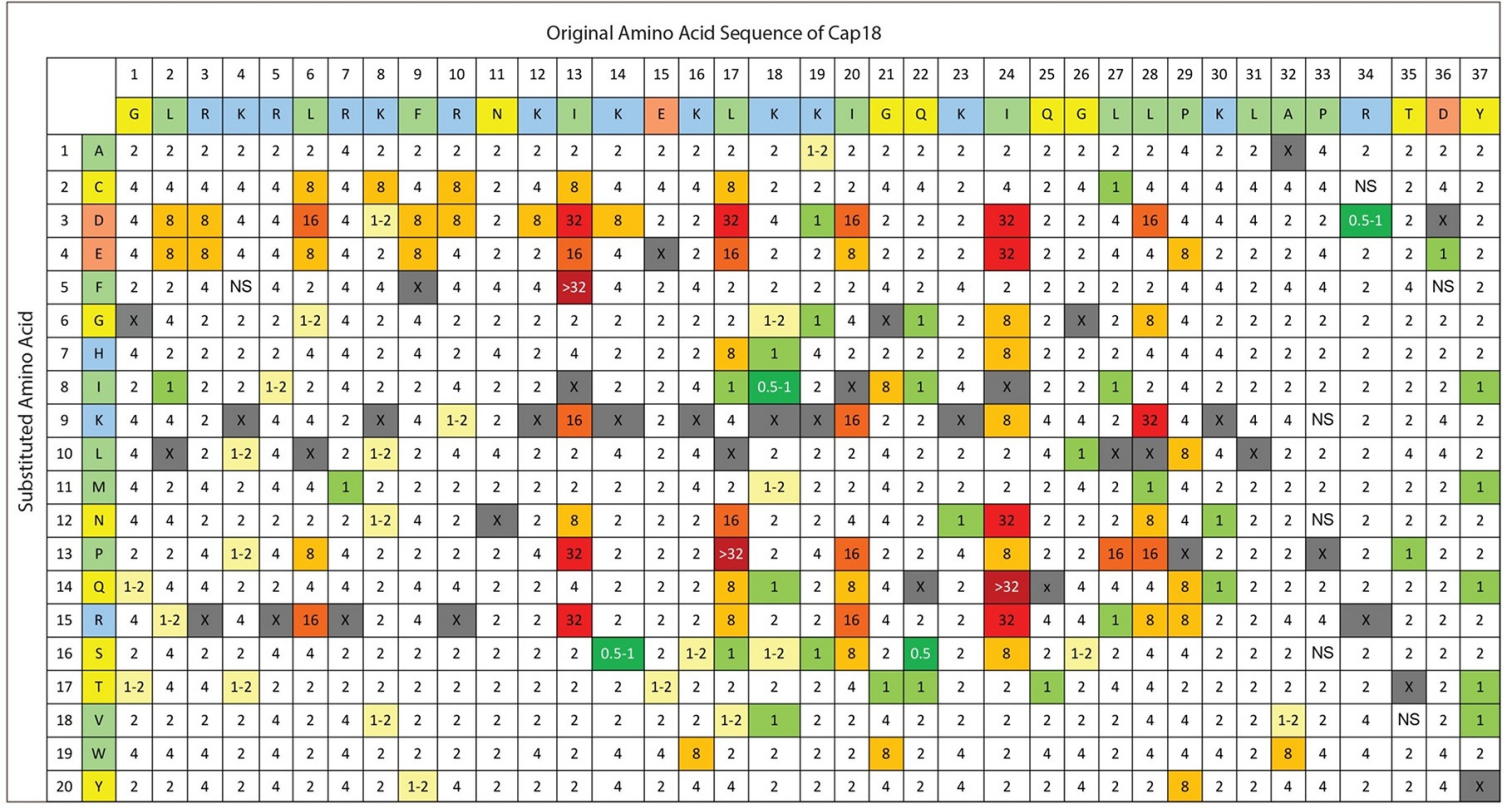
Complete Substitution Analysis of Cap18 measuring the antimicrobial activity against *Aeromonas salmonicida*. The original Cap18 sequence (GLRKRLRKFRNKIKEKLKKIGQKIQGLLPKLAPRTDY) and amino acid assignments are presented in the first two rows. The second column identifies the amino acid substitution at each position (A-Y). Each box in the matrix represents a Cap18 peptide harboring one single amino acid substitution compared to the original Cap18 sequence. For example, the amino acid sequence of the peptide in column 1/row 1 is ALRKRLRKFRNKIKEKLKKIGQKIQGLLPKLAPRTDY, the sequence of the peptide in column 1/row 2 is CLRKRLRKFRNKIKEKLKKIGQKIQGLLPKLAPRTDY, the sequence of peptide in column 2/row 1 is GARKRLRKFRNKIKEKLKKIGQKIQGLLPKLAPRTDY etc.; the values within each box represent a MIC value (μg/ml) against *Aeromonas salmonicida*. Grey boxes represent the original Cap18 sequence. NS = no MIC value determination possible due to the insolubility of the peptide in DMSO.

**Fig 2:**
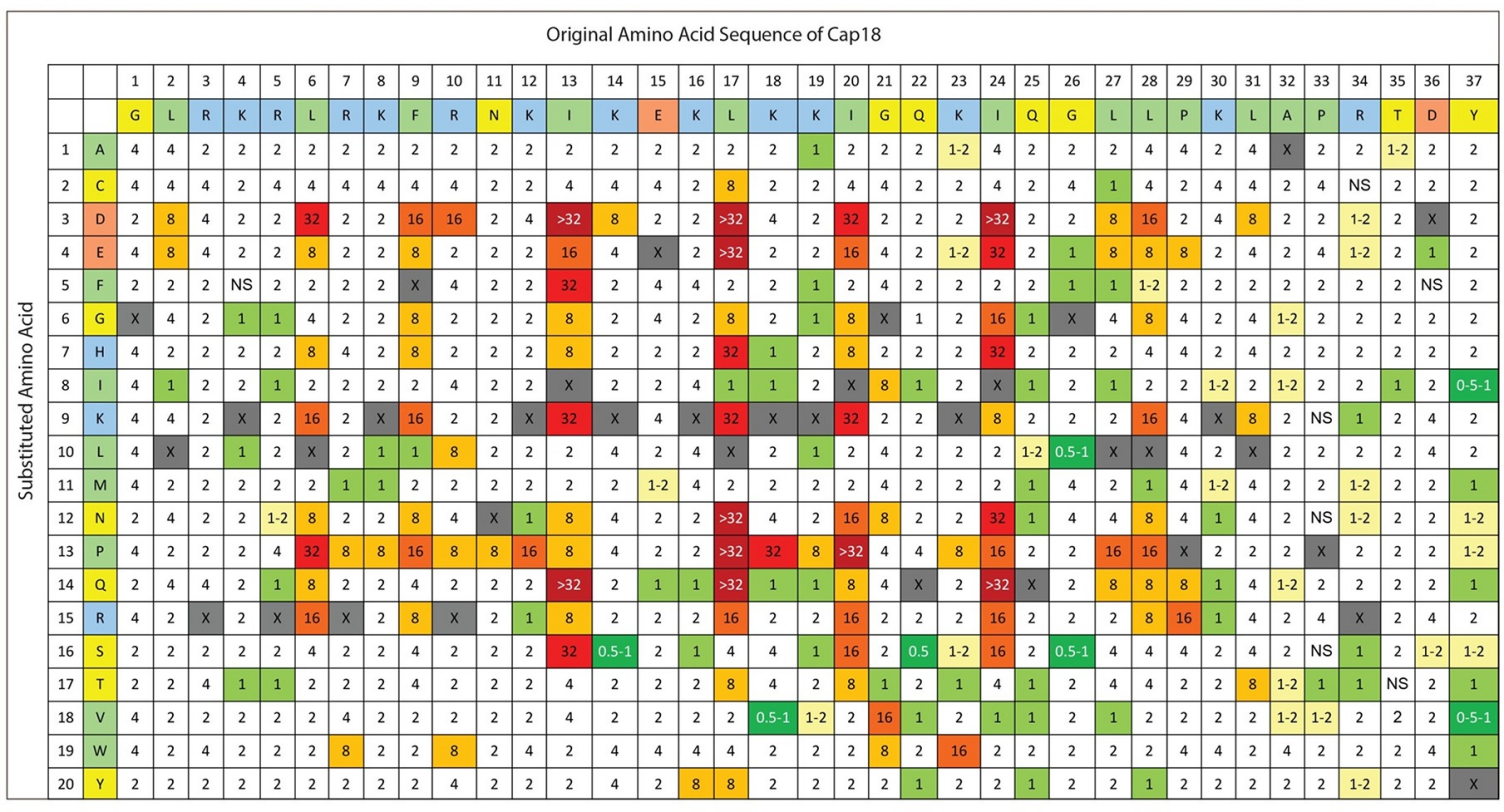
Complete Substitution Analysis of Cap18 measuring the antimicrobial activity against *Yersinia ruckeri*. The original Cap18 sequence (GLRKRLRKFRNKIKEKLKKIGQKIQGLLPKLAPRTDY) and amino acid assignments are presented in the first two rows. The second column identifies the amino acid substitution at each position (A-Y). Each box in the matrix represents a Cap18 peptide harboring one single amino acid substitution compared to the original Cap18 sequence. For example, the amino acid sequence of the peptide in column 1/row 1 is ALRKRLRKFRNKIKEKLKKIGQKIQGLLPKLAPRTDY, the sequence of the peptide in column 1/row 2 is CLRKRLRKFRNKIKEKLKKIGQKIQGLLPKLAPRTDY, the sequence of peptide in column 2/row 1 is GARKRLRKFRNKIKEKLKKIGQKIQGLLPKLAPRTDY etc.; the values within each box represent a MIC value (μg/ml) against *Yersinia ruckeri*. Grey boxes represent the original Cap18 sequence. NS = no MIC value determination possible due to the insolubility of the peptide in DMSO.

**Fig 3:**
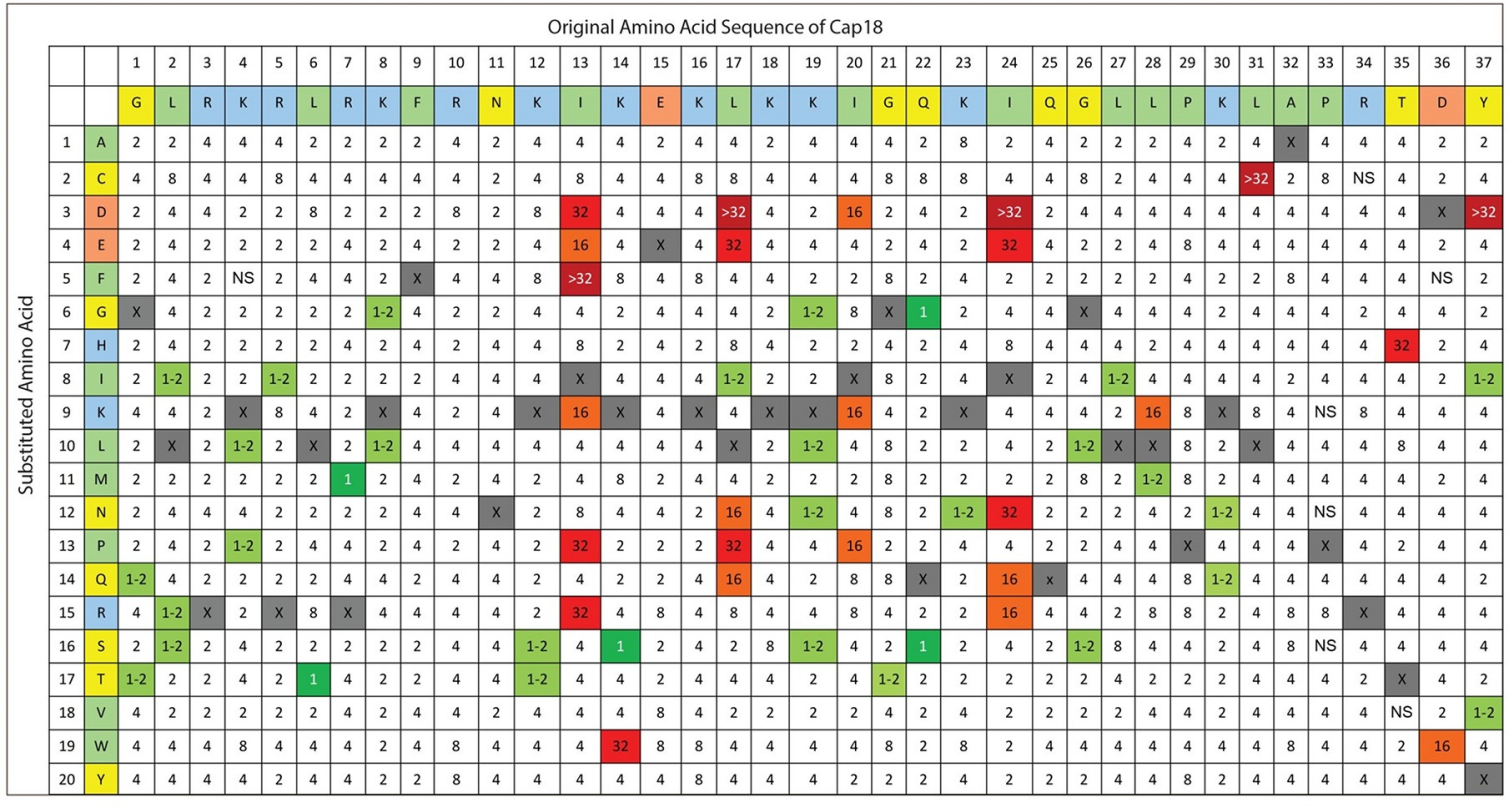
Complete substitution Analysis of Cap18 measuring the antimicrobial activity against *Salmonella* Typhimurium. The original Cap18 sequence (GLRKRLRKFRNKIKEKLKKIGQKIQGLLPKLAPRTDY) and amino acid assignments are presented in the first two rows. The second column identifies the amino acid substitution at each position (A-Y). Each box in the matrix represents a Cap18 peptide harboring one single amino acid substitution compared to the original Cap18 sequence. For example, the amino acid sequence of the peptide in column 1/row 1 is ALRKRLRKFRNKIKEKLKKIGQKIQGLLPKLAPRTDY, the sequence of the peptide in column 1/row 2 is CLRKRLRKFRNKIKEKLKKIGQKIQGLLPKLAPRTDY, the sequence of peptide in column 2/row 1 is GARKRLRKFRNKIKEKLKKIGQKIQGLLPKLAPRTDY etc.; the values within each box represent a MIC value (μg/ml) against *Salmonella* Typhimurium. Grey boxes represent the original Cap18 sequence. NS = no MIC value determination possible due to the insolubility of the peptide in DMSO.

The 696 Cap18 derivatives were screened for antimicrobial activity against *Salmonella* Typhimurium, *Aeromonas salmonicida* and *Yersinia ruckeri*, all important Gram-negative pathogens either in human health or animal production. The antimicrobial activity was recorded for each individual peptide by measuring the MIC values against all three pathogens. The screening results are summarized (Figs 1–3). For *A*. *salmonicida*, 55.2% of all the tested peptides showed the same antimicrobial activity as the original Cap18 (Table 3). 53 derivatives (7.6%) showed a 0.5–2 fold increase and 4 derivatives (0.4%) exhibited a 2–4 fold increase compared to the original Cap18 peptide. A major loss in antimicrobial activity was measured for 25 (3.6%) Cap18 peptides with an 8 fold decrease and 11 (1.6%) variants with a decrease of at least 16 fold compared to the original peptide (Table 3). Similar results were obtained for the screening against *Y*. *ruckeri* (Table 3). For *Y*. *ruckeri*, 53.9% of the derivatives showed unchanged activity; only 7 peptides had a 2–4 fold increased activity while 26 derivatives showed a major reduction of activity of at least 16-fold. For *S*. Typhimurium, 82.7% of all the tested Cap18 derivatives unchanged antimicrobial activity. 5 Cap18 derivatives with a 2–4 fold increased and 14 variants with ≥8-fold reduced antimicrobial activity were identified (Table 3).

**Table 3:** Overview of the antimicrobial activity of 696 Cap18 derivatives.

		MIC [μg/ml]									
		>32	32	16	8	4	2	1–2	1	0.5–1	0.5
*A*. *salmonicida* ATCC33658											
	Number of variants	3	9	13	40	186	388	23	30	3	1
	%	0.4%	1.3%	1.9%	5.7%	26.7%	55.7%	3.3%	4.3%	0.4%	0.1%
Y. ruckeri 392/2003											
	Number of variants	10	13	24	52	127	375	26	62	6	1
	%	1.4%	1.9%	3.4%	7.5%	18.2%	53.9%	3.7%	8.9%	0.9%	0.1%
S. Typhimurium LT2											
	Number of variants	5	9	11	63	342	234	27	5	0	0
	%	0.7%	1.3%	1.6%	9.1%	49.1%	33.6%	3.9%	0.7%	0%	0%
*L*. *lactis* IL1403											
	Number of variants	36	11	42	177	269	145	-	14	-	2
	%	5.2%	1.6%	6.0%	25.4%	38.7%	20.8%	-	2%	-	0.3%

Cap18 variants with unchanged MIC value compared to the original Cap18 are highlighted in grey

### An intact hydrophobic face is crucial for the antimicrobial activity of Cap18

The screening data of 696 Cap18 derivatives allowed identifying key residues of Cap18 which are important for the antimicrobial activity specifically against *A*. *salmonicida*, *Y*. *ruckeri* or *S*. Typhimurium. The analysis of the antimicrobial activity of Cap18 against *A*. *salmonicidia* revealed that the hydrophobic residues I13, L17, I24 and L28 are important for the antimicrobial activity of Cap18. In particular, Cap18 derivatives with amino acids changes at position I13 either by D, F, P or R show a major loss of activity compared to the original Cap18 peptide. The MIC values are more than 16 times higher than the original Cap18 (Table 4). Similarly, substituting amino acid L17 by D or P, amino acid I24 by D, E, N, Q, R and amino acid L28 by K led to a major loss of antimicrobial activity (Table 4).

**Table 4:** Unfavorable amino acid substitutions in Cap18 based on MIC values of Cap18 derivatives for *A*. *salmonicida*, *Y*. *ruckeri*, *L*. *lactis* and *S*. Typhimurium leading to a reduced antimicrobial activity.

Position	Parent Amino Acid	Not Favored Substitution–Reduction of Antimicrobial Activity (MIC ≥ 32 μg/ml)			
		*A*. *salmonicida*	*Y*. *ruckeri*	*S*. Typhimurium	*L*. *lactis*
1	G				
2	L				
3	R				
4	K				
5	R				
6	L		D,P		R
7	R				
8	K				
9	F				
10	R				L
11	N				
12	K				
13	I	D,F,P,R	D,F,K,Q,S	D,F,P	D,E,F,H,K,N,P,Q,R
14	K				
15	E				
16	K				C,F,I,L,M,Y
17	L	D,P	D,E,H,K,N,P,Q	D,E,P	C,D,E,H,K,N,P,Q
18	K		P		P
19	K				
20	I		D,K,P		D,E,H,K,N,P,Q,R,
21	G				C,I,L,
22	Q				
23	K				
24	I	D,E,N,Q,R	D,E,H,N,Q	D,E,N,	C,D,E,G,H,N,Q,R,S
25	Q				
26	G				
27	L				P
28	L	K			
29	P				
30	K				
31	L			C	
32	A				
33	P				
34	R				
35	T			H	
36	D				
37	Y			D	

Similar results were found for the specific activity of Cap18 against *Y*. *ruckeri*. The hydrophobic positions L6, I13, L17, I20, I24 are important for the antimicrobial activity of Cap18. In particular, substituting residue L6 by D or P, residue I13 by D, F, K, Q or S, residue I20 by D, K, P, residue I24 by D, E, H, N or Q led to major loss of antimicrobial activity. In addition, exchanging the charged residue K18 by P reduced the efficacy of Cap18 against *Y*. *ruckeri* by a factor of ≥16 (Table 4).

Against *S*. Typhimurium the antimicrobial activity was abolished by introducing D, F, or P at the hydrophobic position I13; D, E, or P at position L17; or D, E, N at position I24. In addition, the Cap18 peptide harboring a L31C mutation lost the antimicrobial activity with a MIC ≥32. However, not only changing hydrophobic residues of Cap18 will lead to a reduced antimicrobial activity against *S*. Typhimurium. Cap18 T35H and Cap18 Y37D have a MIC MIC ≥32, and are therefore ineffective against *S*. Typhimurium (Table 4). To conclude, these data suggest that an intact hydrophobic interface of Cap18 consisting of the residues I13, L17 and I24 is required for the antimicrobial activity of Cap18 against all the tested pathogens *Y*. *ruckeri*, *A*. *salmonicida* and *S*. Typhimurium.

### Screening for Cap18 derivatives with changed species-specificity

**Fig 4:**
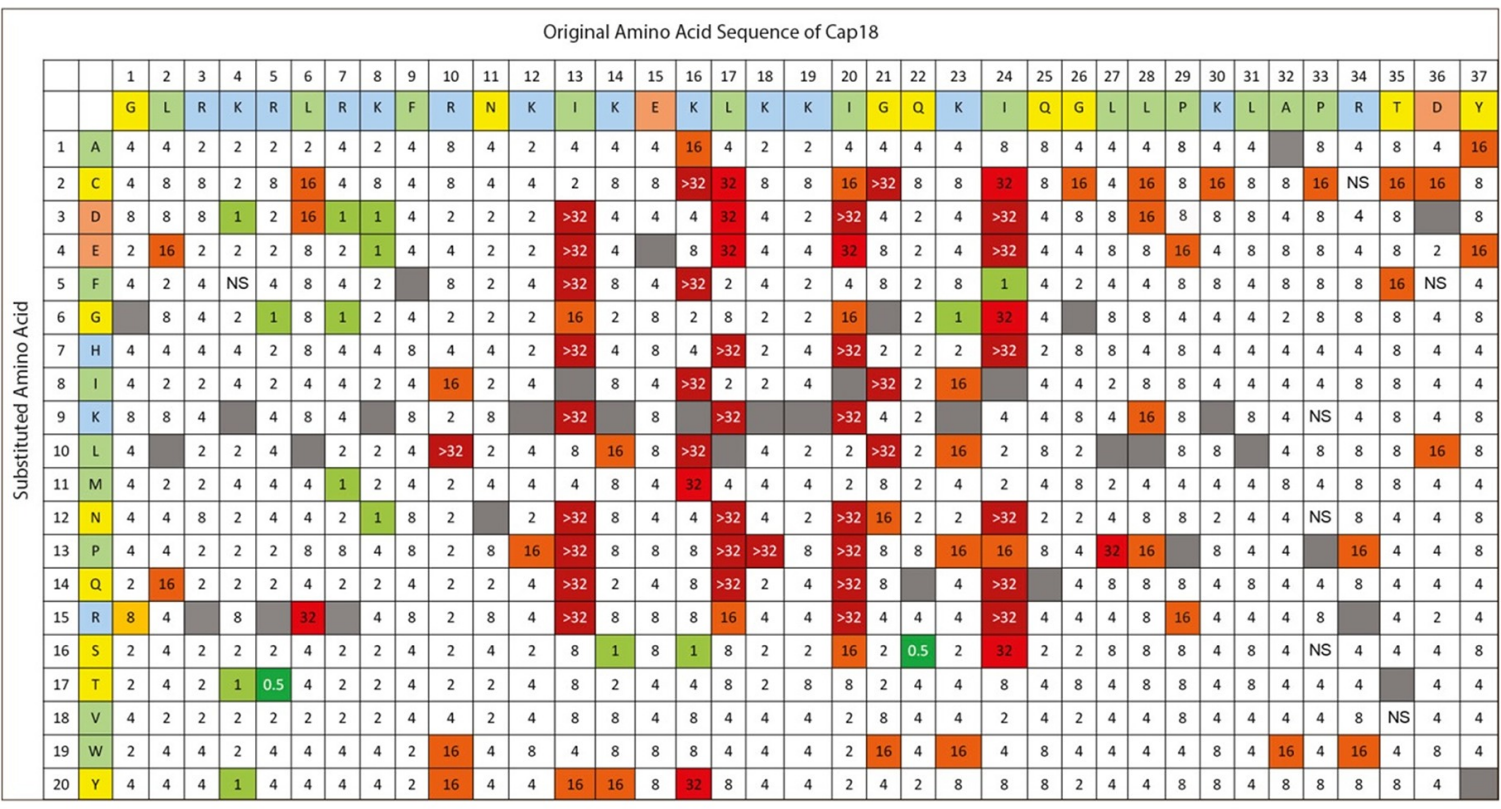
Complete Substitution Analysis of Cap18 measuring the antimicrobial activity against *Lactococcus lactis*. The original Cap sequence is (GLRKRLRKFRNKIKEKLKKIGQKIQGLLPKLAPRTDY) and amino acid assignments are presented in the first two rows. The second column identifies the amino acid substitution at each position (A-Y). Each box in the matrix represents a Cap18 peptide harboring one single amino acid substitution compared to the original Cap18 sequence. For example, the amino acid sequence of the peptide in column 1/row 1 is ALRKRLRKFRNKIKEKLKKIGQKIQGLLPKLAPRTDY, the sequence of the peptide in column 1/row 2 is CLRKRLRKFRNKIKEKLKKIGQKIQGLLPKLAPRTDY, the sequence of peptide in column 2/row 1 is GARKRLRKFRNKIKEKLKKIGQKIQGLLPKLAPRTDY etc.; the values within each box represent a MIC value (μg/ml) against *Lactococcus lactis*. Grey boxes represent the original Cap18 sequence. NS = no MIC value determination possible due to the insolubility of the peptide in DMSO.

The use of traditional antibiotics not only selects for resistance, but also disturbs and alters the microbiota, which plays an important role in human and animal health. Therefore it would be desirable to design AMPs with both, high antimicrobial activity and high species selectivity. To identify and design Cap18 derivatives, which are both, highly active and exhibit a targeted species specificity by only killing one specific target pathogen, the Cap18 library was screened for antimicrobial activity against both, pathogenic and beneficial bacteria. In addition to the screened pathogens *Y*. *ruckeri*, *A*. *salmonicida* and *S*. Typhimurium, the Cap18 peptide library was screened for the loss of antimicrobial activity against the beneficial organism *Lactococcus lactis*. The MIC values representing the antimicrobial activity against *L*. *lactis* are summarized in Fig 4 and Table 3. 59.5% of the Cap18 variant peptides had a MIC value of 2–4 μg/ml which corresponds to wild-type activity. 5.2% of the tested Cap18 derivatives showed a MIC of ≥32 μg/ml which corresponds to loss of antimicrobial activity of more than 8 fold, whereas only 2.3% were slightly more active the original Cap18 peptide (Table 3).

To identify Cap18 variant peptides with changed species specificity, the antimicrobial screening data of *Y*. *ruckeri*, *A*. *salmonicida*, *S*. Typhimurium and *L*. *lactis* was compared. Cap18 peptides with a changed antimicrobial spectrum were identified and classified into two different groups (class I and II) according to the following selection criteria (Table 5): Class I peptides have lost the antimicrobial activity against all the screened test strains (MIC ≥32 μg/ml) and class II peptides show a species specific loss of antimicrobial activity (targeted antimicrobial activity). More detailed, peptides from class II exhibit wild-type antimicrobial activity (Table 2) against at least one test strain while at the same time having completely lost the antimicrobial activity against at least another screening strain (MIC ≥32 μg/ml). To summarize, out of 696 tested Cap18 derivatives, 34 peptides showed a changed species specificity (Class II peptides). Those peptides kept unchanged antimicrobial activity against at least one screening strain, while at the same time being ineffective against at least another screening strain. Interestingly, only 7 derivatives (class I) lost the antimicrobial activity completely and had no antimicrobial effect against all the tested organisms.

**Table 5:** Cap18 derivatives identified in the screening with lost antimicrobial activity or changed species-specificity.

Cap18 variant peptides				Antimicrobial Activity–MIC in μg/ml			
Amino Acid	Position	Peptide Class	Mutation	*L*. *lactis*	*S*. Typhimurium	*Y*. *ruckeri*	*A*. *salmonicida*
L	6	II	L6D	16	8	≥32	16
		II	L6P*	8	4	≥32	8
		II	L6R	≥32	8	16	16
R	10	II	R10L*	≥32	4	8	4
I	13	I	I13D*	≥32	≥32	≥32	≥32
		I	I13F*	≥32	≥32	≥32	≥32
		II	I13H*	≥32	8	8	4
		II	I13N	≥32	8	-	8
		II	I13Q*	≥32	4	≥32	4
		II	I13S*	8	4	≥32	2
K	16	II	K16C*	≥32	8	2	4
		II	K16F*	≥32	8	4	4
		II	K16I*	≥32	4	4	4
		II	K16L*	≥32	4	4	4
		II	K16M*	≥32	4	4	4
		II	K16Y*	≥32	8	8	4
L	17	II	L17C	≥32	8	?	8
		I	L17D*	≥32	≥32	≥32	≥32
		II	L17H	≥32	8	≥32	8
		II	L17K*	≥32	4	≥32	4
		I	L17P*	≥32	≥32	≥32	≥32
K	18	II	K18P*	≥32	4	≥32	2
I	20	II	I20E*	≥32	4	16	8
		II	I20H*	≥32	2	8	2
		II	I20N*	≥32	4	16	4
		II	I20Q	≥32	8	8	8
		II	I20R	≥32	8	16	16
G	21	II	G21C*	≥32	8	4	4
		II	G21I	≥32	8	8	8
		II	G21L*	≥32	8	4	4
I	24	II	I24C*	≥32	4	4	4
		I	I24D*	≥32	≥32	≥32	≥32
		I	I24E	≥32	≥32	≥32	≥32
		II	I24G*	≥32	4	16	8
		II	I24H	≥32	8	≥32	8
		I	I24N*	≥32	≥32	≥32	≥32
		II	I24S*	≥32	4	16	8
L	27	II	L27P*	≥32	4	16	16
L	31	II	L31C	8	≥32	4	4
T	35	II	T35H	8	≥32	2	2
Y	37	II	Y37D	8	≥32	2	2

Class I peptides are highlighted in light grey and class II peptides are shown in dark grey. Cap18 variant peptides chosen for further analysis using pure peptides (purity >95%) are marked with *.

### Generating Cap18 peptides with engineered species specificity by introducing one single amino acid exchange

**Fig 5:**
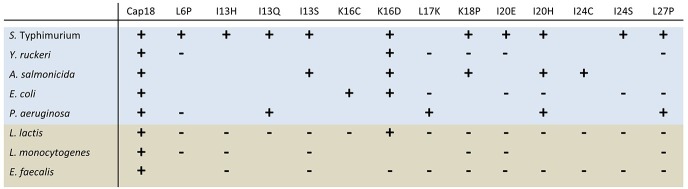
Cap18 derivatives with changed species-specificity. Unchanged antimicrobial activity compared to the original Cap18 is indicated by +, whereas the loss of antimicrobial activity (MIC ≥32 μg/ml) is illustrated by -. Gram-negative bacteria are highlighted in light blue, Gram-positive bacteria are presented in light brown.

To validate and confirm the screening results, 6 peptides from class I and 22 Cap18 peptides from class II were ordered as highly pure peptides (purity >95%). In addition, three Cap18 derivatives harboring either a I13F, I13M or G26T substitution were ordered as positive controls. These three derivatives showed unchanged antimicrobial activity compared to the original Cap18 peptide in the initial screening (S1 Table). The antimicrobial activity of the highly pure peptides was determined by measuring the MIC value of each derivative against the four test strains *Y*. *ruckeri*, *A*. *salmonicida*, *S*.Typhimurium and *L*. *lactis* (Table 6). By comparing the MIC data, Cap18 derivatives from class II with changed species-specificity could be identified. Cap18 derivatives with a changed antimicrobial profile being ineffective against at least one tested organism (MIC ≥32 μg/ml), while at the same time retained full antimicrobial activity against another species exhibit species-specific antimicrobial activity. All Cap18 derivatives with changed species-specificity and their antimicrobial activity pattern are summarized in Fig 5. In addition to the additional screening strains, a wider range of relevant pathogens including Gram-positive and Gram-negative bacteria was analyzed for targeted antimicrobial activity. The MIC values were determined using highly pure peptides against *E*. *coli*, *P*. *aeruginosa*, *L*. *monocytogenes* and *E*. *faecalis* and the results are summarized in Table 6.

**Table 6:** Antimicrobial activity of purified Cap18 derivatives against Gram-negative and Gram-positive bacteria.

		Antimicrobial Activity [μg/ml]							
		Gram-negative bacteria					Gram-positive bacteria		
Cap18 peptides	Substitution	*S*. Typhimurium LT2	*Y*. *ruckeri* 392/2003	*A*. *salmonicida* ATCC33658	*E*. *coli* ATCC25922	*P*. *aeruginosa* ATCC27853	*L*. *lactis* IL1403	*L*. *monocytogenes* N22-2	*E*. *faecalis* ATCC29212
Cap18—pure	Original sequence	4–8	2–4	4	4–8	4–8	1–2	4	8
Peptide **1**	L6P	8	64	8–16	8	≥64	≥64	32	32
Peptide **2**	R10L	16	32	32	32	32	≥64	8–16	16–32
Peptide **3**	I13D	≥64	≥64	≥64	≥64	≥64	≥64	32–64	≥64
Peptide **4**	I13F	4	2	4	4–8	4–8	2	4	8–16
Peptide **5**	I13H	4	8–16	2–4	4	8–16	≥64	32	≥64
Peptide **6**	I13M	8	4	4	8–16	8–16	2	4	8–16
Peptide **7**	I13Q	4–8	8	8	16–32	8	≥64	16	32
Peptide **8**	I13S	8	8–16	4	16	8–16	≥64	32	≥64
Peptide **9**	K16C	8–16	8–16	8	8	16–32	≥64	16	32
Peptide **10**	K16D	4	4	2–4	4–8	16–32	2	4–8	32
Peptide **11**	K16F	16	16–32	16–32	16	32	≥64	16	16
Peptide **12**	K16I	16	16	8–16	16	16	≥64	8–16	16
Peptide **13**	K16L	16	16	16	16	16–32	≥64	8–16	16
Peptide **14**	K16M	16	8–16	8–16	16	16–32	≥64	16	32
Peptide **15**	K16Y	16	8–16	16	16	16	≥64	8–16	16
Peptide **16**	L17D	≥64	≥64	≥64	≥64	≥64	≥64	≥64	≥64
Peptide **17**	L17K	16	≥64	16–32	≥64	8	≥64	16–32	≥64
Peptide **18**	L17P	≥64	≥64	≥64	≥64	≥64	≥64	≥64	≥64
Peptide **19**	K18P	4	32	2–4	8–16	16	32	32–64	≥64
Peptide **20**	I20E	8	32	16	≥64	16	32	32	≥64
Peptide **21**	I20H	8	16	4	32	8	32	16	≥64
Peptide **22**	I20N	8–16	≥64	16	≥64	8–16	≥64	16–32	≥64
Peptide **23**	G21C	16	16	16–32	16	32	≥64	16	32
Peptide **24**	G21L	16	16	16	16	16	≥64	16	16
Peptide **25**	I24C	8–16	16	4–8	16	16	≥64	16	64
Peptide **26**	I24D	≥64	≥64	≥64	≥64	≥64	≥64	≥64	≥64
Peptide **27**	I24G	8–16	32	8	≥64	16	≥64	32	≥64
Peptide **28**	I24N	32	≥64	≥64	≥64	64/16	≥64	32	≥64
Peptide **29**	I24S	8	16	8	32–64	8–16	32	16–32	≥64
Peptide **30**	G26T	4–8	2–4	4	4–8	8	2	4	8
Peptide **31**	L27P	8	32	16	≥64	8	32	32	≥64

Data are collected as minimal inhibitory concentrations (MICs) according to the Clinical and Laboratory Standards Institute (CLSI) and expressed in μg/ml. All MIC determinations were carried out in triplicate. MIC values are given as a single value when replicates gave identical results whereas two values are given when replicates differed by one well.

Based on all the collected MIC data using highly pure Cap18 peptides, we can conclude that 13 derivatives of Cap18 were identified with changed species-specificity by introducing one single amino acid substitution compared to the original Cap18 peptide (Fig 5). In addition, introducing the following substitutions I13D, L17D, L17P, I24D or I24N leads to a complete loss in antimicrobial activity (MIC values ≥ 32 μg/ml) against all the tested bacterial species including Gram-positive and Gram-negative bacteria. Amino acids I13, L17, and I24 of Cap18 seem to be of specific importance for general antimicrobial activity of Cap18 against both, Gram-negative and Gram-positive bacteria. These findings are in agreement with the initial screening data (Table 4).

### The proline residue at position 29 is essential for the differentiation between prokaryotic and eukaryotic cells

Not only a high antimicrobial activity, but also the ability to differentiate between bacterial and mammalian cells is an important characteristic for a successful antimicrobial peptide. The cytotoxicity was determined using a hemolytic assay based on the lysis of washed horse erythrocytes. The Cap18 peptide with high purity (≥89.5%) had at the peptide concentration of 64 μg/ml a very minimal hemolytic activity (1% compared to the triton X-100 control) (Table 7) which is in agreement with previously published data [21]. To validate the original Cap18 peptide from the positional scanning library, which has a lower purity of only 47.5% purity, the hemolytic activity was determined to 2% ± 1% (32 μg/ml final peptide concentration in the assays), which was very similar to the measured hemolytic activity of the pure Cap18 peptide (Table 7). Summarizing, both the highly pure Cap18 peptide and the library Cap18 peptide showed minimal hemolytic activity at the measured concentrations. The solvent DMSO alone had no hemolytic activity in the concentration range used in the assay (data not shown).

**Table 7:** Unfavorable amino acid substitutions of Cap18 leading to increased hemolytic activity against horse erythrocytes.

Position	Parent Amino Acid	Hemolytic activity		
		6% -10%	11%-15%	≥16%
1	G			
2	L		I	
3	R			
4	K			
5	R	W		
6	L			
7	R			
8	K			
9	F			
10	R	F,L,W		
11	N			
12	K	W		
13	I			
14	K	W		
15	E			
16	K	A,C,F,I,L,M,V,W	S	
17	L			I
18	K	A,H	Q,Y	
19	K	G		
20	I			
21	G	F,L,Y	W	
22	Q	K		S
23	K	A,I,N,V,W,Y	C,F,L,M	
24	I			
25	Q	C,I,L,V,W,Y		F
26	G	E,W	L,R	
27	L	C,I		
28	L	F,H	M	
29	P	E,F,H,I,K,L,M,N,R,V,W	C	D
30	K	C,Q,W	Y	
31	L			
32	A	F,I	C	
33	P			W
34	R	K,R		
35	T	I		C
36	D	C,W,Y	F	
37	Y	I,M,Q		

**Fig 6:**
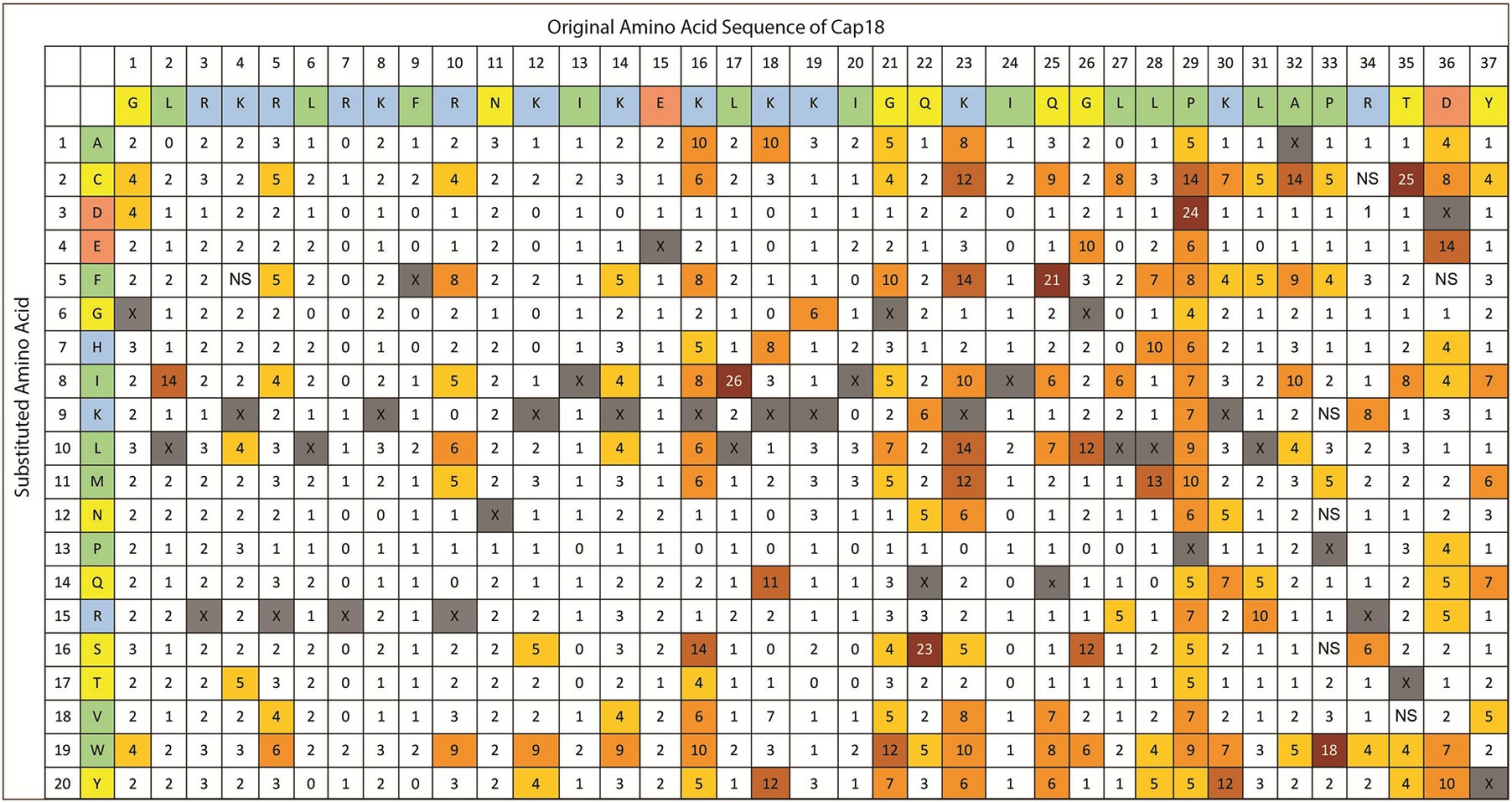
Complete Substitution Analysis of Cap18 measuring the hemolytic activity of Cap18 peptides against horse erythrocytes. The original Cap18 sequence (GLRKRLRKFRNKIKEKLKKIGQKIQGLLPKLAPRTDY) and amino acid assignments are presented in the first two rows. The second column identifies the amino acid substitution at each position (A-Y). Each box in the matrix represents a Cap18 peptide harboring one single amino acid substitution compared to the original Cap18 sequence. For example, the amino acid sequence of the peptide in column 1/row 1 is ALRKRLRKFRNKIKEKLKKIGQKIQGLLPKLAPRTDY, the sequence of the peptide in column 1/row 2 is CLRKRLRKFRNKIKEKLKKIGQKIQGLLPKLAPRTDY, the sequence of peptide in column 2/row 1 is GARKRLRKFRNKIKEKLKKIGQKIQGLLPKLAPRTDY etc.; the values within each box represent the hemolytic activity measured relative to full lysis by 0.2% Trition X-100. The final peptide concentration in the assay is 32 μg/ml. Grey boxes with X represent the original Cap18 sequence. NS = no MIC value determination possible due to the insolubility of the peptide in DMSO.

To identify which residues of Cap18 play a central role in generating specificity between eukaryotic and prokaryotic cells, the hemolytic activity of all the 696 Cap18 derivatives was therefore determined measuring the hemoglobin release of horse erythrocytes at a peptide concentration of 32 μg/ml (Fig 5). Out of the 696 tested Cap18 derivatives, 550 peptides (79.1%) had no or minimal hemolytic activity similar to the original Cap18 peptide. 60 Cap18 derivatives showed an hemolytic activity of 4–5% which is a slight increase compared to the original Cap18 peptide. 64 derivatives displayed a hemolytic activity of 6–10% compared to the original Cap18 peptide, 16 derivatives had a hemolytic activity of 11–15% and 6 derivatives exhibited a hemolytic activity of ≥16% (Fig 6, Table 8). Exchanging proline 29 by any other amino acid led to increased hemolytic activity compared to the non-hemolytic Cap18 original peptide. Substituting residues K16, G21 and K23 by any hydrophobic amino acid (A, F, I, L, M V or W) raises the hemolytic activity of the derivatives compared to the original Cap18 peptide. In addition, substituting negatively charged residues at position R5, R10, K12, K4, K16, K23, K30 and R34 leads to higher hemolytic activity (Fig 6, Table 8). To validate the screening data, the hemolytic activity of the pure Cap18 derivatives Peptide **1–31** which were previously selected based on changed antimicrobial activity pattern (Table 6), was determined. In addition, five Cap18 derivatives harboring a substitution at P29 were ordered as peptides with high purity (Peptide **32**–**36**) and the hemolytic activity was determined. Results of the hemolytic assay are presented in Table 7. In summary, the screening data and the dataset using purified peptides provide evidence that residue P29, K16, G21 and L23 plays the central role in the hemolytic activity of Cap18 and are involved in generating specificity between eukaryotic and prokaryotic cells.

**Table 8:** Hemolytic activity against horse erythrocytes, calculated hydrophobicity, hydrophobic moment and net charge of Cap18 and Cap18 derivatives.

Peptide	Substitution	Peptide concentration [μg/ml]	Haemolytic Activity [%]	Hydrophobicity <H>	Hydrophobic Moment <μH>	Net Charge
Cap18 –pure^1)^	original	64	1% ± 1%	0.107	0.471	+12
Cap18 –library^2)^	original	32	2% ± 1%	0.107	0.471	+12
Peptide **1**	L6P	64	1%	0.080	0.445	+12
Peptide **2**	R10L	64	6%	0.180	0.518	+11
Peptide **3**	I13D	64	1%	0.037	0.402	+11
Peptide **4**	I13F	64	2%	0.106	0.471	+12
Peptide **5**	I13H	64	1%	0.062	0.426	+12
Peptide **6**	I13M	64	2%	0.091	0.456	+12
Peptide **7**	I13Q	64	1%	0.052	0.417	+12
Peptide **8**	I13S	64	1%	0.057	0.421	+12
Peptide **9**	K16C	64	4%	0.175	0.502	+11
Peptide **10**	K16D	64	1%	0.113	0.473	+10
Peptide **11**	K16F	64	9%	0.182	0.505	+11
Peptide **12**	K16I	64	10%	0.182	0.505	+11
Peptide **13**	K16L	64	14%	0.179	0.504	+11
Peptide **14**	K16M	64	8%	0.167	0.497	+11
Peptide **15**	K16Y	64	9%	0.159	0.494	+11
Peptide **16**	L17D	64	0%	0.040	0.417	+11
Peptide **17**	L17K	64	1%	0.034	0.412	+13
Peptide **18**	L17P	64	0%	0.080	0.449	+12
Peptide **19**	K18P	64	1%	0.153	0.441	+11
Peptide **20**	I20E	64	1%	0.041	0.413	+11
Peptide **21**	I20H	64	1%	0.062	0.431	+12
Peptide **22**	I20N	64	0%	0.042	0.414	+12
Peptide **23**	G21C	64	4%	0.148	0.485	+12
Peptide **24**	G21L	64	7%	0.153	0.486	+12
Peptide **25**	I24C	64	2%	0.100	0.464	+12
Peptide **26**	I24D	64	0%	0.037	0.403	+11
Peptide **27**	I24G	64	1%	0.058	0.424	+12
Peptide **28**	I24N	64	1%	0.042	0.408	+12
Peptide **29**	I24S	64	1%	0.057	0.423	+12
Peptide **30**	G26T	64	2%	0.114	0.465	+12
Peptide **31**	L27P	64	1%	0.080	0.453	+12
Peptide **32**	P29A	64	4%	0.096	0.481	+12
Peptide **33**	P29D	64	3%	0.066	0.507	+11
Peptide **34**	P29F	64	7%	0.136	0.445	+12
Peptide **35**	P29H	64	3%	0.091	0.485	+12
Peptide **36**	P29S	64	4%	0.086	0.489	+12

^1)^ Purity ≥89.5%

^2)^ Purity 47.5%

The hemolytic activity is measured in duplicates and given as the average ± SD in % relative to full lysis by 0.2% triton X-100. Mean hydrophobicity <H>, hydrophobic moment <μH>, and net charge are calculated using heliquest (http://heliquest.ipmc.cnrs.fr).

## Discussion

The number of antimicrobials entering the market is significantly decreasing since the golden age of the antibiotic discovery in the mid-1900s and simultaneously, the levels of antimicrobial resistance amongst bacterial pathogens are rising which calls for alternative solutions controlling bacterial infections [27]. The majority of the currently used antimicrobials have a broad killing spectrum. This has the advantage that they can be used without precise diagnosis, but they also disrupt the normal microbiota by killing the beneficial commensal bacteria which can lead to severe side-effects. Antimicrobial peptides are an attractive alternative to create novel, target-specific antimicrobials. The aim of this study was to design and identify antimicrobial peptides with greater specificity and more targeted antimicrobial activity based on the cathelicidin Cap18, a cationic AMP originally isolated from rabbit neutrophils.

Combinatorial libraries, prepared through chemical synthesis, or biological libraries such as phage display, represent powerful tools to optimize existing antimicrobial peptides [28]. By designing and screening a positional scanning library of Cap18, we successfully designed Cap18 peptide derivatives with changed species specificity and the potential to be used in target-specific antimicrobial therapy by introducing single amino acid substitutions in the original Cap18 sequence. Generally, by screening the positional scanning library of Cap18, we were able to analyze the antimicrobial and hemolytic properties of Cap18 and to identify specific residues within the amino acid sequence of Cap18 which are crucial for antimicrobial activity and required for the inherent low hemolytic activity of Cap18. Peptide libraries are a powerful tool to optimize the properties of antimicrobial peptides. However, the quality and purity of the peptides is important for the subsequent screening applications and the accuracy of the obtained results. Since we used a peptide library consisting of crude purity peptides, positive hits identified in the screening needed further confirmation and verification using purified peptides (purity ≥95%). Comparing the MIC values recorded for crude and highly pure peptides, we can suggest that the quality of a crude peptide library allows the discrimination between active and non-active Cap18 derivatives. More subtle changes in the antimicrobial activity are difficult to measure using crude peptides due to impurities even after adjusting for the different purities of the individual Cap18 derivatives. The impurities in the crude peptides of low yield are likely to be peptides of related sequences with premature termination or other mistakes in the synthesis. Such peptides might have similar antimicrobial activity to the peptide of desired sequence and if considered as a mere diluent the antimicrobial activity might be overestimated. Crude peptide libraries will therefore be more efficient in detecting loss of function than in detecting modest increase in activity In order to be able to measure smaller changes in the antimicrobial activity, peptide libraries with high purity are needed. This might only be applicable and a valid alternative for shorter peptides due to the high synthesis costs.

**Fig 7:**
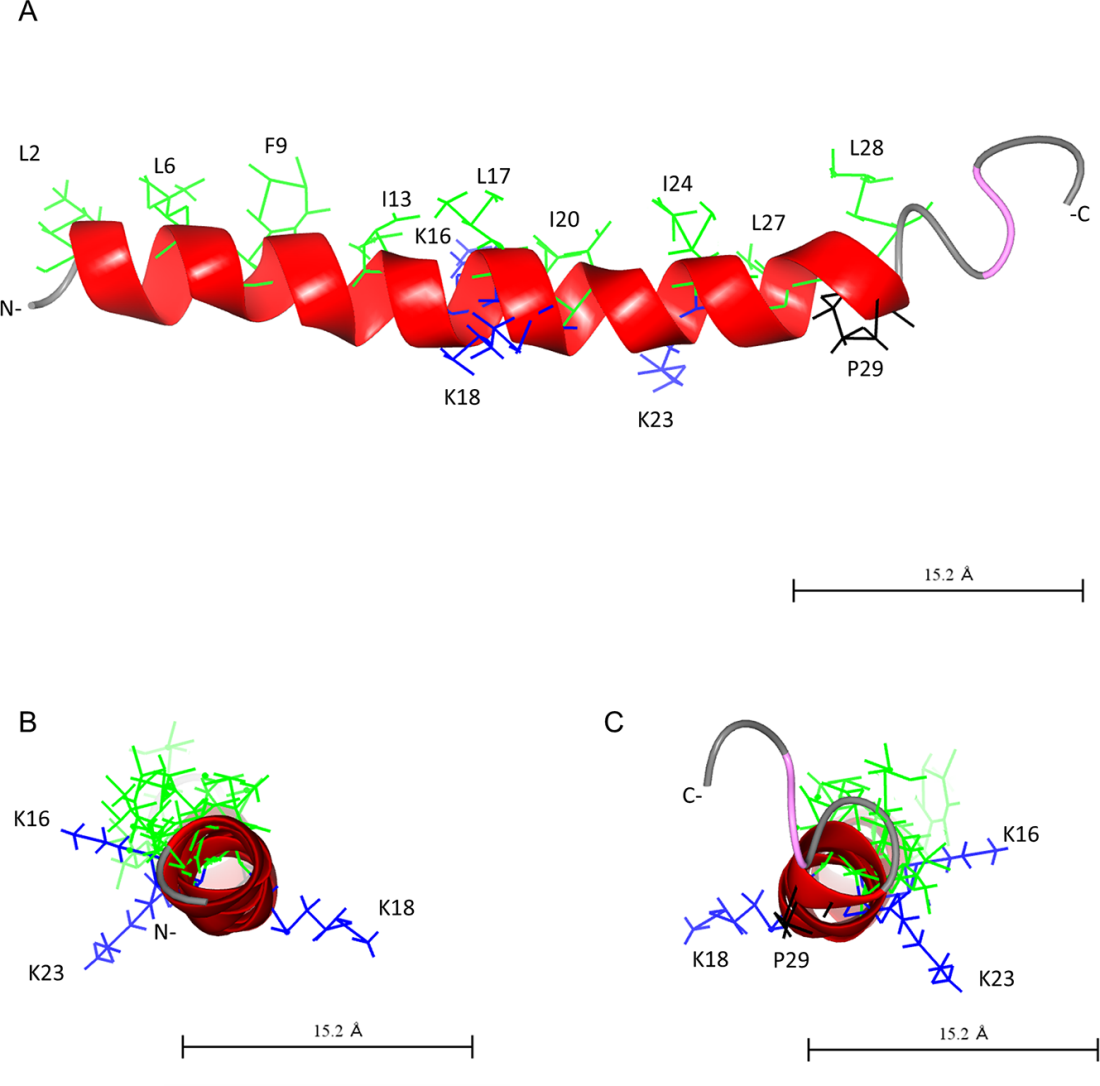
Predicted structure of Cap18. The structure of Cap18 was predicted using I-Tasser [30] and visualized by CCP4 software [31]. The predicted α-helix is highlighted in red. Hydrophobic residues of the α-helix are shown in green. Specific residues are highlighted the following: K16, K18 and K23 in blue and P29 in black. A: view along helix axis B: view from N- to C-terminal C: view form C- to N-terminal.

**Fig 8:**
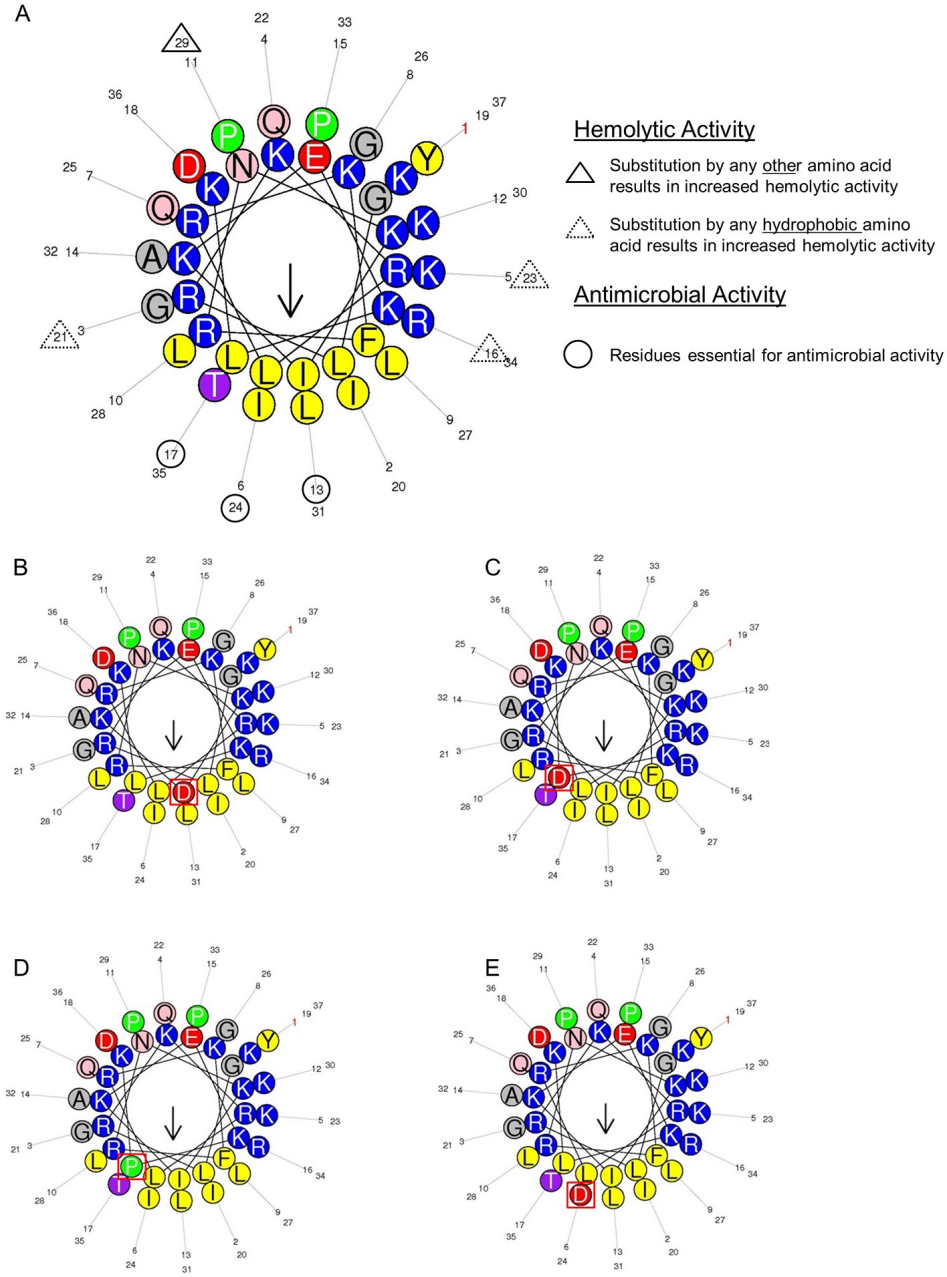
Helical wheel projection of the original Cap18 peptide and Cap18 derivatives. Hydrophobic amino acids are yellow, negatively charged amino acids are red and positively charged amino acids are in dark blue. Particular polar residues are violet (threonine, serine) or pink (asparagine, glutamine). Glycine and alanine are grey and proline residues are shown in green. The helices were created using http://heliquest.ipmc.cnrs.fr/ [32]. A: helical wheel projection of the original Cap18 peptide. Residues important for the hemolytic activity and antimicrobial activity of Cap18 are highlighted. B-E: helical wheel projections of Cap18 derivatives that lost the antimicrobial activity against all the tested organisms. Peptide 3 harboring the I13D amino acid substitution (B), Peptide 16 harboring the L17D substitution (C), Peptide 18 harboring the L17P substitution (D) and Peptide 26 harboring the I24D substitution (E). Corresponding substitutions are highlighted.

Physiochemical and structural parameters such as hydrophobicity, cationicity, amphipathicity and amino acid composition are important determinants for the antimicrobial activity of α-helical AMPs (reviewed [13]). By screening the positional scanning library of Cap18, followed by the validation of potential hits using peptides with high purity, specific residues within the Cap18 amino acid sequence were identified which are strictly required for antimicrobial activity against Gram-positive as well as Gram-negative bacteria. Peptides **3, 16, 18, 26** and **28** harboring either a I13D, L17D, L17P, I24D or I24N substitution lost their antimicrobial activity and are completely ineffective against all tested bacterial species. Residues I13, L17 and I24 are all part of the hydrophobic face highlighted in the predicted structure or illustrated by the helical wheel projection of Cap18 (Figs 7 and 8). The introduction of non-hydrophobic amino acids in particular the negatively charged residue D at position 13, 17 or 24 will disrupt the hydrophobic interface (Fig 8). The disruption of the hydrophobic patch will most likely prevent optimal interaction with the bacterial membrane and therefore interfere with the insertion of the peptide in to the lipid bilayer. This reduced antimicrobial efficacy of peptides **3, 16, 18, 26** and **28** nicely correlates with a reduction in the mean hydrophobicity <H> and the mean hydrophobic moment <μH> calculated by HeliQuest (http://heliquest.ipmc.cnrs.fr). The mean hydrophobic moment <μH> is a measure of the amphiphilicity of the α-helix, in which the length and direction of the <μH> vector are depending on the hydrophobicity and the position of the side chains along the helix axis. Large <μH> value implies that the helix is amphipathic perpendicular to its axis [29]. Apart from peptides **3, 16, 18, 26** and **28**, additional derivatives with substitutions at positions 13, 17 or 24 displayed a changed antimicrobial activity pattern. In contrast to peptides **3, 16, 18, 26** and **28,** peptides **5, 7** and **8** retained full antimicrobial activity against some bacterial species and at the same time became ineffective against other bacteria. Those variants are acting in a species-specific manner compared to the original Cap18 peptide having a broad antimicrobial activity. In more detail, Peptide **5** harboring a I13H substitution retained full antimicrobial activity against *S*. Typhimurium LT2, reduced activity against the other Gram-negative bacteria tested and completely lost efficacy against the three tested Gram-positive species. Similarly, peptides **7** and **8** showed changed species-specificity. Peptide **7** (I13Q) was completely ineffective against *L*. *lactis*, however fully active against *S*. Typhimurium LT2 and *P*. *aeruginosa* ATCC27853, whereas peptide **8** (I13S) was active against *S*. Typhimurium LT2 and *A*. *salmonicida* ATCC33658 and ineffective against all three Gram-positive species tested. Even though the values of the calculated hydrophobicity and hydrophobic moment are lower for peptides **5, 7** and **8** compared to the original Cap18 peptides, peptides **5, 7** and **8** retained full antimicrobial activity against some selected species. In addition, selected substitutions at the hydrophobic residues L6, I20 and L27, all part of the hydrophobic face highlighted in predicted structure and illustrated by the helical wheel projection, exhibit a changed antimicrobial activity spectrum (Fig 5). The calculated hydrophobicity and hydrophobic moment values of those derivatives, peptide **2** (L6P), peptide **20** (I20E), peptide **21** (I20H) and peptide **31** (I27P), are lowered compared to the original Cap18 peptide. Summarizing, we suggest that the hydrophobic residues in the hydrophobic face might be promising targets to change the antimicrobial activity spectrum of Cap18 and to generate Cap18 derivatives which are killing in species-specific manner. However, our data also suggest that not only residues in the hydrophobic face might be targets for the generation of species-specificity. Peptides **9** and **10** have cysteine or aspartic acid substitution at position K16, which is part of the hydrophilic side of Cap18, highlighted in the structure and helical wheel projection (Figs 7 and 8). In contrast to the derivatives having substitutions in the hydrophobic face, peptides **9** (K16C) and **10** (K16D) are more hydrophobic than the original Cap18 peptide displaying an increased hydrophobicity and hydrophobic moment. Based on our results, we argue that there exists an optimal hydrophobicity of Cap18 depending on the target organisms. A threshold hydrophobicity at which optimal antimicrobial activity can be obtained is highly dependent on the target organism. Fine-tuning of the hydrophobicity and hydrophobic moment of Cap18 might be a promising possibility to adjust the antimicrobial activity dependent on the target organism and to generate Cap18 derivatives with a very narrow killing spectrum.

However, hydrophobicity and amphipathicity are not the only parameters determining the antimicrobial activity of the individual Cap18 derivatives. Even though e.g. peptide **18** (L17P) is less hydrophobic than peptide **17** (L17K) based on the calculations of <H> and <μH>, peptide **18** has completely lost its antimicrobial activity being ineffective against all the tested strains, whereas peptide **17** retained full antimicrobial activity against *P*. *aeruginosa*. A reduced hydrophobicity or hydrophobic moment value alone are not sufficient to abolish the antimicrobial activity of Cap18 arguing that the amino acid composition plays an important role. Proline is a well-known helix-breaker causing a kink in the helix. This is caused by proline being unable to complete the H-bonding chain and by steric effects preventing proline from adapting the preferred helical geometry [33]. Glycine has poor helix forming properties and tends to disrupt α-helices due to its high conformational flexibility. Therefore, the presence of glycine and proline could have a critical effect on the antimicrobial and hemolytic activity of α-helical AMPs. Proline residues are often found within the amphipathic a-helix of AMPs and therefore the importance of proline residues in AMPs has been investigated. Previous studies investigating the effect of proline on the biological activity of α-helical AMPs revealed that substitution of proline decreased the antimicrobial activity. Substitution of the central proline by alanine in gaegurin [34] or by leucine or alanine in the hybrid analog P18 resulted in a reduction of antimicrobial activity [35]. Further, it was shown that the proline hinge in buforin II is responsible for its cell-penetrating ability [36] and the central PXXP hinge of PMAP-23 is important for its antimicrobial activity [37]. Our data on Cap18 shows that the introduction of proline at specific sites of Cap18 changes the antimicrobial activity as well as the species-specificity depending on the site of introduction. Replacing the central leucine at position 17 by a proline causes a complete loss of antimicrobial activity (peptide **18**). This finding is in agreement with previous studies which showed that the introduction of proline in α-helical AMPs decreased the ability of the peptide to penetrate the cytoplasmic membrane of *E*. *coli* which was concomitant with a reduction in the antimicrobial activity [38,39]. However, introducing a proline residue at position L6, K18 or L27 does not abolish antimicrobial activity completely, but generates Cap18 derivatives with a changed antimicrobial activity spectrum. The antimicrobial activity pattern of peptide **1,** peptide **19** and peptide **31**, all harboring a proline insertion, are different depending on the target species as well as on the insertion site of the proline residue. All three derivatives kept unchanged antimicrobial activity against *S*. Typhimurium LT2, whereas all three were completely ineffective against *Y*. *ruckeri*, *L*. *lactis* and *L*. *monocytogenes*. Analyzing the remaining results of those three derivatives harboring a proline insertion, we can argue that the antimicrobial activity depends on both the position of proline insertion and the target organism itself (Table 5). Summarizing, we can suggest that the dramatic effect of introducing a proline at residue L17 resulting in complete loss of antimicrobial activity might be due to a structural change in the helix preventing optimal interaction with the membrane which is crucial for antimicrobial activity of Cap18. However, the positioning of proline residues determines how the antimicrobial activity is affected–positively or negatively depending on the target organism. This is in agreement with a previous study demonstrating that the properties conferred on AMPs by proline residues strongly depends on the properties of the proline-free template peptide as well as the positioning of the proline residue in the primary sequence [40].

Apart from high antimicrobial activity, low cytotoxicity is a very desirable characteristic for antimicrobial peptides used as potential drug candidates. The original Cap18 peptide displays low hemolytic activity *in vitro* measuring the hemoglobin release of horse and trout erythrocytes [22][21]. In order to design AMPs with optimized characteristics for potential therapeutic use, it is crucial to understand how cell selectivity is generated and which residues are responsible for the inherent low hemolytic activity of Cap18. Analysis of the screening data investigating the hemolytic effect of Cap18 derivatives reveals a crucial role of proline at position 29 for the differentiation between mammalian and bacterial membrane. Substituting P29 by any other amino acid will lead to increased hemolytic activity concluding that the presence of P29 in the original Cap18 is crucial for its inherent low hemolytic activity. Looking at the predicted structure of Cap18, P29 is situated at the very end of the predicted a-helix. The presence of proline residues in AMPs which might cause kinks in the α-helix, not only affects the antimicrobial activity, but also the hemolytic activity. Previous studies have found that the elimination of proline at position 7 in the α-helical pardaxin, substituting proline at position 14 in melittin by alanine or replacing the glycine residues at position 13 or 17 in pleurocidin are resulting in increased hemolytic activity[41–43]. Similarly, incorporating a proline kink in the α-helical AMP Anal 3 by replacing glutamic acid by proline in the middle of the peptide sequence had positive effect on hemolytic activity showing reduced membrane damaging activity of Anal 3-Pro compared to the original Anal 3 peptide [44]. Introducing a proline residue at any other position of Cap18 has no negative effect on the hemolytic activity. Interestingly, the substitution of P29 had no or only very minor effect on the antimicrobial activity regardless of the target organism. This suggests that P29 is crucial for the specificity between prokaryotes and eukaryotes and plays a minor role for the antimicrobial activity of Cap18. Hydrophobicity (H), hydrophobic moment (μH) and net charge are important physiochemical parameters controlling cell selectivity. The initial screening shows that the substitution of the positive charged residues K16 and K23 as well as G21 by any hydrophobic residues (not proline) enhance the hemolytic activity (Fig 6, Table 7). K16, G21, and K23 all being part of the hydrophilic interface are situated right next to the hydrophobic face which illustrated by the helical wheel projection (Fig 8). Peptide **23** and peptide **24** harboring a G21C or G21L substitution are more hydrophobic and more hemolytic active compared to the original Cap18 peptide. Similarly, replacing K16 with hydrophobic amino acids (peptide **9,** peptide **11,** peptide **12,** peptide **13,** peptide **14** and peptide **15**) leads to a higher mean hydrophobicity and hydrophobic moment than the original Cap18 peptide which is consistent with enhanced hemolytic activity (Fig 8). Concluding, we can suggest that enhanced hemolytic activity is correlated with increased hydrophobicity which is in agreement with previous studies [45,46]. In addition, position K16, G21, K23 and P29 play a crucial role in generating selectivity between mammalian and bacterial cells. In particular, the absence of P29 negatively affects the hemolytic activity, there is no or minor effects on the antimicrobial activity and therefore crucial for the selectivity. In contrast, position K16 is not only important for the selectivity between bacterial and mammalian cells, but also for the selectivity between different bacterial species. Peptide **9** (K16C) not only exhibit increased hemolytic activity, but also a changed antimicrobial activity pattern (Fig 5). In contrast, Peptide **10** displays a changed antimicrobial activity pattern, whereas the hemolytic activity is unchanged compared to the original Cap18 peptide. This argues that the substitution K16D only contributes to the specificity between different bacterial strains, but not to a more general specificity distinguishing mammalian and bacterial cells in general.

Based on a very thorough dissection of Cap18 analyzing 696 derivatives of Cap18, we can conclude that specific single amino acid substitution of Cap18 can alter the antimicrobial activity pattern. We were able to generate Cap18 derivatives with target-specific activity. So far, only Cap18 derivatives harboring single amino acid substitutions were analyzed, however, it would be highly interesting to see if further improvement can be achieved by combining single substitutions and to generate derivatives with double or triple substitutions. By screening further organisms and careful analysis of the results, as well determining the structure of selected derivatives investigating the mode of action, it might be possible to predict Cap18 derivatives with targeted activity against any organism. However, it might be challenging to hit the optimal window which is a delicate balance between maximum antimicrobial activity and minimum toxicity to the host cells.

## Supporting information

S1 Fig Positional scanning library of Cap18. The original Cap18 sequence is (GLRKRLRKFRNKIKEKLKKIGQKIQGLLPKLAPRTDY) is presented in the first row. The second column identifies the amino acid substitution at each position (A-Y). Each box in the matrix represents a Cap18 derivative harboring one single amino acid substitution compared to the original Cap18 sequence. For example, the amino acid sequence of the peptide in column 1/row 1 is ALRKRLRKFRNKIKEKLKKIGQKIQGLLPKLAPRTDY, the sequence of the peptide in column 1/row 2 is CLRKRLRKFRNKIKEKLKKIGQKIQGLLPKLAPRTDY, the sequence of peptide in column 2/row 1 is GARKRLRKFRNKIKEKLKKIGQKIQGLLPKLAPRTDY.
(TIF)

S1 Table Antimicrobial peptides used in this study. (DOCX)

## References

[1] French GL. The continuing crisis in antibiotic resistance. Int J Antimicrob Agents. 2010;36 Suppl 3: S3–7. doi: 10.1016/S0924-8579(10)70003-0 10.1016/S0924-8579(10)70003-0 21129629

[2] Goff DA. Antimicrobial stewardship: bridging the gap between quality care and cost. Curr Opin Infect Dis. 2011;24 Suppl 1: S11–20. doi: 10.1097/01.qco.0000393484.17894.05 21200180 10.1097/01.qco.0000393484.17894.05

[3] Gould IM. Coping with antibiotic resistance: the impending crisis. Int J Antimicrob Agents. 2010;36 Suppl 3: S1–2. doi: 10.1016/S0924-8579(10)00497-8 10.1016/S0924-8579(10)00497-8 21129626

[4] Tamma PD, Cosgrove SE. Antimicrobial stewardship. Infect Dis Clin North Am. 2011;25: 245–260. doi: 10.1016/j.idc.2010.11.011 21316003 10.1016/j.idc.2010.11.011

[5] Guarner F, Malagelada J-R. Gut flora in health and disease. Lancet. 2003;361: 512–519. doi: 10.1016/S0140-6736(03)12489-0 12583961 10.1016/S0140-6736(03)12489-0

[6] Kau AL, Ahern PP, Griffin NW, Goodman AL, Gordon JI. Human nutrition, the gut microbiome and the immune system. Nature. 2011;474: 327–336. doi: 10.1038/nature10213 21677749 10.1038/nature10213 PMC3298082

[7] Rashid MU, Weintraub A, Nord CE. Effect of new antimicrobial agents on the ecological balance of human microflora. Anaerobe. 2012;18: 249–253. doi: 10.1016/j.anaerobe.2011.11.005 22155131 10.1016/j.anaerobe.2011.11.005

[8] Buffie CG, Pamer EG. Microbiota-mediated colonization resistance against intestinal pathogens. Nat Rev Immunol. 2013;13: 790–801. doi: 10.1038/nri3535 24096337 10.1038/nri3535 PMC4194195

[9] Wren SM, Ahmed N, Jamal A, Safadi BY. Preoperative oral antibiotics in colorectal surgery increase the rate of Clostridium difficile colitis. Arch Surg. 2005;140: 752–756. doi: 10.1001/archsurg.140.8.752 16103284 10.1001/archsurg.140.8.752

[10] Zasloff M. Antimicrobial peptides of multicellular organisms. Nature. 2002;415: 389–95. doi: 10.1038/415389a 11807545 10.1038/415389a

[11] Maróti G, Kereszt A, Kondorosi É, Mergaert P. Natural roles of antimicrobial peptides in microbes, plants and animals. Res Microbiol. 2011;162: 363–374. doi: 10.1016/j.resmic.2011.02.005 21320593 10.1016/j.resmic.2011.02.005

[12] Hancock REW, Sahl H-G. Antimicrobial and host-defense peptides as new anti-infective therapeutic strategies. Nat Biotech. 2006;24: 1551–1557. doi: 10.1038/nbt1267 17160061 10.1038/nbt1267

[13] Takahashi D, Shukla SK, Prakash O, Zhang G. Structural determinants of host defense peptides for antimicrobial activity and target cell selectivity. Biochimie. 2010 pp. 1236–1241. doi: 10.1016/j.biochi.2010.02.023 20188791 10.1016/j.biochi.2010.02.023

[14] Nguyen LT, Haney EF, Vogel HJ. The expanding scope of antimicrobial peptide structures and their modes of action. Trends in Biotechnology. 2011 pp. 464–472. doi: 10.1016/j.tibtech.2011.05.001 21680034 10.1016/j.tibtech.2011.05.001

[15] Brogden KA. Antimicrobial peptides: pore formers or metabolic inhibitors in bacteria? Nat Rev Microbiol. 2005;3: 238–250. doi: 10.1038/nrmicro1098 15703760 10.1038/nrmicro1098

[16] Mahlapuu M, Håkansson J, Ringstad L, Björn C. Antimicrobial Peptides: An Emerging Category of Therapeutic Agents. Front Cell Infect Microbiol. 2016;6: 194 doi: 10.3389/fcimb.2016.00194 28083516 10.3389/fcimb.2016.00194 PMC5186781

[17] Melo MN, Ferre R, Castanho MARB. Antimicrobial peptides: linking partition, activity and high membrane-bound concentrations. Nat Rev Microbiol. 2009;7: 245–250. doi: 10.1038/nrmicro2095 19219054 10.1038/nrmicro2095

[18] Yeaman MR. Mechanisms of Antimicrobial Peptide Action and Resistance. Pharmacol Rev. 2003;55: 27–55. doi: 10.1124/pr.55.1.2 12615953 10.1124/pr.55.1.2

[19] Ebenhan T, Gheysens O, Kruger HG, Zeevaart JR, Sathekge MM. Antimicrobial Peptides: Their Role as Infection-Selective Tracers for Molecular Imaging. Biomed Res Int. 2014;2014: 1–15. doi: 10.1155/2014/867381 25243191 10.1155/2014/867381 PMC4163393

[20] Lai Y, Gallo RL. AMPed up immunity: how antimicrobial peptides have multiple roles in immune defense. Trends in Immunology. 2009 pp. 131–141. doi: 10.1016/j.it.2008.12.003 19217824 10.1016/j.it.2008.12.003 PMC2765035

[21] Ebbensgaard A, Mordhorst H, Overgaard MT, Nielsen CG, Aarestrup FM, Hansen EB. Comparative Evaluation of the Antimicrobial Activity of Different Antimicrobial Peptides against a Range of Pathogenic Bacteria. Mergaert P, editor. PLoS One. Public Library of Science; 2015;10: e0144611 doi: 10.1371/journal.pone.0144611 26656394 10.1371/journal.pone.0144611 PMC4684357

[22] Chettri JK, Mehrdana F, Hansen EB, Ebbensgaard A, Overgaard MT, Lauritsen AH, et al Antimicrobial peptide CAP18 and its effect on *Yersinia ruckeri* infections in rainbow trout *Oncorhynchus mykiss* (Walbaum): comparing administration by injection and oral routes. J Fish Dis. 2017;40: 97–104. doi: 10.1111/jfd.12497 27334068 10.1111/jfd.12497

[23] Fouz B, Zarza C, Amaro C. First description of non-motile Yersinia ruckeri serovar I strains causing disease in rainbow trout, Oncorhynchus mykiss (Walbaum), cultured in Spain. J Fish Dis. 2006;29: 339–346. doi: 10.1111/j.1365-2761.2006.00723.x 16768714 10.1111/j.1365-2761.2006.00723.x

[24] Bolotin A, Wincker P, Mauger S, Jaillon O, Malarme K, Weissenbach J, et al The complete genome sequence of the lactic acid bacterium Lactococcus lactis ssp. lactis IL1403. Genome Res. 2001;11: 731–53. doi: 10.1101/gr.169701 11337471 10.1101/gr.169701 PMC311110

[25] Wulff G, Gram L, Ahrens P, Vogel BF. One group of genetically similar Listeria monocytogenes strains frequently dominates and persists in several fish slaughter- and smokehouses. Appl Environ Microbiol. 2006;72: 4313–4322. doi: 10.1128/AEM.02288-05 16751546 10.1128/AEM.02288-05 PMC1489582

[26] Wikler MA, Cockerill FR, Bush K, Dudley MN, Eliopoulos GM, Hardy DJ, et al Methods for dilution antimicrobial susceptibility tests for bacteria that grow aerobically; approved standard—eighth edition Clinical and Laboratory Standards Institute 2009.

[27] Boucher HW, Talbot GH, Bradley JS, Edwards JE, Gilbert D, Rice LB, et al Bad bugs, no drugs: no ESKAPE! An update from the Infectious Diseases Society of America. Clin Infect Dis. 2009;48: 1–12. doi: 10.1086/595011 19035777 10.1086/595011

[28] Ashby M, Petkova A, Gani J, Mikut R, Hilpert K. Use of Peptide Libraries for Identification and Optimization of Novel Antimicrobial Peptides. Curr Top Med Chem. 2016;17: 537–553. doi: 10.2174/1568026616666160713125555 10.2174/1568026616666160713125555 27411326

[29] Eisenberg D, Weiss RM, Terwilliger TC. The helical hydrophobic moment: a measure of the amphiphilicity of a helix. Nature. 1982;299: 371–374. doi: 10.1038/299371a0 7110359 10.1038/299371a0

[30] Roy A, Kucukural A, Zhang Y. I-TASSER: a unified platform for automated protein structure and function prediction. Nat Protoc. 2010;5: 725–738. doi: 10.1038/nprot.2010.5 20360767 10.1038/nprot.2010.5 PMC2849174

[31] McNicholas S, Potterton E, Wilson KS, Noble MEM. Presenting your structures: The CCP4mg molecular-graphics software. Acta Crystallogr Sect D Biol Crystallogr. 2011;67: 386–394. doi: 10.1107/S0907444911007281 21460457 10.1107/S0907444911007281 PMC3069754

[32] Gautier R, Douguet D, Antonny B, Drin G. HELIQUEST: a web server to screen sequences with specific α-helical properties. Bioinformatics. 2008;24: 2101–2102. doi: 10.1093/bioinformatics/btn392 18662927 10.1093/bioinformatics/btn392

[33] MacArthur MW, Thornton JM. Influence of proline residues on protein conformation. J Mol Biol. 1991;218: 397–412. doi: 10.1016/0022-2836(91)90721-H 2010917 10.1016/0022-2836(91)90721-h

[34] Suh JY, Lee YT, Park CB, Lee KH, Kim SC, Choi BS. Structural and functional implications of a proline residue in the antimicrobial peptide gaegurin. Eur J Biochem. 1999;266: 665–674. doi: 10.1046/j.1432-1327.1999.00917.x 10561611 10.1046/j.1432-1327.1999.00917.x

[35] Shin S y., Yang S-T, Park E j., Eom S h., Song W k., Kim J i., et al Antibacterial, antitumor and hemolytic activities of α-helical antibiotic peptide, P18 and its analogs. J Pept Res. 2001;58: 504–514. doi: 10.1034/j.1399-3011.2001.00934.x 12005420 10.1034/j.1399-3011.2001.00934.x

[36] Park CB, Yi KS, Matsuzaki K, Kim MS, Kim SC. Structure-activity analysis of buforin II, a histone H2A-derived antimicrobial peptide: the proline hinge is responsible for the cell-penetrating ability of buforin II. Pnas. 2000;97: 8245–50. doi: 10.1073/pnas.150518097 10890923 10.1073/pnas.150518097 PMC26932

[37] Yang ST, Jeon JH, Kim Y, Shin SY, Hahm KS, Kim JI. Possible role of a PXXP central hinge in the antibacterial activity and membrane interaction of PMAP-23, a member of cathelicidin family. Biochemistry. 2006;45: 1775–1784. doi: 10.1021/bi051524k 16460024 10.1021/bi051524k

[38] Zhang L, Benz R, Hancock REW. Influence of proline residues on the antibacterial and synergistic activities of α-helical peptides. Biochemistry. 1999;38: 8102–8111. doi: 10.1021/bi9904104 10387056 10.1021/bi9904104

[39] Andreu D, Merrifield RB, Steiner H, Boman HG. N-Terminal analogs of cecropin A: synthesis, antibacterial activity, and conformational properties. Biochemistry. 1985;24: 1683–1688. doi: 10.1021/bi00328a017 3924096 10.1021/bi00328a017

[40] Vermeer LS, Lan Y, Abbate V, Ruh E, Bui TT, Wilkinson LJ, et al Conformational Flexibility Determines Selectivity and Antibacterial, Antiplasmodial, and Anticancer Potency of Cationic α-Helical Peptides. J Biol Chem. 2012;287: 34120–34133. doi: 10.1074/jbc.M112.359067 22869378 10.1074/jbc.M112.359067 PMC3464521

[41] Thennarasu S, Nagaraj R. Specific antimicrobial and hemolytic activities of 18-residue peptides derived from the amino terminal region of the toxin pardaxin. "Protein Eng Des Sel. 1996;9: 1219–1224. doi: 10.1093/protein/9.12.1219 10.1093/protein/9.12.1219 9010936

[42] Dempsey CE, Bazzo R, Harvey TS, Syperek I, Boheim G, Campbell ID. Contribution of proline-14 to the structure and actions of melittin. FEBS Lett. 1991;281: 240–244. doi: 10.1016/0014-5793(91)80402-O 2015901 10.1016/0014-5793(91)80402-o

[43] Lim SS, Song YM, Jang MH, Kim Y, Hahm K-S, Shin SY. Effects of two glycine residues in positions 13 and 17 of pleurocidin on structure and bacterial cell selectivity. Protein Pept Lett. 2004;11: 35–40. doi: 10.2174/0929866043478383 14965277 10.2174/0929866043478383

[44] Lee JK, Gopal R, Park S-C, Ko HS, Kim Y, Hahm K-S, et al A Proline-Hinge Alters the Characteristics of the Amphipathic α-helical AMPs. Delprato AM, editor. PLoS One. 2013;8: e67597 doi: 10.1371/journal.pone.0067597 23935838 10.1371/journal.pone.0067597 PMC3720801

[45] Huang Y, He L, Li G, Zhai N, Jiang H, Chen Y. Role of helicity of α-helical antimicrobial peptides to improve specificity. Protein Cell. 2014;5: 631–642. doi: 10.1007/s13238-014-0061-0 24805306 10.1007/s13238-014-0061-0 PMC4130925

[46] Chen Y, Guarnieri MT, Vasil AI, Vasil ML, Mant CT, Hodges RS. Role of peptide hydrophobicity in the mechanism of action of alpha-helical antimicrobial peptides. Antimicrob Agents Chemother. 2007;51: 1398–1406. doi: 10.1128/AAC.00925-06 17158938 10.1128/AAC.00925-06 PMC1855469

