# Supplementary figures and images for: Dissection of the antimicrobial and hemolytic activity of Cap18: Generation of Cap18 derivatives with enhanced specificity
Source: PLoS One. 2018 May 31;13(5):e0197742. doi: 10.1371/journal.pone.0197742 (PMC5978884; doi:10.1371/journal.pone.0197742)

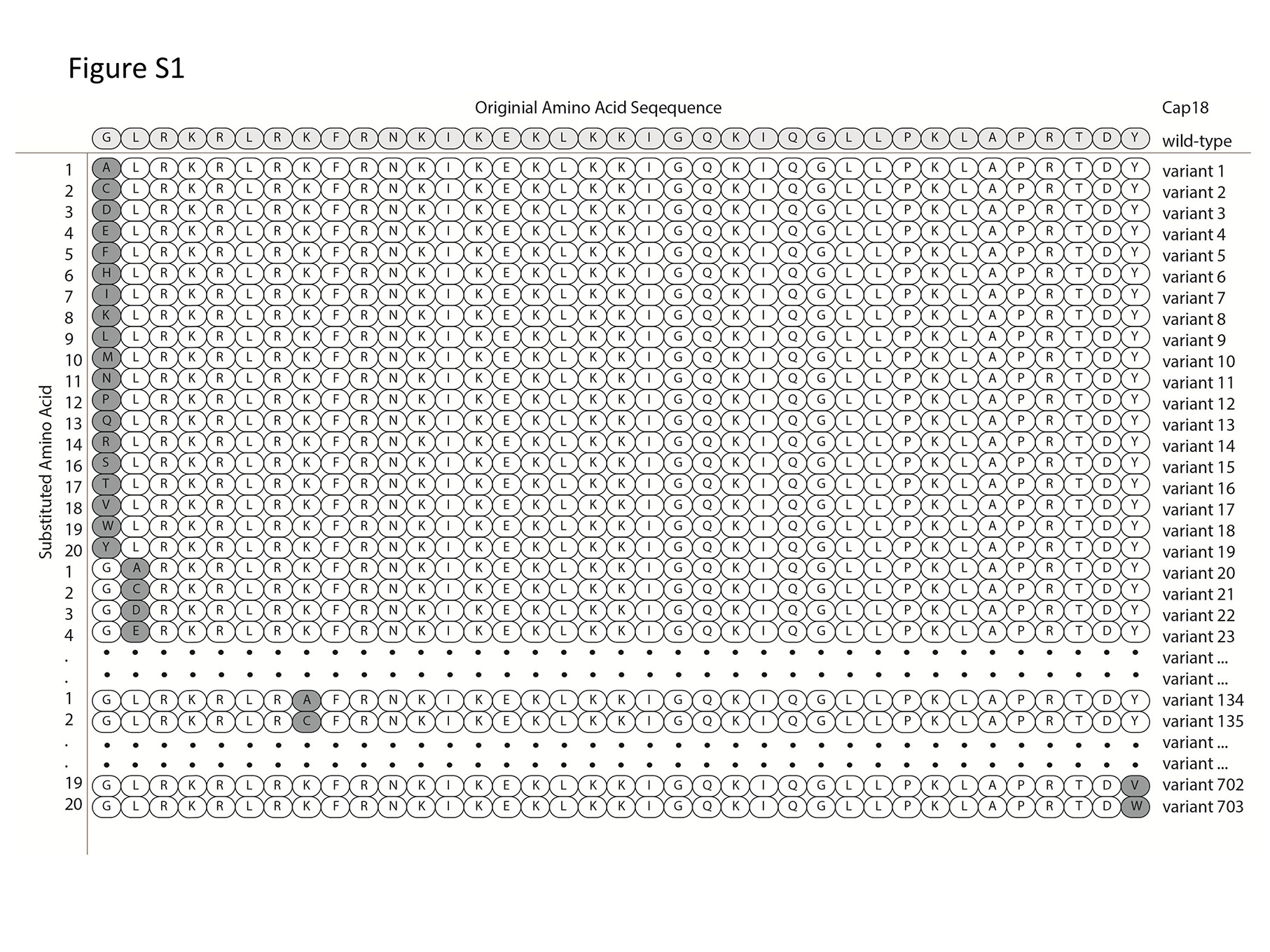

Supplement: S1 Fig — The original Cap18 sequence is (GLRKRLRKFRNKIKEKLKKIGQKIQGLLPKLAPRTDY) is presented in the first row. The second column identifies the amino acid substitution at each position (A-Y). Each box in the matrix represents a Cap18 derivative harboring one single amino acid substitution compared to the original Cap18 sequence. For example, the amino acid sequence of the peptide in column 1/row 1 is ALRKRLRKFRNKIKEKLKKIGQKIQGLLPKLAPRTDY, the sequence of the peptide in column 1/row 2 is CLRKRLRKFRNKIKEKLKKIGQKIQGLLPKLAPRTDY, the sequence of peptide in column 2/row 1 is GARKRLRKFRNKIKEKLKKIGQKIQGLLPKLAPRTDY. (TIF) [file pone.0197742.s001.tif]
