# Supplementary material for: Dissection of the antimicrobial and hemolytic activity of Cap18: Generation of Cap18 derivatives with enhanced specificity
Source: PLoS One. 2018 May 31;13(5):e0197742. doi: 10.1371/journal.pone.0197742 (PMC5978884; doi:10.1371/journal.pone.0197742)
Supplement: S1 Table — (DOCX) [file pone.0197742.s002.docx]

**S1 Table - Antimicrobial Peptides used in this study**

**Cap18 wild-type peptides**

| Name | | Amino Acid Sequence | Purity | Solvent | Company |
| --- | --- | --- | --- | --- | --- |
| Cap18 - pure | Original | GLRKRLRKFRNKIKEKLKKIGQKIQGLLPKLAPRTDY | ≥ 89.5% | DMSO | Genscript |
| Cap18 - library | Original | GLRKRLRKFRNKIKEKLKKIGQKIQGLLPKLAPRTDY | 47.5% | DMSO | Genscript |

**Cap18 variant peptides - Library Peptides**

| Name | Position | Substitution | Sequence | Purity | Solvent | Company |
| --- | --- | --- | --- | --- | --- | --- |
| 2 | 1 | (G1A) | ALRKRLRKFRNKIKEKLKKIGQKIQGLLPKLAPRTDY | 62.4 | DMSO | Genscript |
| 3 | 1 | (G1C) | CLRKRLRKFRNKIKEKLKKIGQKIQGLLPKLAPRTDY | 47.4 | DMSO | Genscript |
| 4 | 1 | (G1D) | DLRKRLRKFRNKIKEKLKKIGQKIQGLLPKLAPRTDY | 39.6 | DMSO | Genscript |
| 5 | 1 | (G1E) | ELRKRLRKFRNKIKEKLKKIGQKIQGLLPKLAPRTDY | 38.4 | DMSO | Genscript |
| 6 | 1 | (G1F) | FLRKRLRKFRNKIKEKLKKIGQKIQGLLPKLAPRTDY | 48.4 | DMSO | Genscript |
| 7 | 1 | (G1H) | HLRKRLRKFRNKIKEKLKKIGQKIQGLLPKLAPRTDY | 41.7 | DMSO | Genscript |
| 8 | 1 | (G1I) | ILRKRLRKFRNKIKEKLKKIGQKIQGLLPKLAPRTDY | 43.3 | DMSO | Genscript |
| 9 | 1 | (G1K) | KLRKRLRKFRNKIKEKLKKIGQKIQGLLPKLAPRTDY | 62.8 | DMSO | Genscript |
| 10 | 1 | (G1L) | LLRKRLRKFRNKIKEKLKKIGQKIQGLLPKLAPRTDY | 47.3 | DMSO | Genscript |
| 11 | 1 | (G1M) | MLRKRLRKFRNKIKEKLKKIGQKIQGLLPKLAPRTDY | 41.2 | DMSO | Genscript |
| 12 | 1 | (G1N) | NLRKRLRKFRNKIKEKLKKIGQKIQGLLPKLAPRTDY | 46 | DMSO | Genscript |
| 13 | 1 | (G1P) | PLRKRLRKFRNKIKEKLKKIGQKIQGLLPKLAPRTDY | 48.3 | DMSO | Genscript |
| 14 | 1 | (G1Q) | QLRKRLRKFRNKIKEKLKKIGQKIQGLLPKLAPRTDY | 31.1 | DMSO | Genscript |
| 15 | 1 | (G1R) | RLRKRLRKFRNKIKEKLKKIGQKIQGLLPKLAPRTDY | 43.1 | DMSO | Genscript |
| 16 | 1 | (G1S) | SLRKRLRKFRNKIKEKLKKIGQKIQGLLPKLAPRTDY | 46.4 | DMSO | Genscript |
| 17 | 1 | (G1T) | TLRKRLRKFRNKIKEKLKKIGQKIQGLLPKLAPRTDY | 33.8 | DMSO | Genscript |
| 18 | 1 | (G1V) | VLRKRLRKFRNKIKEKLKKIGQKIQGLLPKLAPRTDY | 49.3 | DMSO | Genscript |
| 19 | 1 | (G1W) | WLRKRLRKFRNKIKEKLKKIGQKIQGLLPKLAPRTDY | 58.8 | DMSO | Genscript |
| 20 | 1 | (G1Y) | YLRKRLRKFRNKIKEKLKKIGQKIQGLLPKLAPRTDY | 53.3 | DMSO | Genscript |
| 21 | 2 | (L2A) | GARKRLRKFRNKIKEKLKKIGQKIQGLLPKLAPRTDY | 53.9 | DMSO | Genscript |
| 22 | 2 | (L2C) | GCRKRLRKFRNKIKEKLKKIGQKIQGLLPKLAPRTDY | 38.7 | DMSO | Genscript |
| 23 | 2 | (L2D) | GDRKRLRKFRNKIKEKLKKIGQKIQGLLPKLAPRTDY | 50 | DMSO | Genscript |
| 24 | 2 | (L2E) | GERKRLRKFRNKIKEKLKKIGQKIQGLLPKLAPRTDY | 50.4 | DMSO | Genscript |
| 25 | 2 | (L2F) | GFRKRLRKFRNKIKEKLKKIGQKIQGLLPKLAPRTDY | 47.6 | DMSO | Genscript |
| 26 | 2 | (L2G) | GGRKRLRKFRNKIKEKLKKIGQKIQGLLPKLAPRTDY | 57.4 | DMSO | Genscript |
| 27 | 2 | (L2H) | GHRKRLRKFRNKIKEKLKKIGQKIQGLLPKLAPRTDY | 57.9 | DMSO | Genscript |
| 28 | 2 | (L2I) | GIRKRLRKFRNKIKEKLKKIGQKIQGLLPKLAPRTDY | 29.2 | DMSO | Genscript |
| 29 | 2 | (L2K) | GKRKRLRKFRNKIKEKLKKIGQKIQGLLPKLAPRTDY | 44.4 | DMSO | Genscript |
| 30 | 2 | (L2M) | GMRKRLRKFRNKIKEKLKKIGQKIQGLLPKLAPRTDY | 46 | DMSO | Genscript |
| 31 | 2 | (L2N) | GNRKRLRKFRNKIKEKLKKIGQKIQGLLPKLAPRTDY | 44.4 | DMSO | Genscript |
| 32 | 2 | (L2P) | GPRKRLRKFRNKIKEKLKKIGQKIQGLLPKLAPRTDY | 43.3 | DMSO | Genscript |
| 33 | 2 | (L2Q) | GQRKRLRKFRNKIKEKLKKIGQKIQGLLPKLAPRTDY | 62.7 | DMSO | Genscript |
| 34 | 2 | (L2R) | GRRKRLRKFRNKIKEKLKKIGQKIQGLLPKLAPRTDY | 50.9 | DMSO | Genscript |
| 35 | 2 | (L2S) | GSRKRLRKFRNKIKEKLKKIGQKIQGLLPKLAPRTDY | 49.1 | DMSO | Genscript |
| 36 | 2 | (L2T) | GTRKRLRKFRNKIKEKLKKIGQKIQGLLPKLAPRTDY | 29.6 | DMSO | Genscript |
| 37 | 2 | (L2V) | GVRKRLRKFRNKIKEKLKKIGQKIQGLLPKLAPRTDY | 39.2 | DMSO | Genscript |
| 38 | 2 | (L2W) | GWRKRLRKFRNKIKEKLKKIGQKIQGLLPKLAPRTDY | 57.7 | DMSO | Genscript |
| 39 | 2 | (L2Y) | GYRKRLRKFRNKIKEKLKKIGQKIQGLLPKLAPRTDY | 39.1 | DMSO | Genscript |
| 40 | 3 | (R3A) | GLAKRLRKFRNKIKEKLKKIGQKIQGLLPKLAPRTDY | 49.9 | DMSO | Genscript |
| 41 | 3 | (R3C) | GLCKRLRKFRNKIKEKLKKIGQKIQGLLPKLAPRTDY | 50.5 | DMSO | Genscript |
| 42 | 3 | (R3D) | GLDKRLRKFRNKIKEKLKKIGQKIQGLLPKLAPRTDY | 56.3 | DMSO | Genscript |
| 43 | 3 | (R3E) | GLEKRLRKFRNKIKEKLKKIGQKIQGLLPKLAPRTDY | 44.6 | DMSO | Genscript |
| 44 | 3 | (R3F) | GLFKRLRKFRNKIKEKLKKIGQKIQGLLPKLAPRTDY | 71.1 | DMSO | Genscript |
| 45 | 3 | (R3G) | GLGKRLRKFRNKIKEKLKKIGQKIQGLLPKLAPRTDY | 49.5 | DMSO | Genscript |
| 46 | 3 | (R3H) | GLHKRLRKFRNKIKEKLKKIGQKIQGLLPKLAPRTDY | 45.4 | DMSO | Genscript |
| 47 | 3 | (R3I) | GLIKRLRKFRNKIKEKLKKIGQKIQGLLPKLAPRTDY | 41.8 | DMSO | Genscript |
| 48 | 3 | (R3K) | GLKKRLRKFRNKIKEKLKKIGQKIQGLLPKLAPRTDY | 60.1 | DMSO | Genscript |
| 49 | 3 | (R3L) | GLLKRLRKFRNKIKEKLKKIGQKIQGLLPKLAPRTDY | 57.4 | DMSO | Genscript |
| 50 | 3 | (R3M) | GLMKRLRKFRNKIKEKLKKIGQKIQGLLPKLAPRTDY | 41 | DMSO | Genscript |
| 51 | 3 | (R3N) | GLNKRLRKFRNKIKEKLKKIGQKIQGLLPKLAPRTDY | 53.4 | DMSO | Genscript |
| 52 | 3 | (R3P) | GLPKRLRKFRNKIKEKLKKIGQKIQGLLPKLAPRTDY | 35.7 | DMSO | Genscript |
| 53 | 3 | (R3Q) | GLQKRLRKFRNKIKEKLKKIGQKIQGLLPKLAPRTDY | 45.8 | DMSO | Genscript |
| 54 | 3 | (R3S) | GLSKRLRKFRNKIKEKLKKIGQKIQGLLPKLAPRTDY | 48 | DMSO | Genscript |
| 55 | 3 | (R3T) | GLTKRLRKFRNKIKEKLKKIGQKIQGLLPKLAPRTDY | 39.6 | DMSO | Genscript |
| 56 | 3 | (R3V) | GLVKRLRKFRNKIKEKLKKIGQKIQGLLPKLAPRTDY | 44 | DMSO | Genscript |
| 57 | 3 | (R3W) | GLWKRLRKFRNKIKEKLKKIGQKIQGLLPKLAPRTDY | 53.3 | DMSO | Genscript |
| 58 | 3 | (R3Y) | GLYKRLRKFRNKIKEKLKKIGQKIQGLLPKLAPRTDY | 46.2 | DMSO | Genscript |
| 59 | 4 | (K4A) | GLRARLRKFRNKIKEKLKKIGQKIQGLLPKLAPRTDY | 46.4 | DMSO | Genscript |
| 60 | 4 | (K4C) | GLRCRLRKFRNKIKEKLKKIGQKIQGLLPKLAPRTDY | 38.9 | DMSO | Genscript |
| 61 | 4 | (K4D) | GLRDRLRKFRNKIKEKLKKIGQKIQGLLPKLAPRTDY | 45.5 | DMSO | Genscript |
| 62 | 4 | (K4E) | GLRERLRKFRNKIKEKLKKIGQKIQGLLPKLAPRTDY | 46.9 | DMSO | Genscript |
| 63 | 4 | (K4F) | GLRFRLRKFRNKIKEKLKKIGQKIQGLLPKLAPRTDY | 47.4 | DMSO | Genscript |
| 64 | 4 | (K4G) | GLRGRLRKFRNKIKEKLKKIGQKIQGLLPKLAPRTDY | 44.9 | DMSO | Genscript |
| 65 | 4 | (K4H) | GLRHRLRKFRNKIKEKLKKIGQKIQGLLPKLAPRTDY | 49.9 | DMSO | Genscript |
| 66 | 4 | (K4I) | GLRIRLRKFRNKIKEKLKKIGQKIQGLLPKLAPRTDY | 46.9 | DMSO | Genscript |
| 67 | 4 | (K4L) | GLRLRLRKFRNKIKEKLKKIGQKIQGLLPKLAPRTDY | 37.7 | DMSO | Genscript |
| 68 | 4 | (K4M) | GLRMRLRKFRNKIKEKLKKIGQKIQGLLPKLAPRTDY | 59.6 | DMSO | Genscript |
| 69 | 4 | (K4N) | GLRNRLRKFRNKIKEKLKKIGQKIQGLLPKLAPRTDY | 57.5 | DMSO | Genscript |
| 70 | 4 | (K4P) | GLRPRLRKFRNKIKEKLKKIGQKIQGLLPKLAPRTDY | 30.6 | DMSO | Genscript |
| 71 | 4 | (K4Q) | GLRQRLRKFRNKIKEKLKKIGQKIQGLLPKLAPRTDY | 41.4 | DMSO | Genscript |
| 72 | 4 | (K4R) | GLRRRLRKFRNKIKEKLKKIGQKIQGLLPKLAPRTDY | 63.4 | DMSO | Genscript |
| 73 | 4 | (K4S) | GLRSRLRKFRNKIKEKLKKIGQKIQGLLPKLAPRTDY | 51.2 | DMSO | Genscript |
| 74 | 4 | (K4T) | GLRTRLRKFRNKIKEKLKKIGQKIQGLLPKLAPRTDY | 47.2 | DMSO | Genscript |
| 75 | 4 | (K4V) | GLRVRLRKFRNKIKEKLKKIGQKIQGLLPKLAPRTDY | 43 | DMSO | Genscript |
| 76 | 4 | (K4W) | GLRWRLRKFRNKIKEKLKKIGQKIQGLLPKLAPRTDY | 55.9 | DMSO | Genscript |
| 77 | 4 | (K4Y) | GLRYRLRKFRNKIKEKLKKIGQKIQGLLPKLAPRTDY | 49.8 | DMSO | Genscript |
| 78 | 5 | (R5A) | GLRKALRKFRNKIKEKLKKIGQKIQGLLPKLAPRTDY | 51 | DMSO | Genscript |
| 79 | 5 | (R5C) | GLRKCLRKFRNKIKEKLKKIGQKIQGLLPKLAPRTDY | 51.9 | DMSO | Genscript |
| 80 | 5 | (R5D) | GLRKDLRKFRNKIKEKLKKIGQKIQGLLPKLAPRTDY | 46.9 | DMSO | Genscript |
| 81 | 5 | (R5E) | GLRKELRKFRNKIKEKLKKIGQKIQGLLPKLAPRTDY | 46.3 | DMSO | Genscript |
| 82 | 5 | (R5F) | GLRKFLRKFRNKIKEKLKKIGQKIQGLLPKLAPRTDY | 44.1 | DMSO | Genscript |
| 83 | 5 | (R5G) | GLRKGLRKFRNKIKEKLKKIGQKIQGLLPKLAPRTDY | 47.5 | DMSO | Genscript |
| 84 | 5 | (R5H) | GLRKHLRKFRNKIKEKLKKIGQKIQGLLPKLAPRTDY | 47.5 | DMSO | Genscript |
| 85 | 5 | (R5I) | GLRKILRKFRNKIKEKLKKIGQKIQGLLPKLAPRTDY | 36.6 | DMSO | Genscript |
| 86 | 5 | (R5K) | GLRKKLRKFRNKIKEKLKKIGQKIQGLLPKLAPRTDY | 67.2 | DMSO | Genscript |
| 87 | 5 | (R5L) | GLRKLLRKFRNKIKEKLKKIGQKIQGLLPKLAPRTDY | 52.1 | DMSO | Genscript |
| 88 | 5 | (R5M) | GLRKMLRKFRNKIKEKLKKIGQKIQGLLPKLAPRTDY | 43.1 | DMSO | Genscript |
| 89 | 5 | (R5N) | GLRKNLRKFRNKIKEKLKKIGQKIQGLLPKLAPRTDY | 48.5 | DMSO | Genscript |
| 90 | 5 | (R5P) | GLRKPLRKFRNKIKEKLKKIGQKIQGLLPKLAPRTDY | 63.8 | DMSO | Genscript |
| 91 | 5 | (R5Q) | GLRKQLRKFRNKIKEKLKKIGQKIQGLLPKLAPRTDY | 35.8 | DMSO | Genscript |
| 92 | 5 | (R5S) | GLRKSLRKFRNKIKEKLKKIGQKIQGLLPKLAPRTDY | 48.4 | DMSO | Genscript |
| 93 | 5 | (R5T) | GLRKTLRKFRNKIKEKLKKIGQKIQGLLPKLAPRTDY | 21.9 | DMSO | Genscript |
| 94 | 5 | (R5V) | GLRKVLRKFRNKIKEKLKKIGQKIQGLLPKLAPRTDY | 45.4 | DMSO | Genscript |
| 95 | 5 | (R5W) | GLRKWLRKFRNKIKEKLKKIGQKIQGLLPKLAPRTDY | 39.2 | DMSO | Genscript |
| 96 | 5 | (R5Y) | GLRKYLRKFRNKIKEKLKKIGQKIQGLLPKLAPRTDY | 57.2 | DMSO | Genscript |
| 97 | 6 | (L6A) | GLRKRARKFRNKIKEKLKKIGQKIQGLLPKLAPRTDY | 53.5 | DMSO | Genscript |
| 98 | 6 | (L6C) | GLRKRCRKFRNKIKEKLKKIGQKIQGLLPKLAPRTDY | 46 | DMSO | Genscript |
| 99 | 6 | (L6D) | GLRKRDRKFRNKIKEKLKKIGQKIQGLLPKLAPRTDY | 56.2 | DMSO | Genscript |
| 100 | 6 | (L6E) | GLRKRERKFRNKIKEKLKKIGQKIQGLLPKLAPRTDY | 55.1 | DMSO | Genscript |
| 101 | 6 | (L6F) | GLRKRFRKFRNKIKEKLKKIGQKIQGLLPKLAPRTDY | 44.3 | DMSO | Genscript |
| 102 | 6 | (L6G) | GLRKRGRKFRNKIKEKLKKIGQKIQGLLPKLAPRTDY | 56.3 | DMSO | Genscript |
| 103 | 6 | (L6H) | GLRKRHRKFRNKIKEKLKKIGQKIQGLLPKLAPRTDY | 55.2 | DMSO | Genscript |
| 104 | 6 | (L6I) | GLRKRIRKFRNKIKEKLKKIGQKIQGLLPKLAPRTDY | 43.8 | DMSO | Genscript |
| 105 | 6 | (L6K) | GLRKRKRKFRNKIKEKLKKIGQKIQGLLPKLAPRTDY | 57 | DMSO | Genscript |
| 106 | 6 | (L6M) | GLRKRMRKFRNKIKEKLKKIGQKIQGLLPKLAPRTDY | 41.6 | DMSO | Genscript |
| 107 | 6 | (L6N) | GLRKRNRKFRNKIKEKLKKIGQKIQGLLPKLAPRTDY | 45.4 | DMSO | Genscript |
| 108 | 6 | (L6P) | GLRKRPRKFRNKIKEKLKKIGQKIQGLLPKLAPRTDY | 51.6 | DMSO | Genscript |
| 109 | 6 | (L6Q) | GLRKRQRKFRNKIKEKLKKIGQKIQGLLPKLAPRTDY | 52.1 | DMSO | Genscript |
| 110 | 6 | (L6R) | GLRKRRRKFRNKIKEKLKKIGQKIQGLLPKLAPRTDY | 58.1 | DMSO | Genscript |
| 111 | 6 | (L6S) | GLRKRSRKFRNKIKEKLKKIGQKIQGLLPKLAPRTDY | 56.8 | DMSO | Genscript |
| 112 | 6 | (L6T) | GLRKRTRKFRNKIKEKLKKIGQKIQGLLPKLAPRTDY | 38.4 | DMSO | Genscript |
| 113 | 6 | (L6V) | GLRKRVRKFRNKIKEKLKKIGQKIQGLLPKLAPRTDY | 38.1 | DMSO | Genscript |
| 114 | 6 | (L6W) | GLRKRWRKFRNKIKEKLKKIGQKIQGLLPKLAPRTDY | 46.5 | DMSO | Genscript |
| 115 | 6 | (L6Y) | GLRKRYRKFRNKIKEKLKKIGQKIQGLLPKLAPRTDY | 43.1 | DMSO | Genscript |
| 116 | 7 | (R7A) | GLRKRLAKFRNKIKEKLKKIGQKIQGLLPKLAPRTDY | 47.3 | DMSO | Genscript |
| 117 | 7 | (R7C) | GLRKRLCKFRNKIKEKLKKIGQKIQGLLPKLAPRTDY | 51.7 | DMSO | Genscript |
| 118 | 7 | (R7D) | GLRKRLDKFRNKIKEKLKKIGQKIQGLLPKLAPRTDY | 56.4 | DMSO | Genscript |
| 119 | 7 | (R7E) | GLRKRLEKFRNKIKEKLKKIGQKIQGLLPKLAPRTDY | 51.8 | DMSO | Genscript |
| 120 | 7 | (R7F) | GLRKRLFKFRNKIKEKLKKIGQKIQGLLPKLAPRTDY | 54.6 | DMSO | Genscript |
| 121 | 7 | (R7G) | GLRKRLGKFRNKIKEKLKKIGQKIQGLLPKLAPRTDY | 53.6 | DMSO | Genscript |
| 122 | 7 | (R7H) | GLRKRLHKFRNKIKEKLKKIGQKIQGLLPKLAPRTDY | 50.6 | DMSO | Genscript |
| 123 | 7 | (R7I) | GLRKRLIKFRNKIKEKLKKIGQKIQGLLPKLAPRTDY | 43.9 | DMSO | Genscript |
| 124 | 7 | (R7K) | GLRKRLKKFRNKIKEKLKKIGQKIQGLLPKLAPRTDY | 45.1 | DMSO | Genscript |
| 125 | 7 | (R7L) | GLRKRLLKFRNKIKEKLKKIGQKIQGLLPKLAPRTDY | 46.4 | DMSO | Genscript |
| 126 | 7 | (R7M) | GLRKRLMKFRNKIKEKLKKIGQKIQGLLPKLAPRTDY | 21.8 | DMSO | Genscript |
| 127 | 7 | (R7N) | GLRKRLNKFRNKIKEKLKKIGQKIQGLLPKLAPRTDY | 45.6 | DMSO | Genscript |
| 128 | 7 | (R7P) | GLRKRLPKFRNKIKEKLKKIGQKIQGLLPKLAPRTDY | 43.9 | DMSO | Genscript |
| 129 | 7 | (R7Q) | GLRKRLQKFRNKIKEKLKKIGQKIQGLLPKLAPRTDY | 51.7 | DMSO | Genscript |
| 130 | 7 | (R7S) | GLRKRLSKFRNKIKEKLKKIGQKIQGLLPKLAPRTDY | 47.6 | DMSO | Genscript |
| 131 | 7 | (R7T) | GLRKRLTKFRNKIKEKLKKIGQKIQGLLPKLAPRTDY | 48.4 | DMSO | Genscript |
| 132 | 7 | (R7V) | GLRKRLVKFRNKIKEKLKKIGQKIQGLLPKLAPRTDY | 67.2 | DMSO | Genscript |
| 133 | 7 | (R7W) | GLRKRLWKFRNKIKEKLKKIGQKIQGLLPKLAPRTDY | 54.2 | DMSO | Genscript |
| 134 | 7 | (R7Y) | GLRKRLYKFRNKIKEKLKKIGQKIQGLLPKLAPRTDY | 44.6 | DMSO | Genscript |
| 135 | 8 | (K8A) | GLRKRLRAFRNKIKEKLKKIGQKIQGLLPKLAPRTDY | 51.2 | DMSO | Genscript |
| 136 | 8 | (K8C) | GLRKRLRCFRNKIKEKLKKIGQKIQGLLPKLAPRTDY | 56.4 | DMSO | Genscript |
| 137 | 8 | (K8D) | GLRKRLRDFRNKIKEKLKKIGQKIQGLLPKLAPRTDY | 37.2 | DMSO | Genscript |
| 138 | 8 | (K8E) | GLRKRLREFRNKIKEKLKKIGQKIQGLLPKLAPRTDY | 48.1 | DMSO | Genscript |
| 139 | 8 | (K8F) | GLRKRLRFFRNKIKEKLKKIGQKIQGLLPKLAPRTDY | 50.8 | DMSO | Genscript |
| 140 | 8 | (K8G) | GLRKRLRGFRNKIKEKLKKIGQKIQGLLPKLAPRTDY | 41.2 | DMSO | Genscript |
| 141 | 8 | (K8H) | GLRKRLRHFRNKIKEKLKKIGQKIQGLLPKLAPRTDY | 61 | DMSO | Genscript |
| 142 | 8 | (K8I) | GLRKRLRIFRNKIKEKLKKIGQKIQGLLPKLAPRTDY | 43.7 | DMSO | Genscript |
| 143 | 8 | (K8L) | GLRKRLRLFRNKIKEKLKKIGQKIQGLLPKLAPRTDY | 38.4 | DMSO | Genscript |
| 144 | 8 | (K8M) | GLRKRLRMFRNKIKEKLKKIGQKIQGLLPKLAPRTDY | 43.3 | DMSO | Genscript |
| 145 | 8 | (K8N) | GLRKRLRNFRNKIKEKLKKIGQKIQGLLPKLAPRTDY | 47.8 | DMSO | Genscript |
| 146 | 8 | (K8P) | GLRKRLRPFRNKIKEKLKKIGQKIQGLLPKLAPRTDY | 45.2 | DMSO | Genscript |
| 147 | 8 | (K8Q) | GLRKRLRQFRNKIKEKLKKIGQKIQGLLPKLAPRTDY | 53.5 | DMSO | Genscript |
| 148 | 8 | (K8R) | GLRKRLRRFRNKIKEKLKKIGQKIQGLLPKLAPRTDY | 46.3 | DMSO | Genscript |
| 149 | 8 | (K8S) | GLRKRLRSFRNKIKEKLKKIGQKIQGLLPKLAPRTDY | 44.2 | DMSO | Genscript |
| 150 | 8 | (K8T) | GLRKRLRTFRNKIKEKLKKIGQKIQGLLPKLAPRTDY | 54.7 | DMSO | Genscript |
| 151 | 8 | (K8V) | GLRKRLRVFRNKIKEKLKKIGQKIQGLLPKLAPRTDY | 36.9 | DMSO | Genscript |
| 152 | 8 | (K8W) | GLRKRLRWFRNKIKEKLKKIGQKIQGLLPKLAPRTDY | 48.9 | DMSO | Genscript |
| 153 | 8 | (K8Y) | GLRKRLRYFRNKIKEKLKKIGQKIQGLLPKLAPRTDY | 50.5 | DMSO | Genscript |
| 154 | 9 | (F9A) | GLRKRLRKARNKIKEKLKKIGQKIQGLLPKLAPRTDY | 49.3 | DMSO | Genscript |
| 155 | 9 | (F9C) | GLRKRLRKCRNKIKEKLKKIGQKIQGLLPKLAPRTDY | 35 | DMSO | Genscript |
| 156 | 9 | (F9D) | GLRKRLRKDRNKIKEKLKKIGQKIQGLLPKLAPRTDY | 38.1 | DMSO | Genscript |
| 157 | 9 | (F9E) | GLRKRLRKERNKIKEKLKKIGQKIQGLLPKLAPRTDY | 55.5 | DMSO | Genscript |
| 158 | 9 | (F9G) | GLRKRLRKGRNKIKEKLKKIGQKIQGLLPKLAPRTDY | 67.6 | DMSO | Genscript |
| 159 | 9 | (F9H) | GLRKRLRKHRNKIKEKLKKIGQKIQGLLPKLAPRTDY | 68.2 | DMSO | Genscript |
| 160 | 9 | (F9I) | GLRKRLRKIRNKIKEKLKKIGQKIQGLLPKLAPRTDY | 53.6 | DMSO | Genscript |
| 161 | 9 | (F9K) | GLRKRLRKKRNKIKEKLKKIGQKIQGLLPKLAPRTDY | 50.8 | DMSO | Genscript |
| 162 | 9 | (F9L) | GLRKRLRKLRNKIKEKLKKIGQKIQGLLPKLAPRTDY | 53.4 | DMSO | Genscript |
| 163 | 9 | (F9M) | GLRKRLRKMRNKIKEKLKKIGQKIQGLLPKLAPRTDY | 60.6 | DMSO | Genscript |
| 164 | 9 | (F9N) | GLRKRLRKNRNKIKEKLKKIGQKIQGLLPKLAPRTDY | 73.7 | DMSO | Genscript |
| 165 | 9 | (F9P) | GLRKRLRKPRNKIKEKLKKIGQKIQGLLPKLAPRTDY | 56.6 | DMSO | Genscript |
| 166 | 9 | (F9Q) | GLRKRLRKQRNKIKEKLKKIGQKIQGLLPKLAPRTDY | 39.2 | DMSO | Genscript |
| 167 | 9 | (F9R) | GLRKRLRKRRNKIKEKLKKIGQKIQGLLPKLAPRTDY | 61.4 | DMSO | Genscript |
| 168 | 9 | (F9S) | GLRKRLRKSRNKIKEKLKKIGQKIQGLLPKLAPRTDY | 54.2 | DMSO | Genscript |
| 169 | 9 | (F9T) | GLRKRLRKTRNKIKEKLKKIGQKIQGLLPKLAPRTDY | 39.6 | DMSO | Genscript |
| 170 | 9 | (F9V) | GLRKRLRKVRNKIKEKLKKIGQKIQGLLPKLAPRTDY | 50 | DMSO | Genscript |
| 171 | 9 | (F9W) | GLRKRLRKWRNKIKEKLKKIGQKIQGLLPKLAPRTDY | 55.5 | DMSO | Genscript |
| 172 | 9 | (F9Y) | GLRKRLRKYRNKIKEKLKKIGQKIQGLLPKLAPRTDY | 41.7 | DMSO | Genscript |
| 173 | 10 | (R10A) | GLRKRLRKFANKIKEKLKKIGQKIQGLLPKLAPRTDY | 57.6 | DMSO | Genscript |
| 174 | 10 | (R10C) | GLRKRLRKFCNKIKEKLKKIGQKIQGLLPKLAPRTDY | 50.4 | DMSO | Genscript |
| 175 | 10 | (R10D) | GLRKRLRKFDNKIKEKLKKIGQKIQGLLPKLAPRTDY | 59.9 | DMSO | Genscript |
| 176 | 10 | (R10E) | GLRKRLRKFENKIKEKLKKIGQKIQGLLPKLAPRTDY | 47.1 | DMSO | Genscript |
| 177 | 10 | (R10F) | GLRKRLRKFFNKIKEKLKKIGQKIQGLLPKLAPRTDY | 44.6 | DMSO | Genscript |
| 178 | 10 | (R10G) | GLRKRLRKFGNKIKEKLKKIGQKIQGLLPKLAPRTDY | 53 | DMSO | Genscript |
| 179 | 10 | (R10H) | GLRKRLRKFHNKIKEKLKKIGQKIQGLLPKLAPRTDY | 41.4 | DMSO | Genscript |
| 180 | 10 | (R10I) | GLRKRLRKFINKIKEKLKKIGQKIQGLLPKLAPRTDY | 59.5 | DMSO | Genscript |
| 181 | 10 | (R10K) | GLRKRLRKFKNKIKEKLKKIGQKIQGLLPKLAPRTDY | 57.5 | DMSO | Genscript |
| 182 | 10 | (R10L) | GLRKRLRKFLNKIKEKLKKIGQKIQGLLPKLAPRTDY | 47.8 | DMSO | Genscript |
| 183 | 10 | (R10M) | GLRKRLRKFMNKIKEKLKKIGQKIQGLLPKLAPRTDY | 36.8 | DMSO | Genscript |
| 184 | 10 | (R10N) | GLRKRLRKFNNKIKEKLKKIGQKIQGLLPKLAPRTDY | 48.5 | DMSO | Genscript |
| 185 | 10 | (R10P) | GLRKRLRKFPNKIKEKLKKIGQKIQGLLPKLAPRTDY | 60.3 | DMSO | Genscript |
| 186 | 10 | (R10Q) | GLRKRLRKFQNKIKEKLKKIGQKIQGLLPKLAPRTDY | 57.3 | DMSO | Genscript |
| 187 | 10 | (R10S) | GLRKRLRKFSNKIKEKLKKIGQKIQGLLPKLAPRTDY | 59.3 | DMSO | Genscript |
| 188 | 10 | (R10T) | GLRKRLRKFTNKIKEKLKKIGQKIQGLLPKLAPRTDY | 59.5 | DMSO | Genscript |
| 189 | 10 | (R10V) | GLRKRLRKFVNKIKEKLKKIGQKIQGLLPKLAPRTDY | 41.8 | DMSO | Genscript |
| 190 | 10 | (R10W) | GLRKRLRKFWNKIKEKLKKIGQKIQGLLPKLAPRTDY | 57.7 | DMSO | Genscript |
| 191 | 10 | (R10Y) | GLRKRLRKFYNKIKEKLKKIGQKIQGLLPKLAPRTDY | 44.5 | DMSO | Genscript |
| 192 | 11 | (N11A) | GLRKRLRKFRAKIKEKLKKIGQKIQGLLPKLAPRTDY | 50.8 | DMSO | Genscript |
| 193 | 11 | (N11C) | GLRKRLRKFRCKIKEKLKKIGQKIQGLLPKLAPRTDY | 37.7 | DMSO | Genscript |
| 194 | 11 | (N11D) | GLRKRLRKFRDKIKEKLKKIGQKIQGLLPKLAPRTDY | 55.2 | DMSO | Genscript |
| 195 | 11 | (N11E) | GLRKRLRKFREKIKEKLKKIGQKIQGLLPKLAPRTDY | 48.8 | DMSO | Genscript |
| 196 | 11 | (N11F) | GLRKRLRKFRFKIKEKLKKIGQKIQGLLPKLAPRTDY | 51.6 | DMSO | Genscript |
| 197 | 11 | (N11G) | GLRKRLRKFRGKIKEKLKKIGQKIQGLLPKLAPRTDY | 49.4 | DMSO | Genscript |
| 198 | 11 | (N11H) | GLRKRLRKFRHKIKEKLKKIGQKIQGLLPKLAPRTDY | 55.9 | DMSO | Genscript |
| 199 | 11 | (N11I) | GLRKRLRKFRIKIKEKLKKIGQKIQGLLPKLAPRTDY | 54.5 | DMSO | Genscript |
| 200 | 11 | (N11K) | GLRKRLRKFRKKIKEKLKKIGQKIQGLLPKLAPRTDY | 54.3 | DMSO | Genscript |
| 201 | 11 | (N11L) | GLRKRLRKFRLKIKEKLKKIGQKIQGLLPKLAPRTDY | 66.2 | DMSO | Genscript |
| 202 | 11 | (N11M) | GLRKRLRKFRMKIKEKLKKIGQKIQGLLPKLAPRTDY | 57.4 | DMSO | Genscript |
| 203 | 11 | (N11P) | GLRKRLRKFRPKIKEKLKKIGQKIQGLLPKLAPRTDY | 57.9 | DMSO | Genscript |
| 204 | 11 | (N11Q) | GLRKRLRKFRQKIKEKLKKIGQKIQGLLPKLAPRTDY | 49.3 | DMSO | Genscript |
| 205 | 11 | (N11R) | GLRKRLRKFRRKIKEKLKKIGQKIQGLLPKLAPRTDY | 60.3 | DMSO | Genscript |
| 206 | 11 | (N11S) | GLRKRLRKFRSKIKEKLKKIGQKIQGLLPKLAPRTDY | 55.6 | DMSO | Genscript |
| 207 | 11 | (N11T) | GLRKRLRKFRTKIKEKLKKIGQKIQGLLPKLAPRTDY | 36.5 | DMSO | Genscript |
| 208 | 11 | (N11V) | GLRKRLRKFRVKIKEKLKKIGQKIQGLLPKLAPRTDY | 42.9 | DMSO | Genscript |
| 209 | 11 | (N11W) | GLRKRLRKFRWKIKEKLKKIGQKIQGLLPKLAPRTDY | 65.3 | DMSO | Genscript |
| 210 | 11 | (N11Y) | GLRKRLRKFRYKIKEKLKKIGQKIQGLLPKLAPRTDY | 51.4 | DMSO | Genscript |
| 211 | 12 | (K12A) | GLRKRLRKFRNAIKEKLKKIGQKIQGLLPKLAPRTDY | 48.4 | DMSO | Genscript |
| 212 | 12 | (K12C) | GLRKRLRKFRNCIKEKLKKIGQKIQGLLPKLAPRTDY | 53.1 | DMSO | Genscript |
| 213 | 12 | (K12D) | GLRKRLRKFRNDIKEKLKKIGQKIQGLLPKLAPRTDY | 56.6 | DMSO | Genscript |
| 214 | 12 | (K12E) | GLRKRLRKFRNEIKEKLKKIGQKIQGLLPKLAPRTDY | 51.3 | DMSO | Genscript |
| 215 | 12 | (K12F) | GLRKRLRKFRNFIKEKLKKIGQKIQGLLPKLAPRTDY | 66.4 | DMSO | Genscript |
| 216 | 12 | (K12G) | GLRKRLRKFRNGIKEKLKKIGQKIQGLLPKLAPRTDY | 54.8 | DMSO | Genscript |
| 217 | 12 | (K12H) | GLRKRLRKFRNHIKEKLKKIGQKIQGLLPKLAPRTDY | 55.1 | DMSO | Genscript |
| 218 | 12 | (K12I) | GLRKRLRKFRNIIKEKLKKIGQKIQGLLPKLAPRTDY | 52.1 | DMSO | Genscript |
| 219 | 12 | (K12L) | GLRKRLRKFRNLIKEKLKKIGQKIQGLLPKLAPRTDY | 49.6 | DMSO | Genscript |
| 220 | 12 | (K12M) | GLRKRLRKFRNMIKEKLKKIGQKIQGLLPKLAPRTDY | 49.9 | DMSO | Genscript |
| 221 | 12 | (K12N) | GLRKRLRKFRNNIKEKLKKIGQKIQGLLPKLAPRTDY | 45.6 | DMSO | Genscript |
| 222 | 12 | (K12P) | GLRKRLRKFRNPIKEKLKKIGQKIQGLLPKLAPRTDY | 54.3 | DMSO | Genscript |
| 223 | 12 | (K12Q) | GLRKRLRKFRNQIKEKLKKIGQKIQGLLPKLAPRTDY | 57.6 | DMSO | Genscript |
| 224 | 12 | (K12R) | GLRKRLRKFRNRIKEKLKKIGQKIQGLLPKLAPRTDY | 41.3 | DMSO | Genscript |
| 225 | 12 | (K12S) | GLRKRLRKFRNSIKEKLKKIGQKIQGLLPKLAPRTDY | 37.1 | DMSO | Genscript |
| 226 | 12 | (K12T) | GLRKRLRKFRNTIKEKLKKIGQKIQGLLPKLAPRTDY | 60.2 | DMSO | Genscript |
| 227 | 12 | (K12V) | GLRKRLRKFRNVIKEKLKKIGQKIQGLLPKLAPRTDY | 56.5 | DMSO | Genscript |
| 228 | 12 | (K12W) | GLRKRLRKFRNWIKEKLKKIGQKIQGLLPKLAPRTDY | 51.1 | DMSO | Genscript |
| 229 | 12 | (K12Y) | GLRKRLRKFRNYIKEKLKKIGQKIQGLLPKLAPRTDY | 52.8 | DMSO | Genscript |
| 230 | 13 | (I13A) | GLRKRLRKFRNKAKEKLKKIGQKIQGLLPKLAPRTDY | 61.6 | DMSO | Genscript |
| 231 | 13 | (I13C) | GLRKRLRKFRNKCKEKLKKIGQKIQGLLPKLAPRTDY | 71.2 | DMSO | Genscript |
| 232 | 13 | (I13D) | GLRKRLRKFRNKDKEKLKKIGQKIQGLLPKLAPRTDY | 69.4 | DMSO | Genscript |
| 233 | 13 | (I13E) | GLRKRLRKFRNKEKEKLKKIGQKIQGLLPKLAPRTDY | 48.7 | DMSO | Genscript |
| 234 | 13 | (I13F) | GLRKRLRKFRNKFKEKLKKIGQKIQGLLPKLAPRTDY | 43 | DMSO | Genscript |
| 235 | 13 | (I13G) | GLRKRLRKFRNKGKEKLKKIGQKIQGLLPKLAPRTDY | 42.1 | DMSO | Genscript |
| 236 | 13 | (I13H) | GLRKRLRKFRNKHKEKLKKIGQKIQGLLPKLAPRTDY | 48.5 | DMSO | Genscript |
| 237 | 13 | (I13K) | GLRKRLRKFRNKKKEKLKKIGQKIQGLLPKLAPRTDY | 57.3 | DMSO | Genscript |
| 238 | 13 | (I13L) | GLRKRLRKFRNKLKEKLKKIGQKIQGLLPKLAPRTDY | 69.5 | DMSO | Genscript |
| 239 | 13 | (I13M) | GLRKRLRKFRNKMKEKLKKIGQKIQGLLPKLAPRTDY | 45.1 | DMSO | Genscript |
| 240 | 13 | (I13N) | GLRKRLRKFRNKNKEKLKKIGQKIQGLLPKLAPRTDY | 56.9 | DMSO | Genscript |
| 241 | 13 | (I13P) | GLRKRLRKFRNKPKEKLKKIGQKIQGLLPKLAPRTDY | 69.9 | DMSO | Genscript |
| 242 | 13 | (I13Q) | GLRKRLRKFRNKQKEKLKKIGQKIQGLLPKLAPRTDY | 45.6 | DMSO | Genscript |
| 243 | 13 | (I13R) | GLRKRLRKFRNKRKEKLKKIGQKIQGLLPKLAPRTDY | 59.6 | DMSO | Genscript |
| 244 | 13 | (I13S) | GLRKRLRKFRNKSKEKLKKIGQKIQGLLPKLAPRTDY | 48.3 | DMSO | Genscript |
| 245 | 13 | (I13T) | GLRKRLRKFRNKTKEKLKKIGQKIQGLLPKLAPRTDY | 42.4 | DMSO | Genscript |
| 246 | 13 | (I13V) | GLRKRLRKFRNKVKEKLKKIGQKIQGLLPKLAPRTDY | 57 | DMSO | Genscript |
| 247 | 13 | (I13W) | GLRKRLRKFRNKWKEKLKKIGQKIQGLLPKLAPRTDY | 66.6 | DMSO | Genscript |
| 248 | 13 | (I13Y) | GLRKRLRKFRNKYKEKLKKIGQKIQGLLPKLAPRTDY | 56 | DMSO | Genscript |
| 249 | 14 | (K14A) | GLRKRLRKFRNKIAEKLKKIGQKIQGLLPKLAPRTDY | 49.7 | DMSO | Genscript |
| 250 | 14 | (K14C) | GLRKRLRKFRNKICEKLKKIGQKIQGLLPKLAPRTDY | 30.6 | DMSO | Genscript |
| 251 | 14 | (K14D) | GLRKRLRKFRNKIDEKLKKIGQKIQGLLPKLAPRTDY | 77.4 | DMSO | Genscript |
| 252 | 14 | (K14E) | GLRKRLRKFRNKIEEKLKKIGQKIQGLLPKLAPRTDY | 57.9 | DMSO | Genscript |
| 253 | 14 | (K14F) | GLRKRLRKFRNKIFEKLKKIGQKIQGLLPKLAPRTDY | 46.4 | DMSO | Genscript |
| 254 | 14 | (K14G) | GLRKRLRKFRNKIGEKLKKIGQKIQGLLPKLAPRTDY | 47.8 | DMSO | Genscript |
| 255 | 14 | (K14H) | GLRKRLRKFRNKIHEKLKKIGQKIQGLLPKLAPRTDY | 51.2 | DMSO | Genscript |
| 256 | 14 | (K14I) | GLRKRLRKFRNKIIEKLKKIGQKIQGLLPKLAPRTDY | 47 | DMSO | Genscript |
| 257 | 14 | (K14L) | GLRKRLRKFRNKILEKLKKIGQKIQGLLPKLAPRTDY | 58.2 | DMSO | Genscript |
| 258 | 14 | (K14M) | GLRKRLRKFRNKIMEKLKKIGQKIQGLLPKLAPRTDY | 60.1 | DMSO | Genscript |
| 259 | 14 | (K14N) | GLRKRLRKFRNKINEKLKKIGQKIQGLLPKLAPRTDY | 49.7 | DMSO | Genscript |
| 260 | 14 | (K14P) | GLRKRLRKFRNKIPEKLKKIGQKIQGLLPKLAPRTDY | 43.9 | DMSO | Genscript |
| 261 | 14 | (K14Q) | GLRKRLRKFRNKIQEKLKKIGQKIQGLLPKLAPRTDY | 49.8 | DMSO | Genscript |
| 262 | 14 | (K14R) | GLRKRLRKFRNKIREKLKKIGQKIQGLLPKLAPRTDY | 45.4 | DMSO | Genscript |
| 263 | 14 | (K14S) | GLRKRLRKFRNKISEKLKKIGQKIQGLLPKLAPRTDY | 30.2 | DMSO | Genscript |
| 264 | 14 | (K14T) | GLRKRLRKFRNKITEKLKKIGQKIQGLLPKLAPRTDY | 44.2 | DMSO | Genscript |
| 265 | 14 | (K14V) | GLRKRLRKFRNKIVEKLKKIGQKIQGLLPKLAPRTDY | 52 | DMSO | Genscript |
| 266 | 14 | (K14W) | GLRKRLRKFRNKIWEKLKKIGQKIQGLLPKLAPRTDY | 59.3 | DMSO | Genscript |
| 267 | 14 | (K14Y) | GLRKRLRKFRNKIYEKLKKIGQKIQGLLPKLAPRTDY | 47 | DMSO | Genscript |
| 268 | 15 | (E15A) | GLRKRLRKFRNKIKAKLKKIGQKIQGLLPKLAPRTDY | 41.4 | DMSO | Genscript |
| 269 | 15 | (E15C) | GLRKRLRKFRNKIKCKLKKIGQKIQGLLPKLAPRTDY | 53.4 | DMSO | Genscript |
| 270 | 15 | (E15D) | GLRKRLRKFRNKIKDKLKKIGQKIQGLLPKLAPRTDY | 50.1 | DMSO | Genscript |
| 271 | 15 | (E15F) | GLRKRLRKFRNKIKFKLKKIGQKIQGLLPKLAPRTDY | 39.4 | DMSO | Genscript |
| 272 | 15 | (E15G) | GLRKRLRKFRNKIKGKLKKIGQKIQGLLPKLAPRTDY | 61.4 | DMSO | Genscript |
| 273 | 15 | (E15H) | GLRKRLRKFRNKIKHKLKKIGQKIQGLLPKLAPRTDY | 50.6 | DMSO | Genscript |
| 274 | 15 | (E15I) | GLRKRLRKFRNKIKIKLKKIGQKIQGLLPKLAPRTDY | 52.2 | DMSO | Genscript |
| 275 | 15 | (E15K) | GLRKRLRKFRNKIKKKLKKIGQKIQGLLPKLAPRTDY | 43 | DMSO | Genscript |
| 276 | 15 | (E15L) | GLRKRLRKFRNKIKLKLKKIGQKIQGLLPKLAPRTDY | 59 | DMSO | Genscript |
| 277 | 15 | (E15M) | GLRKRLRKFRNKIKMKLKKIGQKIQGLLPKLAPRTDY | 44.1 | DMSO | Genscript |
| 278 | 15 | (E15N) | GLRKRLRKFRNKIKNKLKKIGQKIQGLLPKLAPRTDY | 52.6 | DMSO | Genscript |
| 279 | 15 | (E15P) | GLRKRLRKFRNKIKPKLKKIGQKIQGLLPKLAPRTDY | 60.6 | DMSO | Genscript |
| 280 | 15 | (E15Q) | GLRKRLRKFRNKIKQKLKKIGQKIQGLLPKLAPRTDY | 28.3 | DMSO | Genscript |
| 281 | 15 | (E15R) | GLRKRLRKFRNKIKRKLKKIGQKIQGLLPKLAPRTDY | 52.2 | DMSO | Genscript |
| 282 | 15 | (E15S) | GLRKRLRKFRNKIKSKLKKIGQKIQGLLPKLAPRTDY | 53.1 | DMSO | Genscript |
| 283 | 15 | (E15T) | GLRKRLRKFRNKIKTKLKKIGQKIQGLLPKLAPRTDY | 42.5 | DMSO | Genscript |
| 284 | 15 | (E15V) | GLRKRLRKFRNKIKVKLKKIGQKIQGLLPKLAPRTDY | 47.7 | DMSO | Genscript |
| 285 | 15 | (E15W) | GLRKRLRKFRNKIKWKLKKIGQKIQGLLPKLAPRTDY | 65.6 | DMSO | Genscript |
| 286 | 15 | (E15Y) | GLRKRLRKFRNKIKYKLKKIGQKIQGLLPKLAPRTDY | 53.3 | DMSO | Genscript |
| 287 | 16 | (K16A) | GLRKRLRKFRNKIKEALKKIGQKIQGLLPKLAPRTDY | 44.1 | DMSO | Genscript |
| 288 | 16 | (K16C) | GLRKRLRKFRNKIKECLKKIGQKIQGLLPKLAPRTDY | 43.8 | DMSO | Genscript |
| 289 | 16 | (K16D) | GLRKRLRKFRNKIKEDLKKIGQKIQGLLPKLAPRTDY | 43.3 | DMSO | Genscript |
| 290 | 16 | (K16E) | GLRKRLRKFRNKIKEELKKIGQKIQGLLPKLAPRTDY | 52.4 | DMSO | Genscript |
| 291 | 16 | (K16F) | GLRKRLRKFRNKIKEFLKKIGQKIQGLLPKLAPRTDY | 49.3 | DMSO | Genscript |
| 292 | 16 | (K16G) | GLRKRLRKFRNKIKEGLKKIGQKIQGLLPKLAPRTDY | 42.3 | DMSO | Genscript |
| 293 | 16 | (K16H) | GLRKRLRKFRNKIKEHLKKIGQKIQGLLPKLAPRTDY | 40.5 | DMSO | Genscript |
| 294 | 16 | (K16I) | GLRKRLRKFRNKIKEILKKIGQKIQGLLPKLAPRTDY | 42.4 | DMSO | Genscript |
| 295 | 16 | (K16L) | GLRKRLRKFRNKIKELLKKIGQKIQGLLPKLAPRTDY | 45.4 | DMSO | Genscript |
| 296 | 16 | (K16M) | GLRKRLRKFRNKIKEMLKKIGQKIQGLLPKLAPRTDY | 60 | DMSO | Genscript |
| 297 | 16 | (K16N) | GLRKRLRKFRNKIKENLKKIGQKIQGLLPKLAPRTDY | 47.3 | DMSO | Genscript |
| 298 | 16 | (K16P) | GLRKRLRKFRNKIKEPLKKIGQKIQGLLPKLAPRTDY | 57.7 | DMSO | Genscript |
| 299 | 16 | (K16Q) | GLRKRLRKFRNKIKEQLKKIGQKIQGLLPKLAPRTDY | 46.5 | DMSO | Genscript |
| 300 | 16 | (K16R) | GLRKRLRKFRNKIKERLKKIGQKIQGLLPKLAPRTDY | 56.9 | DMSO | Genscript |
| 301 | 16 | (K16S) | GLRKRLRKFRNKIKESLKKIGQKIQGLLPKLAPRTDY | 30.5 | DMSO | Genscript |
| 302 | 16 | (K16T) | GLRKRLRKFRNKIKETLKKIGQKIQGLLPKLAPRTDY | 57.5 | DMSO | Genscript |
| 303 | 16 | (K16V) | GLRKRLRKFRNKIKEVLKKIGQKIQGLLPKLAPRTDY | 56.5 | DMSO | Genscript |
| 304 | 16 | (K16W) | GLRKRLRKFRNKIKEWLKKIGQKIQGLLPKLAPRTDY | 46.8 | DMSO | Genscript |
| 305 | 16 | (K16Y) | GLRKRLRKFRNKIKEYLKKIGQKIQGLLPKLAPRTDY | 57.4 | DMSO | Genscript |
| 306 | 17 | (L17A) | GLRKRLRKFRNKIKEKAKKIGQKIQGLLPKLAPRTDY | 48.9 | DMSO | Genscript |
| 307 | 17 | (L17C) | GLRKRLRKFRNKIKEKCKKIGQKIQGLLPKLAPRTDY | 65.9 | DMSO | Genscript |
| 308 | 17 | (L17D) | GLRKRLRKFRNKIKEKDKKIGQKIQGLLPKLAPRTDY | 30.7 | DMSO | Genscript |
| 309 | 17 | (L17E) | GLRKRLRKFRNKIKEKEKKIGQKIQGLLPKLAPRTDY | 73.2 | DMSO | Genscript |
| 310 | 17 | (L17F) | GLRKRLRKFRNKIKEKFKKIGQKIQGLLPKLAPRTDY | 44.5 | DMSO | Genscript |
| 311 | 17 | (L17G) | GLRKRLRKFRNKIKEKGKKIGQKIQGLLPKLAPRTDY | 49.1 | DMSO | Genscript |
| 312 | 17 | (L17H) | GLRKRLRKFRNKIKEKHKKIGQKIQGLLPKLAPRTDY | 51.3 | DMSO | Genscript |
| 313 | 17 | (L17I) | GLRKRLRKFRNKIKEKIKKIGQKIQGLLPKLAPRTDY | 15.8 | DMSO | Genscript |
| 314 | 17 | (L17K) | GLRKRLRKFRNKIKEKKKKIGQKIQGLLPKLAPRTDY | 30.4 | DMSO | Genscript |
| 315 | 17 | (L17M) | GLRKRLRKFRNKIKEKMKKIGQKIQGLLPKLAPRTDY | 49.3 | DMSO | Genscript |
| 316 | 17 | (L17N) | GLRKRLRKFRNKIKEKNKKIGQKIQGLLPKLAPRTDY | 45.4 | DMSO | Genscript |
| 317 | 17 | (L17P) | GLRKRLRKFRNKIKEKPKKIGQKIQGLLPKLAPRTDY | 55.6 | DMSO | Genscript |
| 318 | 17 | (L17Q) | GLRKRLRKFRNKIKEKQKKIGQKIQGLLPKLAPRTDY | 53.5 | DMSO | Genscript |
| 319 | 17 | (L17R) | GLRKRLRKFRNKIKEKRKKIGQKIQGLLPKLAPRTDY | 39.8 | DMSO | Genscript |
| 320 | 17 | (L17S) | GLRKRLRKFRNKIKEKSKKIGQKIQGLLPKLAPRTDY | 29.6 | DMSO | Genscript |
| 321 | 17 | (L17T) | GLRKRLRKFRNKIKEKTKKIGQKIQGLLPKLAPRTDY | 32.6 | DMSO | Genscript |
| 322 | 17 | (L17V) | GLRKRLRKFRNKIKEKVKKIGQKIQGLLPKLAPRTDY | 33.7 | DMSO | Genscript |
| 323 | 17 | (L17W) | GLRKRLRKFRNKIKEKWKKIGQKIQGLLPKLAPRTDY | 52.6 | DMSO | Genscript |
| 324 | 17 | (L17Y) | GLRKRLRKFRNKIKEKYKKIGQKIQGLLPKLAPRTDY | 50.5 | DMSO | Genscript |
| 325 | 18 | (K18A) | GLRKRLRKFRNKIKEKLAKIGQKIQGLLPKLAPRTDY | 30.9 | DMSO | Genscript |
| 326 | 18 | (K18C) | GLRKRLRKFRNKIKEKLCKIGQKIQGLLPKLAPRTDY | 38.2 | DMSO | Genscript |
| 327 | 18 | (K18D) | GLRKRLRKFRNKIKEKLDKIGQKIQGLLPKLAPRTDY | 55.3 | DMSO | Genscript |
| 328 | 18 | (K18E) | GLRKRLRKFRNKIKEKLEKIGQKIQGLLPKLAPRTDY | 56.2 | DMSO | Genscript |
| 329 | 18 | (K18F) | GLRKRLRKFRNKIKEKLFKIGQKIQGLLPKLAPRTDY | 53.2 | DMSO | Genscript |
| 330 | 18 | (K18G) | GLRKRLRKFRNKIKEKLGKIGQKIQGLLPKLAPRTDY | 47.2 | DMSO | Genscript |
| 331 | 18 | (K18H) | GLRKRLRKFRNKIKEKLHKIGQKIQGLLPKLAPRTDY | 37.2 | DMSO | Genscript |
| 332 | 18 | (K18I) | GLRKRLRKFRNKIKEKLIKIGQKIQGLLPKLAPRTDY | 42.9 | DMSO | Genscript |
| 333 | 18 | (K18L) | GLRKRLRKFRNKIKEKLLKIGQKIQGLLPKLAPRTDY | 48.3 | DMSO | Genscript |
| 334 | 18 | (K18M) | GLRKRLRKFRNKIKEKLMKIGQKIQGLLPKLAPRTDY | 44.1 | DMSO | Genscript |
| 335 | 18 | (K18N) | GLRKRLRKFRNKIKEKLNKIGQKIQGLLPKLAPRTDY | 47.7 | DMSO | Genscript |
| 336 | 18 | (K18P) | GLRKRLRKFRNKIKEKLPKIGQKIQGLLPKLAPRTDY | 58.9 | DMSO | Genscript |
| 337 | 18 | (K18Q) | GLRKRLRKFRNKIKEKLQKIGQKIQGLLPKLAPRTDY | 25.8 | DMSO | Genscript |
| 338 | 18 | (K18R) | GLRKRLRKFRNKIKEKLRKIGQKIQGLLPKLAPRTDY | 51.2 | DMSO | Genscript |
| 339 | 18 | (K18S) | GLRKRLRKFRNKIKEKLSKIGQKIQGLLPKLAPRTDY | 48.4 | DMSO | Genscript |
| 340 | 18 | (K18T) | GLRKRLRKFRNKIKEKLTKIGQKIQGLLPKLAPRTDY | 31.5 | DMSO | Genscript |
| 341 | 18 | (K18V) | GLRKRLRKFRNKIKEKLVKIGQKIQGLLPKLAPRTDY | 40.5 | DMSO | Genscript |
| 342 | 18 | (K18W) | GLRKRLRKFRNKIKEKLWKIGQKIQGLLPKLAPRTDY | 52.1 | DMSO | Genscript |
| 343 | 18 | (K18Y) | GLRKRLRKFRNKIKEKLYKIGQKIQGLLPKLAPRTDY | 41.4 | DMSO | Genscript |
| 344 | 19 | (K19A) | GLRKRLRKFRNKIKEKLKAIGQKIQGLLPKLAPRTDY | 49.8 | DMSO | Genscript |
| 345 | 19 | (K19C) | GLRKRLRKFRNKIKEKLKCIGQKIQGLLPKLAPRTDY | 50.1 | DMSO | Genscript |
| 346 | 19 | (K19D) | GLRKRLRKFRNKIKEKLKDIGQKIQGLLPKLAPRTDY | 41.3 | DMSO | Genscript |
| 347 | 19 | (K19E) | GLRKRLRKFRNKIKEKLKEIGQKIQGLLPKLAPRTDY | 46.3 | DMSO | Genscript |
| 348 | 19 | (K19F) | GLRKRLRKFRNKIKEKLKFIGQKIQGLLPKLAPRTDY | 39 | DMSO | Genscript |
| 349 | 19 | (K19G) | GLRKRLRKFRNKIKEKLKGIGQKIQGLLPKLAPRTDY | 30.1 | DMSO | Genscript |
| 350 | 19 | (K19H) | GLRKRLRKFRNKIKEKLKHIGQKIQGLLPKLAPRTDY | 59.3 | DMSO | Genscript |
| 351 | 19 | (K19I) | GLRKRLRKFRNKIKEKLKIIGQKIQGLLPKLAPRTDY | 58.6 | DMSO | Genscript |
| 352 | 19 | (K19L) | GLRKRLRKFRNKIKEKLKLIGQKIQGLLPKLAPRTDY | 38.6 | DMSO | Genscript |
| 353 | 19 | (K19M) | GLRKRLRKFRNKIKEKLKMIGQKIQGLLPKLAPRTDY | 47.2 | DMSO | Genscript |
| 354 | 19 | (K19N) | GLRKRLRKFRNKIKEKLKNIGQKIQGLLPKLAPRTDY | 37.6 | DMSO | Genscript |
| 355 | 19 | (K19P) | GLRKRLRKFRNKIKEKLKPIGQKIQGLLPKLAPRTDY | 58.8 | DMSO | Genscript |
| 356 | 19 | (K19Q) | GLRKRLRKFRNKIKEKLKQIGQKIQGLLPKLAPRTDY | 57 | DMSO | Genscript |
| 357 | 19 | (K19R) | GLRKRLRKFRNKIKEKLKRIGQKIQGLLPKLAPRTDY | 54.7 | DMSO | Genscript |
| 358 | 19 | (K19S) | GLRKRLRKFRNKIKEKLKSIGQKIQGLLPKLAPRTDY | 39.6 | DMSO | Genscript |
| 359 | 19 | (K19T) | GLRKRLRKFRNKIKEKLKTIGQKIQGLLPKLAPRTDY | 38.3 | DMSO | Genscript |
| 360 | 19 | (K19V) | GLRKRLRKFRNKIKEKLKVIGQKIQGLLPKLAPRTDY | 38.7 | DMSO | Genscript |
| 361 | 19 | (K19W) | GLRKRLRKFRNKIKEKLKWIGQKIQGLLPKLAPRTDY | 53.4 | DMSO | Genscript |
| 362 | 19 | (K19Y) | GLRKRLRKFRNKIKEKLKYIGQKIQGLLPKLAPRTDY | 65.9 | DMSO | Genscript |
| 363 | 20 | (I20A) | GLRKRLRKFRNKIKEKLKKAGQKIQGLLPKLAPRTDY | 57.1 | DMSO | Genscript |
| 364 | 20 | (I20C) | GLRKRLRKFRNKIKEKLKKCGQKIQGLLPKLAPRTDY | 43.7 | DMSO | Genscript |
| 365 | 20 | (I20D) | GLRKRLRKFRNKIKEKLKKDGQKIQGLLPKLAPRTDY | 52.1 | DMSO | Genscript |
| 366 | 20 | (I20E) | GLRKRLRKFRNKIKEKLKKEGQKIQGLLPKLAPRTDY | 55.9 | DMSO | Genscript |
| 367 | 20 | (I20F) | GLRKRLRKFRNKIKEKLKKFGQKIQGLLPKLAPRTDY | 51.7 | DMSO | Genscript |
| 368 | 20 | (I20G) | GLRKRLRKFRNKIKEKLKKGGQKIQGLLPKLAPRTDY | 67.9 | DMSO | Genscript |
| 369 | 20 | (I20H) | GLRKRLRKFRNKIKEKLKKHGQKIQGLLPKLAPRTDY | 50.9 | DMSO | Genscript |
| 370 | 20 | (I20K) | GLRKRLRKFRNKIKEKLKKKGQKIQGLLPKLAPRTDY | 48.1 | DMSO | Genscript |
| 371 | 20 | (I20L) | GLRKRLRKFRNKIKEKLKKLGQKIQGLLPKLAPRTDY | 55.6 | DMSO | Genscript |
| 372 | 20 | (I20M) | GLRKRLRKFRNKIKEKLKKMGQKIQGLLPKLAPRTDY | 35.8 | DMSO | Genscript |
| 373 | 20 | (I20N) | GLRKRLRKFRNKIKEKLKKNGQKIQGLLPKLAPRTDY | 43.1 | DMSO | Genscript |
| 374 | 20 | (I20P) | GLRKRLRKFRNKIKEKLKKPGQKIQGLLPKLAPRTDY | 71.9 | DMSO | Genscript |
| 375 | 20 | (I20Q) | GLRKRLRKFRNKIKEKLKKQGQKIQGLLPKLAPRTDY | 53 | DMSO | Genscript |
| 376 | 20 | (I20R) | GLRKRLRKFRNKIKEKLKKRGQKIQGLLPKLAPRTDY | 42.1 | DMSO | Genscript |
| 377 | 20 | (I20S) | GLRKRLRKFRNKIKEKLKKSGQKIQGLLPKLAPRTDY | 57.4 | DMSO | Genscript |
| 378 | 20 | (I20T) | GLRKRLRKFRNKIKEKLKKTGQKIQGLLPKLAPRTDY | 46.8 | DMSO | Genscript |
| 379 | 20 | (I20V) | GLRKRLRKFRNKIKEKLKKVGQKIQGLLPKLAPRTDY | 45.5 | DMSO | Genscript |
| 380 | 20 | (I20W) | GLRKRLRKFRNKIKEKLKKWGQKIQGLLPKLAPRTDY | 61.2 | DMSO | Genscript |
| 381 | 20 | (I20Y) | GLRKRLRKFRNKIKEKLKKYGQKIQGLLPKLAPRTDY | 65.6 | DMSO | Genscript |
| 382 | 21 | (G21A) | GLRKRLRKFRNKIKEKLKKIAQKIQGLLPKLAPRTDY | 48.9 | DMSO | Genscript |
| 383 | 21 | (G21C) | GLRKRLRKFRNKIKEKLKKICQKIQGLLPKLAPRTDY | 44.2 | DMSO | Genscript |
| 384 | 21 | (G21D) | GLRKRLRKFRNKIKEKLKKIDQKIQGLLPKLAPRTDY | 49.5 | DMSO | Genscript |
| 385 | 21 | (G21E) | GLRKRLRKFRNKIKEKLKKIEQKIQGLLPKLAPRTDY | 41.3 | DMSO | Genscript |
| 386 | 21 | (G21F) | GLRKRLRKFRNKIKEKLKKIFQKIQGLLPKLAPRTDY | 60.5 | DMSO | Genscript |
| 387 | 21 | (G21H) | GLRKRLRKFRNKIKEKLKKIHQKIQGLLPKLAPRTDY | 64.7 | DMSO | Genscript |
| 388 | 21 | (G21I) | GLRKRLRKFRNKIKEKLKKIIQKIQGLLPKLAPRTDY | 50.7 | DMSO | Genscript |
| 389 | 21 | (G21K) | GLRKRLRKFRNKIKEKLKKIKQKIQGLLPKLAPRTDY | 46.3 | DMSO | Genscript |
| 390 | 21 | (G21L) | GLRKRLRKFRNKIKEKLKKILQKIQGLLPKLAPRTDY | 42.7 | DMSO | Genscript |
| 391 | 21 | (G21M) | GLRKRLRKFRNKIKEKLKKIMQKIQGLLPKLAPRTDY | 47.5 | DMSO | Genscript |
| 392 | 21 | (G21N) | GLRKRLRKFRNKIKEKLKKINQKIQGLLPKLAPRTDY | 74.8 | DMSO | Genscript |
| 393 | 21 | (G21P) | GLRKRLRKFRNKIKEKLKKIPQKIQGLLPKLAPRTDY | 62.7 | DMSO | Genscript |
| 394 | 21 | (G21Q) | GLRKRLRKFRNKIKEKLKKIQQKIQGLLPKLAPRTDY | 46.7 | DMSO | Genscript |
| 395 | 21 | (G21R) | GLRKRLRKFRNKIKEKLKKIRQKIQGLLPKLAPRTDY | 38.6 | DMSO | Genscript |
| 396 | 21 | (G21S) | GLRKRLRKFRNKIKEKLKKISQKIQGLLPKLAPRTDY | 35.6 | DMSO | Genscript |
| 397 | 21 | (G21T) | GLRKRLRKFRNKIKEKLKKITQKIQGLLPKLAPRTDY | 19.3 | DMSO | Genscript |
| 398 | 21 | (G21V) | GLRKRLRKFRNKIKEKLKKIVQKIQGLLPKLAPRTDY | 59.2 | DMSO | Genscript |
| 399 | 21 | (G21W) | GLRKRLRKFRNKIKEKLKKIWQKIQGLLPKLAPRTDY | 56 | DMSO | Genscript |
| 400 | 21 | (G21Y) | GLRKRLRKFRNKIKEKLKKIYQKIQGLLPKLAPRTDY | 48.4 | DMSO | Genscript |
| 401 | 22 | (Q22A) | GLRKRLRKFRNKIKEKLKKIGAKIQGLLPKLAPRTDY | 48.4 | DMSO | Genscript |
| 402 | 22 | (Q22C) | GLRKRLRKFRNKIKEKLKKIGCKIQGLLPKLAPRTDY | 51.3 | DMSO | Genscript |
| 403 | 22 | (Q22D) | GLRKRLRKFRNKIKEKLKKIGDKIQGLLPKLAPRTDY | 50.9 | DMSO | Genscript |
| 404 | 22 | (Q22E) | GLRKRLRKFRNKIKEKLKKIGEKIQGLLPKLAPRTDY | 51.7 | DMSO | Genscript |
| 405 | 22 | (Q22F) | GLRKRLRKFRNKIKEKLKKIGFKIQGLLPKLAPRTDY | 43 | DMSO | Genscript |
| 406 | 22 | (Q22G) | GLRKRLRKFRNKIKEKLKKIGGKIQGLLPKLAPRTDY | 27.3 | DMSO | Genscript |
| 407 | 22 | (Q22H) | GLRKRLRKFRNKIKEKLKKIGHKIQGLLPKLAPRTDY | 51.1 | DMSO | Genscript |
| 408 | 22 | (Q22I) | GLRKRLRKFRNKIKEKLKKIGIKIQGLLPKLAPRTDY | 41.3 | DMSO | Genscript |
| 409 | 22 | (Q22K) | GLRKRLRKFRNKIKEKLKKIGKKIQGLLPKLAPRTDY | 34.8 | DMSO | Genscript |
| 410 | 22 | (Q22L) | GLRKRLRKFRNKIKEKLKKIGLKIQGLLPKLAPRTDY | 62.3 | DMSO | Genscript |
| 411 | 22 | (Q22M) | GLRKRLRKFRNKIKEKLKKIGMKIQGLLPKLAPRTDY | 45.4 | DMSO | Genscript |
| 412 | 22 | (Q22N) | GLRKRLRKFRNKIKEKLKKIGNKIQGLLPKLAPRTDY | 46 | DMSO | Genscript |
| 413 | 22 | (Q22P) | GLRKRLRKFRNKIKEKLKKIGPKIQGLLPKLAPRTDY | 62.1 | DMSO | Genscript |
| 414 | 22 | (Q22R) | GLRKRLRKFRNKIKEKLKKIGRKIQGLLPKLAPRTDY | 43.9 | DMSO | Genscript |
| 415 | 22 | (Q22S) | GLRKRLRKFRNKIKEKLKKIGSKIQGLLPKLAPRTDY | 19.3 | DMSO | Genscript |
| 416 | 22 | (Q22T) | GLRKRLRKFRNKIKEKLKKIGTKIQGLLPKLAPRTDY | 30.7 | DMSO | Genscript |
| 417 | 22 | (Q22V) | GLRKRLRKFRNKIKEKLKKIGVKIQGLLPKLAPRTDY | 37.4 | DMSO | Genscript |
| 418 | 22 | (Q22W) | GLRKRLRKFRNKIKEKLKKIGWKIQGLLPKLAPRTDY | 43.6 | DMSO | Genscript |
| 419 | 22 | (Q22Y) | GLRKRLRKFRNKIKEKLKKIGYKIQGLLPKLAPRTDY | 45.8 | DMSO | Genscript |
| 420 | 23 | (K23A) | GLRKRLRKFRNKIKEKLKKIGQAIQGLLPKLAPRTDY | 49.4 | DMSO | Genscript |
| 421 | 23 | (K23C) | GLRKRLRKFRNKIKEKLKKIGQCIQGLLPKLAPRTDY | 36.9 | DMSO | Genscript |
| 422 | 23 | (K23D) | GLRKRLRKFRNKIKEKLKKIGQDIQGLLPKLAPRTDY | 44.2 | DMSO | Genscript |
| 423 | 23 | (K23E) | GLRKRLRKFRNKIKEKLKKIGQEIQGLLPKLAPRTDY | 43.1 | DMSO | Genscript |
| 424 | 23 | (K23F) | GLRKRLRKFRNKIKEKLKKIGQFIQGLLPKLAPRTDY | 45.4 | DMSO | Genscript |
| 425 | 23 | (K23G) | GLRKRLRKFRNKIKEKLKKIGQGIQGLLPKLAPRTDY | 39.5 | DMSO | Genscript |
| 426 | 23 | (K23H) | GLRKRLRKFRNKIKEKLKKIGQHIQGLLPKLAPRTDY | 41.2 | DMSO | Genscript |
| 427 | 23 | (K23I) | GLRKRLRKFRNKIKEKLKKIGQIIQGLLPKLAPRTDY | 38.9 | DMSO | Genscript |
| 428 | 23 | (K23L) | GLRKRLRKFRNKIKEKLKKIGQLIQGLLPKLAPRTDY | 40.1 | DMSO | Genscript |
| 429 | 23 | (K23M) | GLRKRLRKFRNKIKEKLKKIGQMIQGLLPKLAPRTDY | 45.2 | DMSO | Genscript |
| 430 | 23 | (K23N) | GLRKRLRKFRNKIKEKLKKIGQNIQGLLPKLAPRTDY | 32.6 | DMSO | Genscript |
| 431 | 23 | (K23P) | GLRKRLRKFRNKIKEKLKKIGQPIQGLLPKLAPRTDY | 48.9 | DMSO | Genscript |
| 432 | 23 | (K23Q) | GLRKRLRKFRNKIKEKLKKIGQQIQGLLPKLAPRTDY | 50.3 | DMSO | Genscript |
| 433 | 23 | (K23R) | GLRKRLRKFRNKIKEKLKKIGQRIQGLLPKLAPRTDY | 72.4 | DMSO | Genscript |
| 434 | 23 | (K23S) | GLRKRLRKFRNKIKEKLKKIGQSIQGLLPKLAPRTDY | 36.4 | DMSO | Genscript |
| 435 | 23 | (K23T) | GLRKRLRKFRNKIKEKLKKIGQTIQGLLPKLAPRTDY | 26.9 | DMSO | Genscript |
| 436 | 23 | (K23V) | GLRKRLRKFRNKIKEKLKKIGQVIQGLLPKLAPRTDY | 45.8 | DMSO | Genscript |
| 437 | 23 | (K23W) | GLRKRLRKFRNKIKEKLKKIGQWIQGLLPKLAPRTDY | 46.2 | DMSO | Genscript |
| 438 | 23 | (K23Y) | GLRKRLRKFRNKIKEKLKKIGQYIQGLLPKLAPRTDY | 51.8 | DMSO | Genscript |
| 439 | 24 | (I24A) | GLRKRLRKFRNKIKEKLKKIGQKAQGLLPKLAPRTDY | 61.5 | DMSO | Genscript |
| 440 | 24 | (I24C) | GLRKRLRKFRNKIKEKLKKIGQKCQGLLPKLAPRTDY | 43.9 | DMSO | Genscript |
| 441 | 24 | (I24D) | GLRKRLRKFRNKIKEKLKKIGQKDQGLLPKLAPRTDY | 49.7 | DMSO | Genscript |
| 442 | 24 | (I24E) | GLRKRLRKFRNKIKEKLKKIGQKEQGLLPKLAPRTDY | 46.8 | DMSO | Genscript |
| 443 | 24 | (I24F) | GLRKRLRKFRNKIKEKLKKIGQKFQGLLPKLAPRTDY | 55.2 | DMSO | Genscript |
| 444 | 24 | (I24G) | GLRKRLRKFRNKIKEKLKKIGQKGQGLLPKLAPRTDY | 38.8 | DMSO | Genscript |
| 445 | 24 | (I24H) | GLRKRLRKFRNKIKEKLKKIGQKHQGLLPKLAPRTDY | 71.3 | DMSO | Genscript |
| 446 | 24 | (I24K) | GLRKRLRKFRNKIKEKLKKIGQKKQGLLPKLAPRTDY | 27.6 | DMSO | Genscript |
| 447 | 24 | (I24L) | GLRKRLRKFRNKIKEKLKKIGQKLQGLLPKLAPRTDY | 55.6 | DMSO | Genscript |
| 448 | 24 | (I24M) | GLRKRLRKFRNKIKEKLKKIGQKMQGLLPKLAPRTDY | 49 | DMSO | Genscript |
| 449 | 24 | (I24N) | GLRKRLRKFRNKIKEKLKKIGQKNQGLLPKLAPRTDY | 48.5 | DMSO | Genscript |
| 450 | 24 | (I24P) | GLRKRLRKFRNKIKEKLKKIGQKPQGLLPKLAPRTDY | 37 | DMSO | Genscript |
| 451 | 24 | (I24Q) | GLRKRLRKFRNKIKEKLKKIGQKQQGLLPKLAPRTDY | 81 | DMSO | Genscript |
| 452 | 24 | (I24R) | GLRKRLRKFRNKIKEKLKKIGQKRQGLLPKLAPRTDY | 37.8 | DMSO | Genscript |
| 453 | 24 | (I24S) | GLRKRLRKFRNKIKEKLKKIGQKSQGLLPKLAPRTDY | 55.9 | DMSO | Genscript |
| 454 | 24 | (I24T) | GLRKRLRKFRNKIKEKLKKIGQKTQGLLPKLAPRTDY | 38.3 | DMSO | Genscript |
| 455 | 24 | (I24V) | GLRKRLRKFRNKIKEKLKKIGQKVQGLLPKLAPRTDY | 48.6 | DMSO | Genscript |
| 456 | 24 | (I24W) | GLRKRLRKFRNKIKEKLKKIGQKWQGLLPKLAPRTDY | 48.8 | DMSO | Genscript |
| 457 | 24 | (I24Y) | GLRKRLRKFRNKIKEKLKKIGQKYQGLLPKLAPRTDY | 52.3 | DMSO | Genscript |
| 458 | 25 | (Q25A) | GLRKRLRKFRNKIKEKLKKIGQKIAGLLPKLAPRTDY | 40.5 | DMSO | Genscript |
| 459 | 25 | (Q25C) | GLRKRLRKFRNKIKEKLKKIGQKICGLLPKLAPRTDY | 35.9 | DMSO | Genscript |
| 460 | 25 | (Q25D) | GLRKRLRKFRNKIKEKLKKIGQKIDGLLPKLAPRTDY | 49.1 | DMSO | Genscript |
| 461 | 25 | (Q25E) | GLRKRLRKFRNKIKEKLKKIGQKIEGLLPKLAPRTDY | 40.6 | DMSO | Genscript |
| 462 | 25 | (Q25F) | GLRKRLRKFRNKIKEKLKKIGQKIFGLLPKLAPRTDY | 33.6 | DMSO | Genscript |
| 463 | 25 | (Q25G) | GLRKRLRKFRNKIKEKLKKIGQKIGGLLPKLAPRTDY | 47.2 | DMSO | Genscript |
| 464 | 25 | (Q25H) | GLRKRLRKFRNKIKEKLKKIGQKIHGLLPKLAPRTDY | 48.2 | DMSO | Genscript |
| 465 | 25 | (Q25I) | GLRKRLRKFRNKIKEKLKKIGQKIIGLLPKLAPRTDY | 38.2 | DMSO | Genscript |
| 466 | 25 | (Q25K) | GLRKRLRKFRNKIKEKLKKIGQKIKGLLPKLAPRTDY | 53.3 | DMSO | Genscript |
| 467 | 25 | (Q25L) | GLRKRLRKFRNKIKEKLKKIGQKILGLLPKLAPRTDY | 38.7 | DMSO | Genscript |
| 468 | 25 | (Q25M) | GLRKRLRKFRNKIKEKLKKIGQKIMGLLPKLAPRTDY | 46.4 | DMSO | Genscript |
| 469 | 25 | (Q25N) | GLRKRLRKFRNKIKEKLKKIGQKINGLLPKLAPRTDY | 48.7 | DMSO | Genscript |
| 470 | 25 | (Q25P) | GLRKRLRKFRNKIKEKLKKIGQKIPGLLPKLAPRTDY | 46.3 | DMSO | Genscript |
| 471 | 25 | (Q25R) | GLRKRLRKFRNKIKEKLKKIGQKIRGLLPKLAPRTDY | 58.3 | DMSO | Genscript |
| 472 | 25 | (Q25S) | GLRKRLRKFRNKIKEKLKKIGQKISGLLPKLAPRTDY | 42.9 | DMSO | Genscript |
| 473 | 25 | (Q25T) | GLRKRLRKFRNKIKEKLKKIGQKITGLLPKLAPRTDY | 23.2 | DMSO | Genscript |
| 474 | 25 | (Q25V) | GLRKRLRKFRNKIKEKLKKIGQKIVGLLPKLAPRTDY | 49.6 | DMSO | Genscript |
| 475 | 25 | (Q25W) | GLRKRLRKFRNKIKEKLKKIGQKIWGLLPKLAPRTDY | 61.8 | DMSO | Genscript |
| 476 | 25 | (Q25Y) | GLRKRLRKFRNKIKEKLKKIGQKIYGLLPKLAPRTDY | 39 | DMSO | Genscript |
| 477 | 26 | (G26A) | GLRKRLRKFRNKIKEKLKKIGQKIQALLPKLAPRTDY | 44.1 | DMSO | Genscript |
| 478 | 26 | (G26C) | GLRKRLRKFRNKIKEKLKKIGQKIQCLLPKLAPRTDY | 46.7 | DMSO | Genscript |
| 479 | 26 | (G26D) | GLRKRLRKFRNKIKEKLKKIGQKIQDLLPKLAPRTDY | 52 | DMSO | Genscript |
| 480 | 26 | (G26E) | GLRKRLRKFRNKIKEKLKKIGQKIQELLPKLAPRTDY | 31.3 | DMSO | Genscript |
| 481 | 26 | (G26F) | GLRKRLRKFRNKIKEKLKKIGQKIQFLLPKLAPRTDY | 60.8 | DMSO | Genscript |
| 482 | 26 | (G26H) | GLRKRLRKFRNKIKEKLKKIGQKIQHLLPKLAPRTDY | 44.2 | DMSO | Genscript |
| 483 | 26 | (G26I) | GLRKRLRKFRNKIKEKLKKIGQKIQILLPKLAPRTDY | 43.5 | DMSO | Genscript |
| 484 | 26 | (G26K) | GLRKRLRKFRNKIKEKLKKIGQKIQKLLPKLAPRTDY | 58.6 | DMSO | Genscript |
| 485 | 26 | (G26L) | GLRKRLRKFRNKIKEKLKKIGQKIQLLLPKLAPRTDY | 30.6 | DMSO | Genscript |
| 486 | 26 | (G26M) | GLRKRLRKFRNKIKEKLKKIGQKIQMLLPKLAPRTDY | 56.5 | DMSO | Genscript |
| 487 | 26 | (G26N) | GLRKRLRKFRNKIKEKLKKIGQKIQNLLPKLAPRTDY | 63.2 | DMSO | Genscript |
| 488 | 26 | (G26P) | GLRKRLRKFRNKIKEKLKKIGQKIQPLLPKLAPRTDY | 42.3 | DMSO | Genscript |
| 489 | 26 | (G26Q) | GLRKRLRKFRNKIKEKLKKIGQKIQQLLPKLAPRTDY | 60.8 | DMSO | Genscript |
| 490 | 26 | (G26R) | GLRKRLRKFRNKIKEKLKKIGQKIQRLLPKLAPRTDY | 50.1 | DMSO | Genscript |
| 491 | 26 | (G26S) | GLRKRLRKFRNKIKEKLKKIGQKIQSLLPKLAPRTDY | 25.7 | DMSO | Genscript |
| 492 | 26 | (G26T) | GLRKRLRKFRNKIKEKLKKIGQKIQTLLPKLAPRTDY | 47.8 | DMSO | Genscript |
| 493 | 26 | (G26V) | GLRKRLRKFRNKIKEKLKKIGQKIQVLLPKLAPRTDY | 59.8 | DMSO | Genscript |
| 494 | 26 | (G26W) | GLRKRLRKFRNKIKEKLKKIGQKIQWLLPKLAPRTDY | 46.3 | DMSO | Genscript |
| 495 | 26 | (G26Y) | GLRKRLRKFRNKIKEKLKKIGQKIQYLLPKLAPRTDY | 46.3 | DMSO | Genscript |
| 496 | 27 | (L27A) | GLRKRLRKFRNKIKEKLKKIGQKIQGALPKLAPRTDY | 43.7 | DMSO | Genscript |
| 497 | 27 | (L27C) | GLRKRLRKFRNKIKEKLKKIGQKIQGCLPKLAPRTDY | 22.8 | DMSO | Genscript |
| 498 | 27 | (L27D) | GLRKRLRKFRNKIKEKLKKIGQKIQGDLPKLAPRTDY | 36 | DMSO | Genscript |
| 499 | 27 | (L27E) | GLRKRLRKFRNKIKEKLKKIGQKIQGELPKLAPRTDY | 52.7 | DMSO | Genscript |
| 500 | 27 | (L27F) | GLRKRLRKFRNKIKEKLKKIGQKIQGFLPKLAPRTDY | 46.3 | DMSO | Genscript |
| 501 | 27 | (L27G) | GLRKRLRKFRNKIKEKLKKIGQKIQGGLPKLAPRTDY | 43.7 | DMSO | Genscript |
| 502 | 27 | (L27H) | GLRKRLRKFRNKIKEKLKKIGQKIQGHLPKLAPRTDY | 53.4 | DMSO | Genscript |
| 503 | 27 | (L27I) | GLRKRLRKFRNKIKEKLKKIGQKIQGILPKLAPRTDY | 34.5 | DMSO | Genscript |
| 504 | 27 | (L27K) | GLRKRLRKFRNKIKEKLKKIGQKIQGKLPKLAPRTDY | 42.5 | DMSO | Genscript |
| 505 | 27 | (L27M) | GLRKRLRKFRNKIKEKLKKIGQKIQGMLPKLAPRTDY | 35.4 | DMSO | Genscript |
| 506 | 27 | (L27N) | GLRKRLRKFRNKIKEKLKKIGQKIQGNLPKLAPRTDY | 46.7 | DMSO | Genscript |
| 507 | 27 | (L27P) | GLRKRLRKFRNKIKEKLKKIGQKIQGPLPKLAPRTDY | 39.3 | DMSO | Genscript |
| 508 | 27 | (L27Q) | GLRKRLRKFRNKIKEKLKKIGQKIQGQLPKLAPRTDY | 53.6 | DMSO | Genscript |
| 509 | 27 | (L27R) | GLRKRLRKFRNKIKEKLKKIGQKIQGRLPKLAPRTDY | 28.9 | DMSO | Genscript |
| 510 | 27 | (L27S) | GLRKRLRKFRNKIKEKLKKIGQKIQGSLPKLAPRTDY | 40.2 | DMSO | Genscript |
| 511 | 27 | (L27T) | GLRKRLRKFRNKIKEKLKKIGQKIQGTLPKLAPRTDY | 41.6 | DMSO | Genscript |
| 512 | 27 | (L27V) | GLRKRLRKFRNKIKEKLKKIGQKIQGVLPKLAPRTDY | 39.2 | DMSO | Genscript |
| 513 | 27 | (L27W) | GLRKRLRKFRNKIKEKLKKIGQKIQGWLPKLAPRTDY | 54.7 | DMSO | Genscript |
| 514 | 27 | (L27Y) | GLRKRLRKFRNKIKEKLKKIGQKIQGYLPKLAPRTDY | 55.3 | DMSO | Genscript |
| 515 | 28 | (L28A) | GLRKRLRKFRNKIKEKLKKIGQKIQGLAPKLAPRTDY | 52.3 | DMSO | Genscript |
| 516 | 28 | (L28A) | GLRKRLRKFRNKIKEKLKKIGQKIQGLCPKLAPRTDY | 48 | DMSO | Genscript |
| 517 | 28 | (L28A) | GLRKRLRKFRNKIKEKLKKIGQKIQGLDPKLAPRTDY | 37.4 | DMSO | Genscript |
| 518 | 28 | (L28A) | GLRKRLRKFRNKIKEKLKKIGQKIQGLEPKLAPRTDY | 41.4 | DMSO | Genscript |
| 519 | 28 | (L28A) | GLRKRLRKFRNKIKEKLKKIGQKIQGLFPKLAPRTDY | 42.7 | DMSO | Genscript |
| 520 | 28 | (L28A) | GLRKRLRKFRNKIKEKLKKIGQKIQGLGPKLAPRTDY | 56.3 | DMSO | Genscript |
| 521 | 28 | (L28A) | GLRKRLRKFRNKIKEKLKKIGQKIQGLHPKLAPRTDY | 33.3 | DMSO | Genscript |
| 522 | 28 | (L28A) | GLRKRLRKFRNKIKEKLKKIGQKIQGLIPKLAPRTDY | 55.3 | DMSO | Genscript |
| 523 | 28 | (L28A) | GLRKRLRKFRNKIKEKLKKIGQKIQGLKPKLAPRTDY | 79.3 | DMSO | Genscript |
| 524 | 28 | (L28A) | GLRKRLRKFRNKIKEKLKKIGQKIQGLMPKLAPRTDY | 21.9 | DMSO | Genscript |
| 525 | 28 | (L28A) | GLRKRLRKFRNKIKEKLKKIGQKIQGLNPKLAPRTDY | 44.5 | DMSO | Genscript |
| 526 | 28 | (L28P) | GLRKRLRKFRNKIKEKLKKIGQKIQGLPPKLAPRTDY | 48.2 | DMSO | Genscript |
| 527 | 28 | (L28Q) | GLRKRLRKFRNKIKEKLKKIGQKIQGLQPKLAPRTDY | 64.8 | DMSO | Genscript |
| 528 | 28 | (L28R) | GLRKRLRKFRNKIKEKLKKIGQKIQGLRPKLAPRTDY | 53.8 | DMSO | Genscript |
| 529 | 28 | (L28S) | GLRKRLRKFRNKIKEKLKKIGQKIQGLSPKLAPRTDY | 36.4 | DMSO | Genscript |
| 530 | 28 | (L28T) | GLRKRLRKFRNKIKEKLKKIGQKIQGLTPKLAPRTDY | 49.5 | DMSO | Genscript |
| 531 | 28 | (L28V) | GLRKRLRKFRNKIKEKLKKIGQKIQGLVPKLAPRTDY | 46.8 | DMSO | Genscript |
| 532 | 28 | (L28W) | GLRKRLRKFRNKIKEKLKKIGQKIQGLWPKLAPRTDY | 52.4 | DMSO | Genscript |
| 533 | 28 | (L28Y) | GLRKRLRKFRNKIKEKLKKIGQKIQGLYPKLAPRTDY | 38 | DMSO | Genscript |
| 534 | 29 | (P29A) | GLRKRLRKFRNKIKEKLKKIGQKIQGLLAKLAPRTDY | 48.2 | DMSO | Genscript |
| 535 | 29 | (P29C) | GLRKRLRKFRNKIKEKLKKIGQKIQGLLCKLAPRTDY | 27.5 | DMSO | Genscript |
| 536 | 29 | (P29D) | GLRKRLRKFRNKIKEKLKKIGQKIQGLLDKLAPRTDY | 20.2 | DMSO | Genscript |
| 537 | 29 | (P29E) | GLRKRLRKFRNKIKEKLKKIGQKIQGLLEKLAPRTDY | 48.9 | DMSO | Genscript |
| 538 | 29 | (P29F) | GLRKRLRKFRNKIKEKLKKIGQKIQGLLFKLAPRTDY | 42.2 | DMSO | Genscript |
| 539 | 29 | (P29G) | GLRKRLRKFRNKIKEKLKKIGQKIQGLLGKLAPRTDY | 54.4 | DMSO | Genscript |
| 540 | 29 | (P29H) | GLRKRLRKFRNKIKEKLKKIGQKIQGLLHKLAPRTDY | 56.7 | DMSO | Genscript |
| 541 | 29 | (P29I) | GLRKRLRKFRNKIKEKLKKIGQKIQGLLIKLAPRTDY | 47.5 | DMSO | Genscript |
| 542 | 29 | (P29K) | GLRKRLRKFRNKIKEKLKKIGQKIQGLLKKLAPRTDY | 60.7 | DMSO | Genscript |
| 543 | 29 | (P29L) | GLRKRLRKFRNKIKEKLKKIGQKIQGLLLKLAPRTDY | 53.2 | DMSO | Genscript |
| 544 | 29 | (P29M) | GLRKRLRKFRNKIKEKLKKIGQKIQGLLMKLAPRTDY | 38.1 | DMSO | Genscript |
| 545 | 29 | (P29N) | GLRKRLRKFRNKIKEKLKKIGQKIQGLLNKLAPRTDY | 59.4 | DMSO | Genscript |
| 546 | 29 | (P29Q) | GLRKRLRKFRNKIKEKLKKIGQKIQGLLQKLAPRTDY | 57.3 | DMSO | Genscript |
| 547 | 29 | (P29R) | GLRKRLRKFRNKIKEKLKKIGQKIQGLLRKLAPRTDY | 62.9 | DMSO | Genscript |
| 548 | 29 | (P29S) | GLRKRLRKFRNKIKEKLKKIGQKIQGLLSKLAPRTDY | 52.8 | DMSO | Genscript |
| 549 | 29 | (P29T) | GLRKRLRKFRNKIKEKLKKIGQKIQGLLTKLAPRTDY | 19.6 | DMSO | Genscript |
| 550 | 29 | (P29V) | GLRKRLRKFRNKIKEKLKKIGQKIQGLLVKLAPRTDY | 38.8 | DMSO | Genscript |
| 551 | 29 | (P29W) | GLRKRLRKFRNKIKEKLKKIGQKIQGLLWKLAPRTDY | 54.8 | DMSO | Genscript |
| 552 | 29 | (P29Y) | GLRKRLRKFRNKIKEKLKKIGQKIQGLLYKLAPRTDY | 53.8 | DMSO | Genscript |
| 553 | 30 | (K30A) | GLRKRLRKFRNKIKEKLKKIGQKIQGLLPALAPRTDY | 62.9 | DMSO | Genscript |
| 554 | 30 | (K30C) | GLRKRLRKFRNKIKEKLKKIGQKIQGLLPCLAPRTDY | 50 | DMSO | Genscript |
| 555 | 30 | (K30D) | GLRKRLRKFRNKIKEKLKKIGQKIQGLLPDLAPRTDY | 51.8 | DMSO | Genscript |
| 556 | 30 | (K30E) | GLRKRLRKFRNKIKEKLKKIGQKIQGLLPELAPRTDY | 50.9 | DMSO | Genscript |
| 557 | 30 | (K30F) | GLRKRLRKFRNKIKEKLKKIGQKIQGLLPFLAPRTDY | 62.8 | DMSO | Genscript |
| 558 | 30 | (K30G) | GLRKRLRKFRNKIKEKLKKIGQKIQGLLPGLAPRTDY | 56.8 | DMSO | Genscript |
| 559 | 30 | (K30H) | GLRKRLRKFRNKIKEKLKKIGQKIQGLLPHLAPRTDY | 64.7 | DMSO | Genscript |
| 560 | 30 | (K30I) | GLRKRLRKFRNKIKEKLKKIGQKIQGLLPILAPRTDY | 60.1 | DMSO | Genscript |
| 561 | 30 | (K30L) | GLRKRLRKFRNKIKEKLKKIGQKIQGLLPLLAPRTDY | 63.8 | DMSO | Genscript |
| 562 | 30 | (K30M) | GLRKRLRKFRNKIKEKLKKIGQKIQGLLPMLAPRTDY | 43.1 | DMSO | Genscript |
| 563 | 30 | (K30N) | GLRKRLRKFRNKIKEKLKKIGQKIQGLLPNLAPRTDY | 32 | DMSO | Genscript |
| 564 | 30 | (K30P) | GLRKRLRKFRNKIKEKLKKIGQKIQGLLPPLAPRTDY | 50.3 | DMSO | Genscript |
| 565 | 30 | (K30Q) | GLRKRLRKFRNKIKEKLKKIGQKIQGLLPQLAPRTDY | 31 | DMSO | Genscript |
| 566 | 30 | (K30R) | GLRKRLRKFRNKIKEKLKKIGQKIQGLLPRLAPRTDY | 69.7 | DMSO | Genscript |
| 567 | 30 | (K30S) | GLRKRLRKFRNKIKEKLKKIGQKIQGLLPSLAPRTDY | 57.8 | DMSO | Genscript |
| 568 | 30 | (K30T) | GLRKRLRKFRNKIKEKLKKIGQKIQGLLPTLAPRTDY | 53 | DMSO | Genscript |
| 569 | 30 | (K30V) | GLRKRLRKFRNKIKEKLKKIGQKIQGLLPVLAPRTDY | 62.8 | DMSO | Genscript |
| 570 | 30 | (K30W) | GLRKRLRKFRNKIKEKLKKIGQKIQGLLPWLAPRTDY | 59.3 | DMSO | Genscript |
| 571 | 30 | (K30Y) | GLRKRLRKFRNKIKEKLKKIGQKIQGLLPYLAPRTDY | 38.4 | DMSO | Genscript |
| 572 | 31 | (L31A) | GLRKRLRKFRNKIKEKLKKIGQKIQGLLPKAAPRTDY | 60.1 | DMSO | Genscript |
| 573 | 31 | (L31C) | GLRKRLRKFRNKIKEKLKKIGQKIQGLLPKCAPRTDY | 47.2 | DMSO | Genscript |
| 574 | 31 | (L31D) | GLRKRLRKFRNKIKEKLKKIGQKIQGLLPKDAPRTDY | 58 | DMSO | Genscript |
| 575 | 31 | (L31E) | GLRKRLRKFRNKIKEKLKKIGQKIQGLLPKEAPRTDY | 45.6 | DMSO | Genscript |
| 576 | 31 | (L31F) | GLRKRLRKFRNKIKEKLKKIGQKIQGLLPKFAPRTDY | 40.1 | DMSO | Genscript |
| 577 | 31 | (L31G) | GLRKRLRKFRNKIKEKLKKIGQKIQGLLPKGAPRTDY | 44.3 | DMSO | Genscript |
| 578 | 31 | (L31H) | GLRKRLRKFRNKIKEKLKKIGQKIQGLLPKHAPRTDY | 62.5 | DMSO | Genscript |
| 579 | 31 | (L31I) | GLRKRLRKFRNKIKEKLKKIGQKIQGLLPKIAPRTDY | 59.7 | DMSO | Genscript |
| 580 | 31 | (L31K) | GLRKRLRKFRNKIKEKLKKIGQKIQGLLPKKAPRTDY | 63.6 | DMSO | Genscript |
| 581 | 31 | (L31M) | GLRKRLRKFRNKIKEKLKKIGQKIQGLLPKMAPRTDY | 46.4 | DMSO | Genscript |
| 582 | 31 | (L31N) | GLRKRLRKFRNKIKEKLKKIGQKIQGLLPKNAPRTDY | 55.4 | DMSO | Genscript |
| 583 | 31 | (L31P) | GLRKRLRKFRNKIKEKLKKIGQKIQGLLPKPAPRTDY | 45 | DMSO | Genscript |
| 584 | 31 | (L31Q) | GLRKRLRKFRNKIKEKLKKIGQKIQGLLPKQAPRTDY | 64.2 | DMSO | Genscript |
| 585 | 31 | (L31R) | GLRKRLRKFRNKIKEKLKKIGQKIQGLLPKRAPRTDY | 36.6 | DMSO | Genscript |
| 586 | 31 | (L31S) | GLRKRLRKFRNKIKEKLKKIGQKIQGLLPKSAPRTDY | 61.4 | DMSO | Genscript |
| 587 | 31 | (L31T) | GLRKRLRKFRNKIKEKLKKIGQKIQGLLPKTAPRTDY | 61.7 | DMSO | Genscript |
| 588 | 31 | (L31V) | GLRKRLRKFRNKIKEKLKKIGQKIQGLLPKVAPRTDY | 49 | DMSO | Genscript |
| 589 | 31 | (L31W) | GLRKRLRKFRNKIKEKLKKIGQKIQGLLPKWAPRTDY | 47.1 | DMSO | Genscript |
| 590 | 31 | (L31Y) | GLRKRLRKFRNKIKEKLKKIGQKIQGLLPKYAPRTDY | 48.1 | DMSO | Genscript |
| 591 | 32 | (A32C) | GLRKRLRKFRNKIKEKLKKIGQKIQGLLPKLCPRTDY | 41.5 | DMSO | Genscript |
| 592 | 32 | (A32D) | GLRKRLRKFRNKIKEKLKKIGQKIQGLLPKLDPRTDY | 59.2 | DMSO | Genscript |
| 593 | 32 | (A32E) | GLRKRLRKFRNKIKEKLKKIGQKIQGLLPKLEPRTDY | 60.8 | DMSO | Genscript |
| 594 | 32 | (A32F) | GLRKRLRKFRNKIKEKLKKIGQKIQGLLPKLFPRTDY | 55.6 | DMSO | Genscript |
| 595 | 32 | (A32G) | GLRKRLRKFRNKIKEKLKKIGQKIQGLLPKLGPRTDY | 42 | DMSO | Genscript |
| 596 | 32 | (A32H) | GLRKRLRKFRNKIKEKLKKIGQKIQGLLPKLHPRTDY | 46.8 | DMSO | Genscript |
| 597 | 32 | (A32I) | GLRKRLRKFRNKIKEKLKKIGQKIQGLLPKLIPRTDY | 41 | DMSO | Genscript |
| 598 | 32 | (A32K) | GLRKRLRKFRNKIKEKLKKIGQKIQGLLPKLKPRTDY | 67.7 | DMSO | Genscript |
| 599 | 32 | (A32L) | GLRKRLRKFRNKIKEKLKKIGQKIQGLLPKLLPRTDY | 63.3 | DMSO | Genscript |
| 600 | 32 | (A32M) | GLRKRLRKFRNKIKEKLKKIGQKIQGLLPKLMPRTDY | 53.5 | DMSO | Genscript |
| 601 | 32 | (A32N) | GLRKRLRKFRNKIKEKLKKIGQKIQGLLPKLNPRTDY | 44.1 | DMSO | Genscript |
| 602 | 32 | (A32P) | GLRKRLRKFRNKIKEKLKKIGQKIQGLLPKLPPRTDY | 47.9 | DMSO | Genscript |
| 603 | 32 | (A32Q) | GLRKRLRKFRNKIKEKLKKIGQKIQGLLPKLQPRTDY | 45.6 | DMSO | Genscript |
| 604 | 32 | (A32R) | GLRKRLRKFRNKIKEKLKKIGQKIQGLLPKLRPRTDY | 62.4 | DMSO | Genscript |
| 605 | 32 | (A32S) | GLRKRLRKFRNKIKEKLKKIGQKIQGLLPKLSPRTDY | 70.9 | DMSO | Genscript |
| 606 | 32 | (A32T) | GLRKRLRKFRNKIKEKLKKIGQKIQGLLPKLTPRTDY | 48.1 | DMSO | Genscript |
| 607 | 32 | (A32V) | GLRKRLRKFRNKIKEKLKKIGQKIQGLLPKLVPRTDY | 49 | DMSO | Genscript |
| 608 | 32 | (A32W) | GLRKRLRKFRNKIKEKLKKIGQKIQGLLPKLWPRTDY | 53.4 | DMSO | Genscript |
| 609 | 32 | (A32Y) | GLRKRLRKFRNKIKEKLKKIGQKIQGLLPKLYPRTDY | 61.6 | DMSO | Genscript |
| 610 | 33 | (P33A) | GLRKRLRKFRNKIKEKLKKIGQKIQGLLPKLAARTDY | 53.2 | DMSO | Genscript |
| 611 | 33 | (P33C) | GLRKRLRKFRNKIKEKLKKIGQKIQGLLPKLACRTDY | 45 | DMSO | Genscript |
| 612 | 33 | (P33D) | GLRKRLRKFRNKIKEKLKKIGQKIQGLLPKLADRTDY | 57.4 | DMSO | Genscript |
| 613 | 33 | (P33E) | GLRKRLRKFRNKIKEKLKKIGQKIQGLLPKLAERTDY | 64.7 | DMSO | Genscript |
| 614 | 33 | (P33F) | GLRKRLRKFRNKIKEKLKKIGQKIQGLLPKLAFRTDY | 44.7 | DMSO | Genscript |
| 615 | 33 | (P33G) | GLRKRLRKFRNKIKEKLKKIGQKIQGLLPKLAGRTDY | 68.7 | DMSO | Genscript |
| 616 | 33 | (P33H) | GLRKRLRKFRNKIKEKLKKIGQKIQGLLPKLAHRTDY | 54.2 | DMSO | Genscript |
| 617 | 33 | (P33I) | GLRKRLRKFRNKIKEKLKKIGQKIQGLLPKLAIRTDY | 54 | DMSO | Genscript |
| 618 | 33 | (P33K) | GLRKRLRKFRNKIKEKLKKIGQKIQGLLPKLAKRTDY | 64.9 | DMSO | Genscript |
| 619 | 33 | (P33L) | GLRKRLRKFRNKIKEKLKKIGQKIQGLLPKLALRTDY | 47.4 | DMSO | Genscript |
| 620 | 33 | (P33M) | GLRKRLRKFRNKIKEKLKKIGQKIQGLLPKLAMRTDY | 62.7 | DMSO | Genscript |
| 621 | 33 | (P33N) | GLRKRLRKFRNKIKEKLKKIGQKIQGLLPKLANRTDY | 73.3 | DMSO | Genscript |
| 622 | 33 | (P33Q) | GLRKRLRKFRNKIKEKLKKIGQKIQGLLPKLAQRTDY | 66.6 | DMSO | Genscript |
| 623 | 33 | (P33R) | GLRKRLRKFRNKIKEKLKKIGQKIQGLLPKLARRTDY | 71.6 | DMSO | Genscript |
| 624 | 33 | (P33S) | GLRKRLRKFRNKIKEKLKKIGQKIQGLLPKLASRTDY | 55 | DMSO | Genscript |
| 625 | 33 | (P33T) | GLRKRLRKFRNKIKEKLKKIGQKIQGLLPKLATRTDY | 34.3 | DMSO | Genscript |
| 626 | 33 | (P33V) | GLRKRLRKFRNKIKEKLKKIGQKIQGLLPKLAVRTDY | 46.5 | DMSO | Genscript |
| 627 | 33 | (P33W) | GLRKRLRKFRNKIKEKLKKIGQKIQGLLPKLAWRTDY | 42.8 | DMSO | Genscript |
| 628 | 33 | (P33Y) | GLRKRLRKFRNKIKEKLKKIGQKIQGLLPKLAYRTDY | 68.2 | DMSO | Genscript |
| 629 | 34 | (R34A) | GLRKRLRKFRNKIKEKLKKIGQKIQGLLPKLAPATDY | 69.6 | DMSO | Genscript |
| 630 | 34 | (R34C) | GLRKRLRKFRNKIKEKLKKIGQKIQGLLPKLAPCTDY | 47.8 | DMSO | Genscript |
| 631 | 34 | (R34D) | GLRKRLRKFRNKIKEKLKKIGQKIQGLLPKLAPDTDY | 42 | DMSO | Genscript |
| 632 | 34 | (R34E) | GLRKRLRKFRNKIKEKLKKIGQKIQGLLPKLAPETDY | 58 | DMSO | Genscript |
| 633 | 34 | (R34F) | GLRKRLRKFRNKIKEKLKKIGQKIQGLLPKLAPFTDY | 57.7 | DMSO | Genscript |
| 634 | 34 | (R34G) | GLRKRLRKFRNKIKEKLKKIGQKIQGLLPKLAPGTDY | 48.8 | DMSO | Genscript |
| 635 | 34 | (R34H) | GLRKRLRKFRNKIKEKLKKIGQKIQGLLPKLAPHTDY | 64.4 | DMSO | Genscript |
| 636 | 34 | (R34I) | GLRKRLRKFRNKIKEKLKKIGQKIQGLLPKLAPITDY | 56.5 | DMSO | Genscript |
| 637 | 34 | (R34K) | GLRKRLRKFRNKIKEKLKKIGQKIQGLLPKLAPKTDY | 33.3 | DMSO | Genscript |
| 638 | 34 | (R34L) | GLRKRLRKFRNKIKEKLKKIGQKIQGLLPKLAPLTDY | 54 | DMSO | Genscript |
| 639 | 34 | (R34M) | GLRKRLRKFRNKIKEKLKKIGQKIQGLLPKLAPMTDY | 47.2 | DMSO | Genscript |
| 640 | 34 | (R34N) | GLRKRLRKFRNKIKEKLKKIGQKIQGLLPKLAPNTDY | 62.4 | DMSO | Genscript |
| 641 | 34 | (R34P) | GLRKRLRKFRNKIKEKLKKIGQKIQGLLPKLAPPTDY | 81.1 | DMSO | Genscript |
| 642 | 34 | (R34Q) | GLRKRLRKFRNKIKEKLKKIGQKIQGLLPKLAPQTDY | 52.3 | DMSO | Genscript |
| 643 | 34 | (R34S) | GLRKRLRKFRNKIKEKLKKIGQKIQGLLPKLAPSTDY | 35.5 | DMSO | Genscript |
| 644 | 34 | (R34T) | GLRKRLRKFRNKIKEKLKKIGQKIQGLLPKLAPTTDY | 48.2 | DMSO | Genscript |
| 645 | 34 | (R34V) | GLRKRLRKFRNKIKEKLKKIGQKIQGLLPKLAPVTDY | 68.1 | DMSO | Genscript |
| 646 | 34 | (R34W) | GLRKRLRKFRNKIKEKLKKIGQKIQGLLPKLAPWTDY | 55.6 | DMSO | Genscript |
| 647 | 34 | (R34Y) | GLRKRLRKFRNKIKEKLKKIGQKIQGLLPKLAPYTDY | 58.2 | DMSO | Genscript |
| 648 | 35 | (T35A) | GLRKRLRKFRNKIKEKLKKIGQKIQGLLPKLAPRADY | 52.1 | DMSO | Genscript |
| 649 | 35 | (T35C) | GLRKRLRKFRNKIKEKLKKIGQKIQGLLPKLAPRCDY | 29.6 | DMSO | Genscript |
| 650 | 35 | (T35D) | GLRKRLRKFRNKIKEKLKKIGQKIQGLLPKLAPRDDY | 58.4 | DMSO | Genscript |
| 651 | 35 | (T35E) | GLRKRLRKFRNKIKEKLKKIGQKIQGLLPKLAPREDY | 63.3 | DMSO | Genscript |
| 652 | 35 | (T35F) | GLRKRLRKFRNKIKEKLKKIGQKIQGLLPKLAPRFDY | 62.2 | DMSO | Genscript |
| 653 | 35 | (T35G) | GLRKRLRKFRNKIKEKLKKIGQKIQGLLPKLAPRGDY | 52.9 | DMSO | Genscript |
| 654 | 35 | (T35H) | GLRKRLRKFRNKIKEKLKKIGQKIQGLLPKLAPRHDY | 53.2 | DMSO | Genscript |
| 655 | 35 | (T35I) | GLRKRLRKFRNKIKEKLKKIGQKIQGLLPKLAPRIDY | 40.3 | DMSO | Genscript |
| 656 | 35 | (T35K) | GLRKRLRKFRNKIKEKLKKIGQKIQGLLPKLAPRKDY | 60.1 | DMSO | Genscript |
| 657 | 35 | (T35L) | GLRKRLRKFRNKIKEKLKKIGQKIQGLLPKLAPRLDY | 68.9 | DMSO | Genscript |
| 658 | 35 | (T35M) | GLRKRLRKFRNKIKEKLKKIGQKIQGLLPKLAPRMDY | 47.4 | DMSO | Genscript |
| 659 | 35 | (T35N) | GLRKRLRKFRNKIKEKLKKIGQKIQGLLPKLAPRNDY | 66 | DMSO | Genscript |
| 660 | 35 | (T35P) | GLRKRLRKFRNKIKEKLKKIGQKIQGLLPKLAPRPDY | 40.9 | DMSO | Genscript |
| 661 | 35 | (T35Q) | GLRKRLRKFRNKIKEKLKKIGQKIQGLLPKLAPRQDY | 53.9 | DMSO | Genscript |
| 662 | 35 | (T35R) | GLRKRLRKFRNKIKEKLKKIGQKIQGLLPKLAPRRDY | 48.1 | DMSO | Genscript |
| 663 | 35 | (T35S) | GLRKRLRKFRNKIKEKLKKIGQKIQGLLPKLAPRSDY | 43 | DMSO | Genscript |
| 664 | 35 | (T35V) | GLRKRLRKFRNKIKEKLKKIGQKIQGLLPKLAPRVDY | 62.5 | DMSO | Genscript |
| 665 | 35 | (T35W) | GLRKRLRKFRNKIKEKLKKIGQKIQGLLPKLAPRWDY | 65 | DMSO | Genscript |
| 666 | 35 | (T35Y) | GLRKRLRKFRNKIKEKLKKIGQKIQGLLPKLAPRYDY | 48.7 | DMSO | Genscript |
| 667 | 36 | (D36A) | GLRKRLRKFRNKIKEKLKKIGQKIQGLLPKLAPRTAY | 40.3 | DMSO | Genscript |
| 668 | 36 | (D36C) | GLRKRLRKFRNKIKEKLKKIGQKIQGLLPKLAPRTCY | 36.8 | DMSO | Genscript |
| 669 | 36 | (D36E) | GLRKRLRKFRNKIKEKLKKIGQKIQGLLPKLAPRTEY | 27.3 | DMSO | Genscript |
| 670 | 36 | (D36F) | GLRKRLRKFRNKIKEKLKKIGQKIQGLLPKLAPRTFY | 52.4 | DMSO | Genscript |
| 671 | 36 | (D36G) | GLRKRLRKFRNKIKEKLKKIGQKIQGLLPKLAPRTGY | 64.5 | DMSO | Genscript |
| 672 | 36 | (D36H) | GLRKRLRKFRNKIKEKLKKIGQKIQGLLPKLAPRTHY | 35.4 | DMSO | Genscript |
| 673 | 36 | (D36I) | GLRKRLRKFRNKIKEKLKKIGQKIQGLLPKLAPRTIY | 45.6 | DMSO | Genscript |
| 674 | 36 | (D36K) | GLRKRLRKFRNKIKEKLKKIGQKIQGLLPKLAPRTKY | 48.1 | DMSO | Genscript |
| 675 | 36 | (D36L) | GLRKRLRKFRNKIKEKLKKIGQKIQGLLPKLAPRTLY | 65.9 | DMSO | Genscript |
| 676 | 36 | (D36M) | GLRKRLRKFRNKIKEKLKKIGQKIQGLLPKLAPRTMY | 46.7 | DMSO | Genscript |
| 677 | 36 | (D36N) | GLRKRLRKFRNKIKEKLKKIGQKIQGLLPKLAPRTNY | 60.2 | DMSO | Genscript |
| 678 | 36 | (D36P) | GLRKRLRKFRNKIKEKLKKIGQKIQGLLPKLAPRTPY | 42.9 | DMSO | Genscript |
| 679 | 36 | (D36Q) | GLRKRLRKFRNKIKEKLKKIGQKIQGLLPKLAPRTQY | 37.9 | DMSO | Genscript |
| 680 | 36 | (D36R) | GLRKRLRKFRNKIKEKLKKIGQKIQGLLPKLAPRTRY | 41.5 | DMSO | Genscript |
| 681 | 36 | (D36S) | GLRKRLRKFRNKIKEKLKKIGQKIQGLLPKLAPRTSY | 48.1 | DMSO | Genscript |
| 682 | 36 | (D36T) | GLRKRLRKFRNKIKEKLKKIGQKIQGLLPKLAPRTTY | 57 | DMSO | Genscript |
| 683 | 36 | (D36V) | GLRKRLRKFRNKIKEKLKKIGQKIQGLLPKLAPRTVY | 64.5 | DMSO | Genscript |
| 684 | 36 | (D36W) | GLRKRLRKFRNKIKEKLKKIGQKIQGLLPKLAPRTWY | 48.2 | DMSO | Genscript |
| 685 | 36 | (D36Y) | GLRKRLRKFRNKIKEKLKKIGQKIQGLLPKLAPRTYY | 33.7 | DMSO | Genscript |
| 686 | 37 | (Y37A) | GLRKRLRKFRNKIKEKLKKIGQKIQGLLPKLAPRTDA | 57.5 | DMSO | Genscript |
| 687 | 37 | (Y37C) | GLRKRLRKFRNKIKEKLKKIGQKIQGLLPKLAPRTDC | 58 | DMSO | Genscript |
| 688 | 37 | (Y37D) | GLRKRLRKFRNKIKEKLKKIGQKIQGLLPKLAPRTDD | 56.5 | DMSO | Genscript |
| 689 | 37 | (Y37E) | GLRKRLRKFRNKIKEKLKKIGQKIQGLLPKLAPRTDE | 74.2 | DMSO | Genscript |
| 690 | 37 | (Y37F) | GLRKRLRKFRNKIKEKLKKIGQKIQGLLPKLAPRTDF | 34.3 | DMSO | Genscript |
| 691 | 37 | (Y37G) | GLRKRLRKFRNKIKEKLKKIGQKIQGLLPKLAPRTDG | 46.7 | DMSO | Genscript |
| 692 | 37 | (Y37H) | GLRKRLRKFRNKIKEKLKKIGQKIQGLLPKLAPRTDH | 51.8 | DMSO | Genscript |
| 693 | 37 | (Y37I) | GLRKRLRKFRNKIKEKLKKIGQKIQGLLPKLAPRTDI | 48.5 | DMSO | Genscript |
| 694 | 37 | (Y37K) | GLRKRLRKFRNKIKEKLKKIGQKIQGLLPKLAPRTDK | 59 | DMSO | Genscript |
| 695 | 37 | (Y37L) | GLRKRLRKFRNKIKEKLKKIGQKIQGLLPKLAPRTDL | 61.2 | DMSO | Genscript |
| 696 | 37 | (Y37M) | GLRKRLRKFRNKIKEKLKKIGQKIQGLLPKLAPRTDM | 29.3 | DMSO | Genscript |
| 697 | 37 | (Y37N) | GLRKRLRKFRNKIKEKLKKIGQKIQGLLPKLAPRTDN | 38.7 | DMSO | Genscript |
| 698 | 37 | (Y37P) | GLRKRLRKFRNKIKEKLKKIGQKIQGLLPKLAPRTDP | 60.8 | DMSO | Genscript |
| 699 | 37 | (Y37Q) | GLRKRLRKFRNKIKEKLKKIGQKIQGLLPKLAPRTDQ | 29.3 | DMSO | Genscript |
| 700 | 37 | (Y37R) | GLRKRLRKFRNKIKEKLKKIGQKIQGLLPKLAPRTDR | 53.2 | DMSO | Genscript |
| 701 | 37 | (Y37S) | GLRKRLRKFRNKIKEKLKKIGQKIQGLLPKLAPRTDS | 61.5 | DMSO | Genscript |
| 702 | 37 | (Y37T) | GLRKRLRKFRNKIKEKLKKIGQKIQGLLPKLAPRTDT | 51.4 | DMSO | Genscript |
| 703 | 37 | (Y37V) | GLRKRLRKFRNKIKEKLKKIGQKIQGLLPKLAPRTDV | 38.2 | DMSO | Genscript |
| 704 | 37 | (Y37W) | GLRKRLRKFRNKIKEKLKKIGQKIQGLLPKLAPRTDW | 58 | DMSO | Genscript |

**Cap18 derivatives with high purity (>95%)**

| Name | Position | Substitution | Amino Acid Sequence | Purity | Solvent | Company |
| --- | --- | --- | --- | --- | --- | --- |
| Peptide 1 | 6 | L6P | GLRKR**P**RKFRNKIKEKLKKIGQKIQGLLPKLAPRTDY | 99.9 | DMSO | Genscript |
| Peptide 2 | 10 | R10L | GLRKRLRKF**L**NKIKEKLKKIGQKIQGLLPKLAPRTDY | 100 | DMSO | Peptide 2.0 |
| Peptide 3 | 13 | I13D | GLRKRLRKFRNK**D**KEKLKKIGQKIQGLLPKLAPRTDY | 96.59 | DMSO | Peptide 2.0 |
| Peptide 4 | 13 | I13F | GLRKRLRKFRNK**F**KEKLKKIGQKIQGLLPKLAPRTDY | 100 | DMSO | Peptide 2.0 |
| Peptide 5 | 13 | I13H | GLRKRLRKFRNK**H**KEKLKKIGQKIQGLLPKLAPRTDY | 97.8 | DMSO | Genscript |
| Peptide 6 | 13 | I13M | GLRKRLRKFRNK**M**KEKLKKIGQKIQGLLPKLAPRTDY | 100 | DMSO | Peptide 2.0 |
| Peptide 7 | 13 | I13Q | GLRKRLRKFRNK**Q**KEKLKKIGQKIQGLLPKLAPRTDY | 95.85 | DMSO | Peptide 2.0 |
| Peptide 8 | 13 | I13S | GLRKRLRKFRNK**S**KEKLKKIGQKIQGLLPKLAPRTDY | 96.6 | DMSO | Genscript |
| Peptide 9 | 16 | K16C | GLRKRLRKFRNKIKE**C**LKKIGQKIQGLLPKLAPRTDY | 95.8 | DMSO | Genscript |
| Peptide 10 | 16 | K16D | GLRKRLRKFRNKIKE**D**LKKIGQKIQGLLPKLAPRTDY | 99.84 | DMSO | Peptide 2.0 |
| Peptide 11 | 16 | K16F | GLRKRLRKFRNKIKE**F**LKKIGQKIQGLLPKLAPRTDY | 98.1 | DMSO | Genscript |
| Peptide 12 | 16 | K16I | GLRKRLRKFRNKIKE**I**LKKIGQKIQGLLPKLAPRTDY | 95.6 | DMSO | Genscript |
| Peptide 13 | 16 | K16L | GLRKRLRKFRNKIKE**L**LKKIGQKIQGLLPKLAPRTDY | 100 | DMSO | Peptide 2.0 |
| Peptide 14 | 16 | K16M | GLRKRLRKFRNKIKE**M**LKKIGQKIQGLLPKLAPRTDY | 96.3 | DMSO | Genscript |
| Peptide 15 | 16 | K16Y | GLRKRLRKFRNKIKE**Y**LKKIGQKIQGLLPKLAPRTDY | 96.8 | DMSO | Genscript |
| Peptide 16 | 17 | L17D | GLRKRLRKFRNKIKEK**D**KKIGQKIQGLLPKLAPRTDY | 97.46 | DMSO | Peptide 2.0 |
| Peptide 17 | 17 | L17K | GLRKRLRKFRNKIKEK**K**KKIGQKIQGLLPKLAPRTDY | 96.84 | DMSO | Peptide 2.0 |
| Peptide 18 | 17 | L17P | GLRKRLRKFRNKIKEK**P**KKIGQKIQGLLPKLAPRTDY | 96.26 | DMSO | Peptide 2.0 |
| Peptide 19 | 18 | K18P | GLRKRLRKFRNKIKEKL**P**KIGQKIQGLLPKLAPRTDY | 99.32 | DMSO | Peptide 2.0 |
| Peptide 20 | 20 | I20E | GLRKRLRKFRNKIKEKLKK**E**GQKIQGLLPKLAPRTDY | 99.72 | DMSO | Peptide 2.0 |
| Peptide 21 | 20 | I20H | GLRKRLRKFRNKIKEKLKK**H**GQKIQGLLPKLAPRTDY | 95.2 | DMSO | Peptide 2.0 |
| Peptide 22 | 20 | I20N | GLRKRLRKFRNKIKEKLKK**N**GQKIQGLLPKLAPRTDY | 96.95 | DMSO | Peptide 2.0 |
| Peptide 23 | 21 | G21C | GLRKRLRKFRNKIKEKLKKI**C**QKIQGLLPKLAPRTDY | 95.3 | DMSO | Genscript |
| Peptide 24 | 21 | G21L | GLRKRLRKFRNKIKEKLKKI**L**QKIQGLLPKLAPRTDY | 95.3 | DMSO | Genscript |
| Peptide 25 | 24 | I24C | GLRKRLRKFRNKIKEKLKKIGQK**C**QGLLPKLAPRTDY | 96.1 | DMSO | Genscript |
| Peptide 26 | 24 | I24D | GLRKRLRKFRNKIKEKLKKIGQK**D**QGLLPKLAPRTDY | 98.99 | DMSO | Peptide 2.0 |
| Peptide 27 | 24 | I24G | GLRKRLRKFRNKIKEKLKKIGQK**G**QGLLPKLAPRTDY | 96.2 | DMSO | Genscript |
| Peptide 28 | 24 | I24N | GLRKRLRKFRNKIKEKLKKIGQK**N**QGLLPKLAPRTDY | 99.78 | DMSO | Peptide 2.0 |
| Peptide 29 | 24 | I24S | GLRKRLRKFRNKIKEKLKKIGQK**S**QGLLPKLAPRTDY | 98.94 | DMSO | Peptide 2.0 |
| Peptide 30 | 26 | G26T | GLRKRLRKFRNKIKEKLKKIGQKIQ**T**LLPKLAPRTDY | 95.05 | DMSO | Peptide 2.0 |
| Peptide 31 | 27 | L27P | GLRKRLRKFRNKIKEKLKKIGQKIQG**P**LPKLAPRTDY | 97.94 | DMSO | Peptide 2.0 |
| Peptide 32 | 29 | P29A | GLRKRLRKFRNKIKEKLKKIGQKIQGLL**A**KLAPRTDY | 98.63 | DMSO | Peptide 2.0 |
| Peptide 33 | 29 | P29D | GLRKRLRKFRNKIKEKLKKIGQKIQGLL**D**KLAPRTDY | 96.98 | DMSO | Peptide 2.0 |
| Peptide 34 | 29 | P29F | GLRKRLRKFRNKIKEKLKKIGQKIQGLL**F**KLAPRTDY | 97.2 | DMSO | Peptide 2.0 |
| Peptide 35 | 29 | P29H | GLRKRLRKFRNKIKEKLKKIGQKIQGLL**H**KLAPRTDY | 98.64 | DMSO | Peptide 2.0 |
| Peptide 36 | 29 | P29S | GLRKRLRKFRNKIKEKLKKIGQKIQGLL**S**KLAPRTDY | 96.23 | DMSO | Peptide 2.0 |
